# A Critical Analysis on Characterizing the Meditation Experience Through the Electroencephalogram

**DOI:** 10.3389/fnsys.2020.00053

**Published:** 2020-08-07

**Authors:** Camila Sardeto Deolindo, Mauricio Watanabe Ribeiro, Maria Adelia Aratanha, Rui Ferreira Afonso, Mona Irrmischer, Elisa Harumi Kozasa

**Affiliations:** ^1^Hospital Israelita Albert Einstein, São Paulo, Brazil; ^2^Department of Integrative Neurogenomics and Cognitive Research (CNCR), Amsterdam Neuroscience, VU Amsterdam, Amsterdam, Netherlands

**Keywords:** meditation, electroencephalogram, brain, mindfulness, contemplative science

## Abstract

Meditation practices, originated from ancient traditions, have increasingly received attention due to their potential benefits to mental and physical health. The scientific community invests efforts into scrutinizing and quantifying the effects of these practices, especially on the brain. There are methodological challenges in describing the neural correlates of the subjective experience of meditation. We noticed, however, that technical considerations on signal processing also don't follow standardized approaches, which may hinder generalizations. Therefore, in this article, we discuss the usage of the electroencephalogram (EEG) as a tool to study meditation experiences in healthy individuals. We describe the main EEG signal processing techniques and how they have been translated to the meditation field until April 2020. Moreover, we examine in detail the limitations/assumptions of these techniques and highlight some good practices, further discussing how technical specifications may impact the interpretation of the outcomes. By shedding light on technical features, this article contributes to more rigorous approaches to evaluate the construct of meditation.

## 1. Introduction

Meditation is a broad term that encompasses many practices; different styles and forms of meditation are present in almost all cultures and religions (Tang et al., 2015). Ancient traditional practices are still followed by a large number of practitioners, such as Buddhist meditation, Yoga, Tai Chi, and Qigong, although, new and secularized techniques, drawn from the context of eastern traditions, have become increasingly popular, such as modern variants of mindfulness practices (Kabat-Zinn, 1982). The number of practitioners has significantly increased in recent years due to meditation positive effects on stress reduction, well-being promotion, emotional regulation, attention control, and on cognitive performance (Barnes et al., 2008; Okoro et al., 2013; Tang et al., 2015; Khoury et al., 2017). The area has aroused a growing interest of the scientific community. Therefore, the usage of adequate scientific methodology and instruments to scrutinize the phenomena are of greatest importance.

There are many methodological challenges in the study of meditation, since it encompasses subjective features (Davidson and Kaszniak, 2015). The foremost consideration entangles the conceptualization of the phenomenon: up to now there is no universally applied definition of meditation that encompasses its complexity under the lenses of both scientific thinking and contemplative theories, although there are initiatives dealing with this issue (Nash and Newberg, 2013; Dahl et al., 2015). For example, Lutz et al. (2015) proposed a heuristic tool that describes the construct of meditation by incorporating both features of first-person experience, and making use of clinical frameworks and neuroscientific models. Moreover, there are other challenges, such as the impossibility of true double-blinding research designs, the selection of appropriate control groups, the limits of introspection, and the question to what extent one can rely on self-report measures (Davidson and Kaszniak, 2015).

This is of particular importance if we consider evidences pointing that meditation neural correlates vary with level of expertise, length and nature of practice (Davidson and Kaszniak, 2015; Lutz et al., 2015; Tang et al., 2015). For example, one may be interested on the state of mind and brain induced by the practice. The analysis of the so-called *state* effects may be performed in complete novices or in experienced practitioners. Regarding the former, it is still troublesome to objectively assert the level of compliance of the participant with the instructions, especially in such early stages of the practice. As for the latter, the meditation impact is dependent on the accumulation of previous effects of the practice, and, therefore, one evaluates not only state, but also the so-called *trait* effects, that remain even when participants are outside of formal practice. In this case, state and trait effects are interacting with one another. Thus, to summarize, it is still not clear up to what extent neural correlates meaningfully reflect meditation, and one should be extra careful with reverse inferences (Davidson and Kaszniak, 2015).

In spite of the aforementioned methodological questions, neuroscience research in meditation is nowadays a multidisciplinary field, encompassing contemplatives and scientists with distinct backgrounds, ranging from healthcare to the so-called hard-sciences. Given the multitude of backgrounds of the people on the field, it is paramount that new researchers can quickly get acquainted not only with the main findings, but also with the various available techniques at their disposal to best assess the phenomenon. Therefore, in this work, we shed light on complementary methodological issues permeating the field, regarding technical features. To this end, we unravel and discuss the usage of electroencephalogram (EEG) recordings in meditation studies until April 2020. Lomas et al. (2015) performed a systematic review of EEG findings on meditation and emphasized that the disparity of methodological approaches hinders the elaboration of a meta-analysis, which restricts the generalization of the conclusions. Therefore, in this review we cover in detail EEG signal processing methods that have already been translated to field, discussing assumptions, limitations and strengths of each technique, while also covering equipment enhancements over time. Furthermore, we list EEG technical specifications that must complement the existing guidelines on standardizing and designing EEG protocols on meditation.

## 2. Methods

In this work our emphasis is on the methodological approaches concerning EEG signal processing, although we describe the main findings in the literature. To this end, we analyzed the results in Pubmed for the query “*(neurophysiology OR electroencephalogram) AND (meditation OR mindfulness)*” up to April, 27th 2020. We limited this work to healthy individuals, due to further biases that may arise from illnesses effects. We further excluded studies which encompassed postural movements during the recordings (e.g., Yoga and Tai Chi), albeit they also might be classified as types of meditation (Ospina, 2007; Nash and Newberg, 2013), since body usage/movement manipulation may lead to confounding factors on EEG signals. Nevertheless, these studies were included when the subjects were still during EEG recordings. All the included studies had to be published and to be written in English.

The query resulted in 458 papers. Fifteen (15) were excluded for not being complete articles (e.g., abstracts, editorial letters, etc.), and 48 for being reviews. Moreover, three (3) were excluded for evaluating teenagers, children or infants and one (1) for being an animal model. From the scan of the papers, 43 were excluded for not having EEG, 68 for evaluating patients and 81 for not reporting meditation. Furthermore, as the result of group discussions and analyses, 13 were excluded due to lack of quality, nine (9) for incorporating movements during meditation or not specifically stating that the recordings were performed when participants were still, two (2) for being only study protocols and three (3) for referring to pain evaluation. We excluded a total of 286 studies, and added 15 more during reading, that were absent in the search query, resulting in a total of 187 papers included in the present review.

### 2.1. Categories of Meditation

The nature of the meditation practice has impacts in the neural correlates, and there is still controversy regarding how to group and differentiate among practices (Lutz et al., 2008; Nash and Newberg, 2013; Dahl et al., 2015; Davidson and Kaszniak, 2015). According to Nash and Newberg (2013), there are two main approaches to define “meditation” in the literature: while the first sees meditation as a family of mental training techniques—the “method definition,” the second—“state definition”—is more concerned about experiential/altered states of consciousness. In a systematic review of mindfulness meditation and EEG findings, Lomas et al. (2015) used the proposal of Lutz et al. (2008) (a “method definition approach” Nash and Newberg, 2013), that divides the practices into two broad categories: (i) Focused attention (FA), encompassing a pool of practices aimed at sustaining the focus on a particular object, and that gradually improve concentration and the ability to notice distractions; and (ii) Open monitoring (OM) that assumes no specific focus, but a rather non-specific monitoring of the experiences arising in the mental continuum. However, it is important to emphasize that these categories are not dissociated, and they may share cognitive components with one another: in some traditions OM is introduced after the practitioner has achieved some stability of attention regulation through FA (Davidson and Kaszniak, 2015). Another largely used category is Non-dual awareness (NDA), which does not encompass a dualistic orientation, i.e., that there is a subject observing an object, but rather a level of awareness free of these constraints (Josipovic, 2010). Nevertheless, Travis and Shear (2010) argue that Vedic and Chinese traditions do not fit in these categories, and proposed an additional one: Automatic self-transcending (based in the “state definition approach” Nash and Newberg, 2013), that hypothesizes an explanation to what happens when transitioning from one state of consciousness to another.

The aforementioned disagreements in meditation taxonomy make difficult to unify all practices within definite categories. Therefore, in this paper, we divide meditations based on the proposal of Nash and Newberg (2013), that attempts to unify practices using a third-person perspective in three categories: (i) Affective-directed methods (ADM), which relate to an enhanced affective state during meditation [e.g., Love and Kindness (LK) meditation]; (ii) Null-directed methods (NDM), which attempt a state that is neither affective nor cognitive, thus devoid of a phenomenological content, where the practitioner is unable to define a notion of self or differentiate self and exterior [e.g., Transcendental Meditation (TM), Zen satori and some Yoga methods]; and (iii) Cognitive-directed methods (CDM), which encompass techniques focusing on developing and stabilizing enhanced cognitive states, such as one-pointedness, mindfulness, or insight. This theoretical construct shares similarities with the model of attentional (cognitive-directed) and constructive (affective-directed) categories of practice proposed by Dahl et al. (2015). We have further subdivided CDM when possible in FA and OM, due to the broad usage of these terms and for the sake of similarity with the systematic review of Lomas et al. (2015). Indeed, in another review on meditation and EEG, Lee et al. (2018b) employ four categories OM, FA, TM, and LK, which largely resembles our proposal for this work.

## 3. The Electroencephalogram: An Overview

EEG recordings have been used as a measurement tool to assess meditation given their ability to describe correlates in the central nervous system. In addition, it is non-invasive, widely used in clinic practice and of relative low-cost. The EEG signal is complex and admits a myriad of signal processing approaches that can be explored to evaluate the meditation phenomenon. EEG in humans was first registered by Berger (1935). He performed a systematic study on the brain electrical activity through scalp electrodes connected to a galvanometer (an electromechanical device that measures electric current). He further described various signal patterns noticeable in healthy subjects and in patients. One of the main features was named *alpha wave*, emerging on the occipital area when the subject had their eyes closed.

The electrical signal detected on the scalp is a direct and robust measurement of the synchronized activity of a myriad of neurons in the brain (Nunez and Srinivasan, 2006). The resultant electric field radiates through the biological tissue, such that the farther away from the current source, the greater the attenuation in amplitude and the greater the coverage area. This phenomenon is known as *volume conduction*, and consequently, each EEG electrode measures not only the activity from its vicinity, but also from distant cortical areas and other electrical sources (Biasiucci et al., 2019). Thus, a source may potentially affect multiple electrodes, and there is an intrinsic correlation between the recordings of some EEG channels (Schomer and Lopes da Silva, 2011; Michel and Koenig, 2018; Biasiucci et al., 2019). This make the interpretation of oscillatory activity challenging. We also emphasize that resembling patterns of EEG activity cannot be used to infer that participants have the same mental states.

Therefore, being a direct measure of electrical activity, the EEG has a high temporal resolution, i.e., signal changes are seen almost in real time (with milliseconds precision). Nevertheless, it has, in general, a lower spatial resolution in comparison to other neuroimaging techniques. In other words, a large number of neurons have to modulate their activity in order to be detected by this instrument, but these responses are observable with very low latency. The aforementioned characteristics make the EEG a suitable device for studying short-lasting functional connections between distinct brain sites that emerge due to specific events. The EEG is a combination of waves that show higher power in the frequencies ranging from 1 to 30 Hz. Frequencies are divided into bands, but their specific boundaries are not a consensus in the literature (Buzsáki, 2006), and there are some proposals to establish the boundaries by taking into consideration the specificities of each individual (Klimesch, 1999). The most common ranges are known as: Delta (0–4 Hz Banquet, 1973; Badawi et al., 1984; Dunn et al., 1999; Cahn et al., 2010), Theta (4–8 Hz Banquet, 1973; Badawi et al., 1984; Dunn et al., 1999; Cahn et al., 2010), Alpha (8–14 Hz Banquet, 1973; Badawi et al., 1984; Dunn et al., 1999; Cahn et al., 2010), Beta (14–35 Hz Dunn et al., 1999; Cahn et al., 2010), and Gamma (35–45 Hz Lutz et al., 2004; Tei et al., 2009; Cahn et al., 2010. However, some studies consider frequencies up to 200 Hz as gamma Fell et al., 2010). The rhythms are linked to many behavioral outcomes, but we emphasize that cognitive tasks are accomplished not by one specific rhythm, but an assemble of spectral frequencies and that the measured rhythms may vary depending of the recording technique and on the choice of reference. The most relevant rhythms for meditation studies are described in the next section.

By the time the first studies on EEG changes due to meditation started around the 60's (Anand et al., 1961; Kasamatsu and Hirai, 1966), analog EEG systems were still present on the market (Collura, 1993). In general, analog circuits dissipate more heat; are larger in size; and have higher susceptibility to noise, which is burdensome when one is interested in low energy signals, typical in EEG. Moreover, these EEG apparatuses relied on analog pen-based systems, and signal amplitude variations were stored in long paper ribbons. EEG analysis was then performed by visual inspection. Nevertheless, the incorporation of digital EEG apparatus was not instantaneous, with analog EEG setups persisting until the 80's (Collura, 1993). The incorporation of digital recordings and their storage in tapes allowed further conclusions regarding the neuronal correlates of meditation.

*Artifacts*, corresponding to spurious electrical activity, i.e., recorded signals not derived from cortical activity, are an important aspect of the EEG analysis. The most common and also hard to remove artifacts are muscular activities of head and neck. They share the same bandwidth as typical neuronal signals but have a much greater amplitude. The muscular activity of the eyes also produces a well-known signature in the EEG recordings (Hillyard and Galambos, 1970; Girton et al., 1973). Moreover, experiments are prone to equipment noise, cable movements, and electrodes misplacement (Islam et al., 2016). Throughout the years, scientists have developed different ways to handle artifacts and thus remove non-cortical activities incorporated into the signal, ranging from simple bad epoch exclusion to complex algorithms (Belouchrani et al., 1997; Shao et al., 2009; Winkler et al., 2011; Saggar et al., 2012; Reis et al., 2014). Nowadays, most EEG signals are bandpass filtered thus lessening high frequency noise and DC signal levels. Sometimes a notch filter is further incorporated, to reduce the hum noise (50 or 60 Hz). In addition, eyeblink artifacts are attenuated, for example, by the simultaneous acquisition of an eye electrode or by the Independent Component Analysis (ICA) algorithm (Islam et al., 2016).

## 4. Spectral Features

Although there are many reports based on visual inspection, the first studies on meditation mostly used spectral analysis on EEG signals, which corresponds to the signal characterization in the frequency domain. The decomposition of the EEG signal in specific frequency bands, followed by spectral analysis has expanded the ability to describe neuronal activity. The visualization of some variations, especially in higher frequencies, are only possible in the frequency domain. One algorithm is the *Fast Fourier Transform* (FFT), employed in some of the first studies done in the 70's and is still largely used.

Those studies reported variations in spectral characteristics of EEG signals: at the onset of meditation, the alpha waves increased in amplitude (Wallace et al., 1971; Banquet, 1973; Corby et al., 1978) and slightly reduced in frequency (Kasamatsu and Hirai, 1966; Wallace, 1970; Wallace et al., 1971; Banquet, 1973). Nevertheless, Stigsby et al. (1981) and Heide (1986) reported no difference in alpha activity between meditators and controls. Moreover, those studies reported the appearance of rhythmical theta trains/bursts in some of the meditators (Wallace et al., 1971; Banquet, 1973; Fenwick et al., 1977; Hebert and Lehmann, 1977; Warrenburg et al., 1980) and observed a positive relationship between experience in meditation and theta waves (Kasamatsu and Hirai, 1966; Elson et al., 1977; Corby et al., 1978). Some remarks were also reported on beta bandwidths (Banquet, 1973), but were not always reproducible (Stigsby et al., 1981). Interestingly, opposed to expectations, alpha wave variations were observable even when subjects had their eyes open (Kasamatsu and Hirai, 1966; Wallace, 1970). We recall that the majority of these studies were classified as NDM, but that the findings are in accordance with those of Kasamatsu and Hirai (1966), who analyzed a CDM practice.

More alterations of alpha waves in meditators can be related to *alpha-blocking* (Young and Fenton, 1971), first described in experiments when the subjects received a light influx as they had the eyes opened, however alpha waves appear faster and of smaller amplitude if the stimulus is repeated with periodicity. This phenomenon was further evaluated and the conclusions were controversial: generally, the habituation of the subjects to sensory stimuli has been reported to occur after many stimuli repetitions, so that the latency and amplitude of alpha waves decrease. However, Kasamatsu and Hirai (1966) reported that during meditation (Zazen—CDM-FA), contrary to expected, characteristic latency and amplitude remained fairly constant even after many stimuli repetition, a response that was associated with a lack of habituation. Thus, the lack of alpha-blocking was interpreted as an indication of a distinct state of consciousness, or sustained awareness. The result was partially corroborated by Williams and West (1975), who reported smaller alpha blocking habituation in TM practitioners. In contrast, Heide (1986) could not reproduce group differences using TM and neither could Becker and Shapiro (1981) with Zen, Yoga, and TM meditators, whereas Wallace (1970) reported the lack of alpha blocking habituation as an “occasional” phenomena.

It is important to note that, in early studies many EEG analyses were used to characterize the meditation state by specifically stating its differences or similarities with other states of consciousness, such as hypnosis (Tebēcis, 1975; Morse et al., 1977), relaxation (Elson et al., 1977; Morse et al., 1977; Warrenburg et al., 1980), and sleep (Hebert and Lehmann, 1977; Stigsby et al., 1981). Tebēcis (1975) described similarities of mean EEG parameters between TM practitioners (eyes closed) and hypnotized subjects, namely, a significantly higher mean theta density, during both TM and non-meditation. Nevertheless, the hypnosis group presented more theta than the TM group. Morse et al. (1977) corroborated part of these findings, and additionally reported no differences in slow alpha between hypnotized and relaxed subjects compared to meditators. Warrenburg et al. (1980) compared meditation and relaxation, reporting epochs of sleep stages 1 and 2 and that some subjects in both groups presented theta bursts in the EEG. Elson et al. (1977) reported that half of the control group fell asleep during relaxation, whilst none of the meditators did. Furthermore, they emphasized that even though alpha spectral signatures were similar between meditation and relaxation, the EEG signature was more stable in meditation, generally not developing into sleep stage 2 patterns. Pagano et al. (1976) reported wave forms classifiable as sleep stages (sleep onset) during 40–50% of the meditation time and Hebert and Lehmann (1977) reported drowsiness in about 10% of meditation time. Fenwick et al. (1977) found no significant difference between meditation and sleep onset. On the other hand, Stigsby et al. (1981) described that the EEG of TM practitioners were not different between wakefulness and drowsiness, but clearly distinct from sleep onset and the following sleep recordings. All these studies evaluated TM, except those from Elson et al. (1977), with Ananda Marga, which we also classified as NDM.

Studies also evaluated whether the observable neural correlates were not influenced by external stimuli, such as other cognitive processes or the effect of respiration. In this direction, meditation was compared with mental calculations by Akers et al. (1977) and by Severtsen and Bruya (1986) with aerobic exercises. Both studies were classified as CDM meditations and reported no group differences.

Finally, we remark that EEG signals are artifact prone, and in those early studies the solution was to limit data analysis to artifact-free epochs, which tend to be small data segments and neglect signal dynamics over time. Furthermore, due to technical limitations, studies tended to employ few electrodes. Many papers from that period dealing with meditation do not fully specify the EEG equipment used. Moreover, the literature covered several meditation techniques, such as TM (Wallace et al., 1971), zazen (Kasamatsu and Hirai, 1966), Ananda Marga (Elson et al., 1977; Corby et al., 1978), Raj Yoga (Anand et al., 1961), and catholic-oriented meditation (Akers et al., 1977)—even though most of the studies have been classified as NDM practices (refer to Supplementary Material). Given the lack of standard experimental protocols, the distinct qualities of technical equipment, some result inconsistencies as well as the great variability among subjects, the ability to perform complex analyses and generalize the findings is hindered and open to criticism.

The description of spectral features in the study of meditation persisted over time, primarily through the FFT analysis. Even though more enhanced equipment were employed and many times the spectral analysis was combined to other processing techniques, several studies continued assessing meditation using study designs similar to the first studies. Moreover, we emphasize that studies focusing on CDM meditation types became more frequent with time.

Remarkably, the concern on scrutinizing the different forms of meditative states persisted, and a number of studies distinguish them by means of spectral correlates. Travis and Wallace (1997) evaluate three phases of TM (NDM), reporting that the most advanced one was not achieved by all participants and that the least advanced had lower alpha and increased delta powers. This result is in accordance with the study by Travis (2001), that reported increased alpha amplitudes in TM-Siddhi, a more advanced type of TM practice. Also, Lee et al. (1997) corroborated these findings in distinct Qigong practices, classified as CDM-FA, by reporting increased alpha power in occipital regions during both a sound exercise and meditation. De Los Angeles et al. (2016) evaluated five states of Buddhist concentrative meditation (CDM-FA) and reported alpha power increases at all depth levels of meditation in comparison to rest, but in deeper states the authors further observed increased power in theta and decreases in central beta and low gamma. These differences were not observed in control participants. Tsai et al. (2013) analyzed, in one specific participant, progressive stages of meditation that can be classified as both of the aforementioned categories: CDM-FA and NDM, reporting bilateral alpha and theta increased activities in the more advanced practice. When the practices were compared to baseline, the least advanced meditation had higher bilateral activity only in the theta band, in contrast to the more advanced one, which activated both bandwidths. By contrast, Berman and Stevens (2015) evaluated CDM-FA/OM and NDM meditators in enriched consciousness states, reporting lower power in delta, theta and alpha during these states in comparison to baseline. Finally, Rodin et al. (2017) explored the brain InfraSlow Activity (0.0002–0.1 Hz) activity during YogaNidra (NDM), suggesting higher activity of these frequencies during attentive tasks, such as meditation.

Furthermore, there are comparisons of the meditation spectral signature with other cognitive activities, in which it is noticeable that findings in the alpha and theta bandwidths remain. Dunn et al. (1999) compared a concentrative practice with focus on the breath with Mindfulness (CDM-FA/OM), and reported not only increases in alpha and theta, but also in delta and beta 1 (13–25Hz). Partially corroborating these results, Bing-Canar et al. (2016) reported an increase in alpha power when comparing a group of guided Mindfulness meditators with a control audio group. Moreover, Baijal and Srinivasan (2010) contrasted Sahaj Samadhi (CDM-FA) with relaxation, reporting increased theta during deep meditation. Travis et al. (2001) also reported alpha power increase in TM meditation (NDM) in comparison with reading a modern language. Nevertheless, the increase was also noticeable when participants read Sanskrit. Aftanas and Golosheykin (2005) compared Sahaja Yoga practitioners (CDM-OM) with controls at baseline and each group was watching neutral or emotional movie clips. Their results indicate that the practice of meditation leads to a greater power in theta and alpha during resting, and that alpha activity remained elevated when they viewed neutral movie clips. This was interpreted as indicative that meditators show less tonic arousal and more internalized attention. Similarly, Lee et al. (2015) reported increased alpha (in frontal and central regions), but reduced gamma (in central regions) when Tibetan Nyingmapa meditators (CDM-FA) visualized emotional figures in comparison to a baseline condition. Also, Henz and Schöllhorn (2017) evaluated epochs of resting state in the following conditions: (i) before and after a session of Qigong; (ii) after mentalization of a movement sequence as performed during physical practice; and (iii) after seeing a video of practitioners performing Qigong. Theta and alpha-1 (8–10 Hz) increased after the practice and mentalization in comparison to watching the video, whereas beta and gamma were reduced. Given the similarity between mental and physical training, the results suggest that both induce relaxation of the mind. Finally, Takahashi et al. (2005) compared Zen (Su-soku, a CDM-FA practice) meditation with synchronized breath in novices, reporting increased power in alpha and theta. Their results are partially corroborated by Park and Park (2012), who employed paced breathing as a meditation protocol in comparison to baseline activity. The authors also report increased alpha power with diminished theta.

In addition to that, we recall the large amount of studies that compared meditators with controls/baseline and also reinforced the prevalence of findings in the alpha and theta bandwidths in a myriad of meditation techniques, even though they were not necessarily reported simultaneously in all studies. For example, Kubota et al. (2001), Tanaka et al. (2014), and Tomljenović et al. (2016) reported an increase solely in the theta band when respectively comparing Zen Su-soko (CDM-FA), Mindfulness (CDM-OM), and TM (NDM) practices with baseline. In contrast, Hinterberger et al. (2016) reported that Theta healers (CDM) presented a decreased theta power when compared with baseline, while their clients had no change. This lack of change in beginners is corroborated by Harne and Hiwale (2018) who reported no spectral changes after one session of “Om” chanting (CDM-FA).

On the other hand, some studies have reported only alpha band alterations, such as Qin et al. (2009), with Qigong (CDM-FA); Kim et al. (2013), with autogenic meditation (CDM-FA); and Fan et al. (2014) with a CDM meditation protocol. Nevertheless, many studies report increases in the power of both bandwidths, such as Arambula et al. (2001) with Kundalini Yoga (CDM-FA), Takahashi et al. (2005) with Zen (Su-soko), Lagopoulos et al. (2009) with Acem meditation (NDM), Lee et al. (2015) with Tibetan Nyingmapa (CDM-FA), Nair et al. (2017) with Brahma Kumaris Rajayoga (CDM), Sharma et al. (2018) with Rajyoga (ADM), and Kakumanu et al. (2018) with Vipassana practitioners (CDM and ADM). Still, there are noticeably findings in other bandwidths: Dentico et al. (2018) evaluated meditators before and after long CDM and ADM practices reporting an enhanced activity in frequency bands ranging from delta to low-gamma over midline prefrontal and left centro-parietal electrodes, with the higher spatial extent in the theta range. Tanaka et al. (2015) reported that open monitor meditators (CDM-OM) had lower frontal power in beta during both meditation and baseline. For the delta band, Kim et al. (2014) reported an increase solely in this bandwidth when comparing autogenic meditation (CDM-FA) with baseline, Baijal and Srinivasan (2010) also reported increases not only in these frequencies, but also in alpha for Sahaj Samadhi (CDM-FA). On the other hand, Gao et al. (2016) reported a decrease in delta and an increase in alpha in Mindfulness Based Stress Reduction (MBSR) practitioners (CDM with components of OM and FA). Cahn et al. (2010) further confirmed a decreased delta activity, but also pointed to increased theta and gamma activities in Vipassana (CDM-OM) practitioners. In agreement to reports of increased gamma activity in meditation, Lutz et al. (2004) reported increased amplitude and synchrony in this bandwidth for Loving and kindness (ADM) long-term practitioners, corroborated by Kakumanu et al. (2018) in Vipassana practitioners (CDM and ADM); by Braboszcz et al. (2017) in Vipassana, Himalayan Yoga and Isha Shoonya meditators (CDM-FA/OM); and by Fucci et al. (2018) in Open Presence and Focused attention practitioners (CDM-FA and NDM). However, Braboszcz et al. (2017) reported a positive correlation between gamma power during mind wandering and hours of meditation expertise, while Fucci et al. (2018) reported no relationship between spontaneous gamma activity and hours of meditation practice in expert practitioners. Yet, De Los Angeles et al. (2016) reported a decrease in central low-gamma in CDM-FA practitioners.

Regarding new processing approaches that were translated to the meditation field, firstly we mention the calculation of asymmetry between distinct hemispheres, more specifically in the anterior scalp. Frontal asymmetry in baseline activity was related to dispositional mood, affective reactivity and temperament. More specifically, the higher activities in the left frontal regions were associated to the arousal of positive and approach-related emotions, while the right frontal, to negative and withdrawn-related ones (Davidson, 1992). Davidson et al. (2003) performed a randomized controlled trial with the MBSR (CDM-FA/OM), reporting an anterior activation in alpha asymmetry as a function of meditation training. Moynihan et al. (2013) further evaluated the same meditation in elderly practitioners, and reported a leftward alpha asymmetry. In addition to that, Sharma et al. (2018) reported alpha asymmetry not only in frontal but also in parietal electrodes in experienced Rajyoga practitioners (ADM) when compared to controls. Travis and Arenander (2006) studied TM, a NDM practice and reported alpha frontal asymmetry in long term practitioners, but could not reproduce the findings in a 1 year longitudinal study, which, according to the authors, indicate that this measure might not be sufficiently sensitive to characterize the effects of some practices. Nevertheless, some authors recall asymmetry in other bands, like Chan et al. (2008) who pointed to a leftward theta asymmetry in both a ADM meditation and in music listening in comparison to a baseline recording.

Another advance in spectral analysis relates to the proposal of not restricting spectral bands at fixed intervals, but instead customizing them for each study subject. The alpha rhythmic figures within the dominant frequencies in the scalp of human adults, leading to a characteristic peak in this bandwidth on the spectrogram. However, the central frequency of this bandwidth varies to a large extent with the age and with the cognitive state of an individual. Therefore, the proposal of the *Individual alpha frequency* (IAF), defines the bandwidth in terms of either the peak or the “gravity” frequency, enabling more accurate estimates of modulations in alpha activity (Klimesch, 1999). Aftanas and Golosheikin (2003) reported that long-term CDM meditators had lower IAF relative to short-term practitioners, but Hinterberger et al. (2014) could not find similar differences between a group of experienced CDM meditators and a novices group; this finding was interpreted as indicative that the experienced practitioners are capable of remaining at rest without meditating. Saggar et al. (2012) applied this measure in EEG acquired on the course of 3-months CDM-FA retreats. They averaged IAF measures for all channels of each participant, reporting a reduction in this parameter in both intervention and waiting list groups. Furthermore, they used IAF to compute individual rhythmic bandwidths and, by means of this definition, the expected reductions in alpha-band power following training were not observed. They were, however, prominent in the beta band and the authors argue that the IAF-defined beta-band likely included activity from both the traditionally-defined alpha and beta bands. In a posterior work, Saggar et al. (2015) further employed spectral data from this experiment to fit and improve a computational model of cortical activity, that modeled the interactions between the cortex and the thalamus (Robinson et al., 2001). More specifically, the authors reported reductions in model parameters related to the transmission delay between modeled cortical and thalamic cells, which predicted the reduced IAF in experienced practitioners and reinforced that meditation training leads to changes in neuronal dynamics.

Furthermore, we observed that some studies have also incorporated spectral analysis with the *Wavelets Transform*. Roughly, the FFT has no time resolution and a high spectral resolution, but encompasses only stationary signals; whilst the wavelets transform encompasses non-stationarities, with the drawback of inserting higher uncertainties in the frequency domain. However, it is very good in localizing frequency variations in time, with a higher precision. Again, the findings are predominantly in alpha and theta. For example, Ren et al. (2011) used wavelets and reported an increased percentage of alpha, associated with a more relaxed mental state, in Susoku meditators (CDM-FA), while Nyhus et al. (2019) used the Morlet wavelet in computing the spectral power and reported an increase in theta power in frontal and left-parietal channels after a mindfulness meditation training (CDM-FA). Brandmeyer and Delorme (2016) also used the Morlet Wavelet, and compared the oscillations of mind wandering and meditation. Participants with different levels of expertise in Himalayan Yoga have been evaluated during their seated meditation, returning increased amplitudes in theta on frontal midline and in alpha on the somatosensory areas of meditators. These findings were followed by increased length of self-reported deep meditation epochs. Similarly, non-experts reported longer mind wandering episodes. The results corroborate the hypothesis that internal and external attentional processes share neuronal mechanisms, which modulate via meditation practice. Chandra et al. (2017) also applied Morlet wavelet and the Discrete Wavelet Transform (DWT) to obtain spectral features to differentiate low and high mental workload levels in Sudarshan Kriya Yoga (CDM/FA) practitioners and in a control group. They reported increase in alpha and beta energies in the group of meditators.

Another similar approach is related to the *Hilbert Transform*, which has similar properties to the FFT for longer signals, but that otherwise disposes of higher temporal resolution for fast dynamical variations in frequency, phase and amplitude; and it is usually done in combination with band-pass filters (Freeman, 2007). Ravnik-Glavač et al. (2012) employed a method based on the Discrete Hilbert Transform called Complex demodulation (Draganova and Popivanov, 1999) to compute the spectral power of two experienced CDM-FA meditation practitioners. Both practitioners showed increased power in the alpha and theta bandwidths.

In summary, the spectral results point to modulations mostly in the alpha and theta bandwidths, although there are reports in other frequencies. In particular, we emphasize the findings in gamma and we believe some pipelines would benefit of the usage of *Multitapers* to further detail estimates in this bandwidth, given that sometimes gamma bursts are not time locked with one another. Moreover, in our assessment, these spectral features emerge albeit the great diversity of meditation expertise and the use of distinct practice categories. Furthermore, the findings still point to these bandwidths even though many studies compared meditation with a wide variety of other cognitive activities. Thus, there is evidence that modulations in alpha and theta are robust and strong neuronal correlates of the meditative activity.

## 5. Evoked Potentials/Event Related Potentials

Due to the advances in digital data gathering, the study of evoked potentials (EP)/event related potentials (ERP) emerged as new analysis method, complementary to spectral analysis. The distinction between them is that EP are spontaneously evoked, in contrast to ERP which are elicited by cognitive task processing (Singh Nilkamal and Telles Shirley, 2015). In EP/ERP a characteristic and reproducible waveform, that is time-locked with an external or internal stimulus, is recorded from the scalp (Chiappa, 1997; Pfurtscheller and Da Silva, 1999). A straightforward method to detect and distinguish them from noise levels is to present repeated stimuli (trials) and average the trials' response, separately for each electrode (Bressler and Ding, 2006). The EP/ERP study comprises the identification of components in the average response (negative or positive wave peaks), noticeable before or after an event which can be of a sensory, motor or cognitive nature (Bressler and Ding, 2006; Sur and Sinha, 2009). The main waveform parameters that are generally evaluated in these analyses are *amplitude*, which is indicative of increased neuronal activity of the underlying generators, and *latency* of peaks, which relate to delayed transmissions of the corresponding neuronal generators. However, some studies have further analyzed the spectral components of these waveforms (Pasquini et al., 2015; Bing-Canar et al., 2016). As examples, we mention the P300 component, a positive peak around 300 ms after a stimulus and the N200, a negative peak around 200 ms after the event. EP/ERP analyses have proven robust throughout time, and are widely used to characterize meditation. In fact, they have been reported from the seventies up to the present days. Given that ERP experiments evaluate the response to stimuli, most experiments did not gather EEG simultaneously to the execution of meditative practices. Therefore, in these studies the main objective is to describe meditation effects that remain outside of the practice. Unless explicit, the reader shall consider that the studies in this section compare the ERP responses of meditators before and after practice. By contrast, most EP studies compare subjects at meditation with a baseline or with a control group.

### 5.1. Evoked Potentials

Auditory evoked potentials (AEP) figure within the first EP used in meditation research in the late seventies, and characterize how an involuntary response to external auditory stimuli may be influenced by the practice. The components related to AEP can be subdivided according to their latencies and each can be associated to distinct neuronal generators (for more information, the reader is referred to Singh Nilkamal and Telles Shirley, 2015): (i) short latency (SL), corresponding to waves I-V (1.9–5.8 ms), Na (14–19 ms), and Pa (25–32 ms), that mostly relate to inflection points up until 30 ms; (ii) mid latency (ML), including components Nb (35–65 ms), P1 (40–60 ms), and N1 (80–115 ms), whose inflections position in the range from 35 to 115 ms; and (iii) Long latency (LL), that include P2 (140–180 ms) and N2 (220–280 ms) and refer to characteristic inflections after 140 ms.

McEvoy et al. (1980) studied SLAEP (waves I, II, III, and V) before and after meditation in TM (NDM) practitioners and reported no latency differences in low intensity stimulus (0–35 dB), an increase in wave V during moderate intensity (40–50 dB), and a decrease at higher intensity ones (55–70 dB). Given that increased latencies were in the range of background noise (40–55 dB), their results corroborated with behavioral observations in meditators, namely, increased auditory acuity and decreased sensory thresholds. In contrast, Kumar et al. (2010) only reported a decrease in the latency of wave V during control sections (lecture on meditation/non-targeted thinking) and during a focused attention on the symbol “Om,” but there was no change during an effortless single thought on “Om.”

Subramanya and Telles (2009) compared SLAEP and MLAEP in cyclic meditation (CDM-FA) after practice with rest in supine position. They reported increased latencies for positive (Pa)/negative (Nb) components and increased amplitude for the negative (Nb) component. Telles et al. (1994) also studied SLAEP and MLAEP and likewise observed augmented amplitude of the negative component (Nb), but in this case, an experienced group during the “Om” meditation (CDM-FA) was compared with a “one” repetition-sham-meditation by a novice control group. Telles et al. (2012) studied the same type of practice, but contrasting it to baselines before and after sessions and also with non-meditation sessions. However, they reported increased amplitudes in the Pa component and increased Na/Pa but decreased Nb latencies. In a further study, Telles et al. (2015) complemented those previous results, by studying MLAEP e LLAEP components, reporting a reduced peak latency of the P2 during and after meditation, suggesting that meditation improves the processing of auditory information. This result contrasts with some of the first studies on LLAEP: Barwood et al. (1978) found no significant differences in N1, P1, and N2 latencies and amplitudes between subjects at TM, baseline (before and after meditation) and during light sleep. Corby et al. (1978) contrasted the response of Ananda Marga practitioners (NDM) and controls at baseline, in a preparatory condition and at meditation, reporting that P2, P3, and N1 amplitudes progressively decreased for infrequent tones as individuals entered meditation, but P2 and P3 increased for frequent ones. The different behavior of P2 and P3 was speculated to be related to the lack of habituation or to an active withdrawn of attention. However, Becker and Shapiro (1981) studied Zen, Yoga, and TM practitioners (all classified as NDM) at meditation and baseline, and in comparison to a control group, reporting no differences in P2 and P3 amplitudes while observing an increase for N1 in Yoga and TM.

Likewise to the auditory stimuli, there are also studies exploring visual evoked potentials (VEP), assessing whether the practice of meditation also interferes in other sensorial domains. We recall that the latencies in visual EP are generally higher in comparison to auditory ones. For example, Zhang et al. (1993) analyzed light flashes before, during and after Qigong meditation (Dantian, CDM-FA), observing ambiguous results: there was an increase in the VEP amplitudes (N80, P115, N150, P200, N280) during meditation in advanced practitioners, which was not observed in novices and beginners. However, in the same study there was a group of Qigong practitioners of other schools, which presented a significant decrease in the VEP amplitudes. However, Schöne et al. (2018) observed modulations in much higher latency waveforms (500–6,800 ms), reporting reduced VEP amplitudes acquired during multiple object tracking paradigms in the group that performed a training in mindful breath awareness (CDM-FA/OM) contrasted to an active control group, trained in muscular relaxation. The results were related to an enhanced ability to ignore irrelevant stimuli and with lower attentional effort after meditation training.

Since EP analysis rely on signal averaging around an event, it is highly dependent on the onset, number of repetitions, as well as type (e.g., auditory or visual) of stimuli. Although most results suggest that meditation has an effect on the processes underlying the generation of endogenous EP, it is still difficult to fully compare these studies because of stimuli diversity, distinct EP components being addressed and, most importantly, the state in which the participants are evaluated, giving the distinctions in cognitive state as targeted by different meditation techniques. Nevertheless, EP analysis is an interesting way of studying cognitive processing on the brain and could provide a broad comprehension of meditation.

### 5.2. Event Related Potentials

ERP are of great interest because of their potential to unravel brain functions related to events, thus enhancing the understanding of cognitive processing of the stimulus by the brain. For example, when a subject attends to a set of visual stimuli, its correspondent ERP response is enhanced (Morgan et al., 1996). This endogenous component influencing ERP is widely explored in meditation studies given that its practice also engages attention. Of particular importance in the discrimination of meditative studies are the evaluation of amplitude and latency of the P300 and N200 components, the first because of its association with attention, sensory-motor information processing and decision making, and the latter is associated with executive control functions.

Complementary information about event related changes are noticeable on spectral features, such as event related desynchronization (ERD), event related synchronization (ERS), event related spectral perturbation (ERSP) and inter trial coherence (ITC). ERD and ERS are respectively described as short lasting attenuation or enhancement of the average amplitude. They are usually calculated on alpha and beta over brain areas related to motor or visual events (Pfurtscheller, 1992; Pfurtscheller et al., 1994; Neuper et al., 2006). In ERSP, event related spectral changes are averaged over time for all EEG (Makeig, 1993). Finally, ITC refers to time-locked phase synchronization on the frequency domain (Tallon-Baudry et al., 1996), as further detailed in section 6.

#### 5.2.1. Auditory Stimuli

One paradigm type that elicits this response is the *oddball*, in which the participant is asked to find a specific (rare) stimulus among others (standard stimuli). In auditory oddball, Sarang and Telles (2006) demonstrated that practitioners of cyclic meditation (CDM-FA) enhanced their P300 amplitude and reduced its latency. Telles et al. (2019) applied this task before and after intervention sessions, also reporting increased P300 amplitudes after focusing (CDM-FA) and defocusing (CDM-OM) meditations, while noticing a decrease after random thinking. The latency reduction was only noticed after defocused meditation. Furthermore, Atchley et al. (2016) reported increased P300 amplitudes for target tones in mindfulness meditators (CDM-FA) when compared to controls. Interestingly, the authors further repeated the experiment, instructing the practitioners to ignore the stimuli and focus solely on their breath; this led to decreased P300 amplitude in meditators, partially in agreement with Telles et al. (2019). Isbel et al. (2019) reported not only reduced latency in P300, but also in N200 in mindfulness meditators (CDM-FA) in comparison to controls, as well as increased N200 amplitudes. Delgado-Pastor et al. (2013) also reported enhanced P300 amplitudes in Vipassana meditators (CDM-OM), and so did Yoshida et al. (2020) in naive participants after training in CDM-FA when compared to an active control group that practiced relaxation. This is suggestive of a higher attentional engagement due to practice. In contrast, Payne et al. (2020) reported no difference in P300 or P200 between experienced mindfulness meditators and controls, suggesting that the differences are not related to meditation practice. Conversely, Cahn and Polich (2008) reported that the P300 amplitude was reduced during Vipassana in comparison to the control group, a finding interpreted as indicative that meditation participants were less reactive to distraction noises. In a further study (Cahn and Polich, 2013), the group continued exploring the ERP epoch in auditory oddball, but instead of the traditional amplitude/latency analysis, they performed an spectral power assessment (Wavelets transform). The increased spectral amplitude was marked around the 300ms after the stimuli, but they also report decreased evoked delta power and reduction of late (500–900 ms) alpha activity in response to distracting stimuli, that was interpreted as indicative of a different dynamics of attentional engagement.

Meditation studies have also explored other waveforms time-locked to stimuli, that emerge with the presentation of a distinctive stimulus amidst a sequence of regular ones. We emphasize the so-called Mismatch Negativity (MMN), a subcomponent of the N200, which relates to automatic detection of environmental alterations. Srinivasan and Baijal (2007) reported a larger MMN amplitude in Sudarshan Kriya Yoga practitioners (CDM-FA) than controls, and this result was corroborated by Biedermann et al. (2016) in Zen, Chan, and Tibetan Mahayana practitioners, indicating that attentional level is superior in practitioners. In addition to that, Fucci et al. (2018) evaluated the MMN in frontal electrodes during oddball, and reported amplitude differences in experts during FA meditation, but not during open presence (NDM). However, for the latter, the authors observed increased latency of late potentials (280–400 ms) in comparison to the former group. Thus, the results indicate dissociation between meditation types, such that, in open presence, experts have enhanced sensory monitoring and reduced perceptual inference, in comparison to FA.

Biedermann et al. (2016) also reported decrease in N100 with auditory stimuli (after the first meditation session), but the results of Barnes et al. (2018) indicate that it may not be an effect of the practice. Miyashiro et al. (2019) also observed modulation in N100 after meditation and the distinction was more accentuated after Mindfulness training (CDM). However, in this case the task was slightly different, and participants had epochs of vocalization compared with listening to their own voice. The modulations pointed to increased N100 amplitude during vocalization, which the authors interpreted as an effect of attention control persisting after the practice.

#### 5.2.2. Visual Stimuli

The analyses of meditation effects on ERP through visual stimuli clearly explore a diversity of psychological tasks, as extensively described in this section. We remark that the usage of the oddball and other paradigms exploring contrasting stimuli still persist. For example, Kakumanu et al. (2019) evaluated Vipassana (CDM) practitioners of distinct expertise levels in a gamified visual oddball. They reported no amplitude differences in the P300 component between groups, although all of them presented modulations in rare stimuli. However, they noticed that experienced practitioners had ERSP increases in alpha and low-gamma, as well as reduction in alpha and theta synchrony and in alpha-theta ITC, and that these differences were more prominent in the most experienced group, with moderate effects in the intermediate group. Thus, the authors emphasize that ERP dynamics are potential correlates to characterize meditation state and trait effects on the brain.

Another similar paradigm is the so-called Go/NoGo, that resembles the oddball but with a modified probability distribution among contrasting stimuli, changing from rare to frequent. In an early study, Banquet and Lesévre (1980) applied a visual Go/NoGo to yogic meditation practitioners, contrasting them with a control group, and reported increased P300 latency. Further studies focused on amplitude differences: Andreu et al. (2019) reported no P300 and N200 changes between Vipassana (CDM) meditators and controls, while Saunders et al. (2016) evaluated an emotion-focused and a thought-focused practice, reporting no P300 changes in either group, but increased N100 amplitude. Van der Lubbe et al. (2018) heightened the stimulation, by adding electrical stimulation to visual stimuli, and reported that although MBSR (CDM-FA) practitioners had modulations in several ERP components, they could not correlate it to the meditation training. The Continuous Performance Task (CPT) is also classifiable as Go/NoGo paradigm, where the participant must sustain attention to repetitive stimuli for a length of time, in order to selectively respond to target stimuli. Zanesco et al. (2019) evaluated two groups of CDM practitioners in this task; the first group enrolled in a meditation retreat, while the second underwent the same program after 3 months. The authors calibrated contrasting stimuli on a participant-based level, in order to match task difficulty between participants and over time. On the first retreat group, the calibrations occurred in all evaluations (prior to, during and after retreat), which resulted in no changes in ERP. As for the other group, calibrations were only performed prior to the retreat, which resulted in changes on N1 (with a smaller latency and greater amplitude) and on P300 amplitude, which suggest enhanced attentional processing after intense training.

The Attentional Network Test also evaluates responses to congruent/incongruent target stimuli, but encompasses preceding cue presentations. Jo et al. (2016) described that mindfulness meditators performed better at this task than controls, and also exhibited higher parietal P300 amplitude. By contrast, Moore et al. (2012) analyzed the Stroop test, which requires inhibitory control of the participant in response to mismatched stimuli, and reported decreased P300 amplitude for incongruent stimuli in mindfulness practitioners (CDM-FA). Meditators also had increased lateral posterior N200 amplitudes over both hemispheres, regardless of stimulus congruence. Furthermore, Van Leeuwen et al. (2012) used a version of the Navon paradigm, which tests the recognition of local/global image features, by assembling compounded stimuli with features that could be mutually consonant or not. The paradigm was evaluated in Buddhist meditators (CDM-OM/FA) and those with global focus had higher P300 than those with local focus. The result is suggestive that the depth of visual processing is enhanced in meditators.

A different approach is to assess sustained attention through ERP analyses. One example applied to meditation is the Psychomotor vigilance task, in which the participant must attend to the screen and respond as fast as possible to visual stimuli. Wong et al. (2018) reported that after a mindfulness (CDM) intervention there was a slight decrease in P300 amplitude followed by ERD in alpha power. Similarly, in the Visuo-spatial task switch, a response is required regarding the position of a visual figure. Hawkes et al. (2014) reported that Tai-Chi and other exercise practitioners had larger P300 amplitudes in comparison to sedentary controls.

In addition, there are other studies exploring the response to affective stimuli: Reva et al. (2014) reported that Sahaja Yoga practitioners (CDM) had attenuated P300 for both positive and negative pictures in comparison to controls, indicating an enhanced emotional controls in meditators. Moreover, they reported no differences in long latency responses (LPP) (400–800 ms), corroborating with Sobolewski et al. (2011) who studied Buddhist practices (CDM-OM), and reported LPP differences only in the frontal areas of the control group; and contrasting with Cosme and Wiens (2015) and Zhang et al. (2019) who reported LPP differences between positive and negative pictures. Katyal et al. (2020) explored a slightly different paradigm, the self-referential encoding task, which traditionally displays words related/unrelated to self, but here it was modified to employ self-related affective adjectives. They also reported LPP differences in the control group as opposed to Ananda Marga (NDM) meditators. Another paradigm in this direction is the Approach Avoidance Task, in which the participant ranks items according to stimulus cues to attractive and neutral images. Baquedano et al. (2019) employed this task to compare food craving in two scenarios: (i) when sensory perceptions were enhanced (immersion); and (ii) after mindful instructions, when the impulses toward food were expected to decrease. The authors reported increased P300 amplitude after the mindful instructions and lower LPP amplitude in the meditative group, which suggests a lower emotional engagement in food craving. More recently, meditation was used to study the performance of ERP brain machine interfaces (BMI), that strongly rely on the subject's attention, such as motor imagery and P300-based approaches (Lakey et al., 2011; Tan et al., 2014). The studies reported enhanced classification accuracy in the mindfulness (CDM-FA) group. Interestingly, Tan et al. (2014) also compared the meditation group with an active control group learning to play a musical instrument, which is also considered a form of mental training. Eskandari and Erfanian (2008) also reported accuracy improvements in TM (NDM) meditators in BMI performance, but via evaluation of ERSP. Thus, these studies indicate that regardless of the type of practice, attention exercises may lead to higher efficiency and more reliable BMI control.

Also, the relation between meditation and learning was further explored by the Serial Reaction Time Task, where the stimuli presentation follows some pre-defined pattern. Chan et al. (2020) evaluated the response to pseudo-random numerical blocks, which contained conditioned sequences. The participants that enrolled in more sessions of Yoga Nidra (CDM-CDM) meditation had increased N200 amplitudes than controls and participants who had only performed the practice once. Savostyanov et al. (2019) performed the same paradigm, but with a distinct experimental design. Here the response of shamata (CDM-FA) meditators was evaluated to neutral and affective sentences of which some contained grammatical errors. They noticed higher P300 amplitudes for long-term practitioners in comparison to those of intermediate experience and novices.

Furthermore, we mention the so-called attentional blink task. It explores a phenomenon happening when two targets (T1 and T2) are presented within a short time interval, and the subject is not aware of the second stimulus. Slagter et al. (2007, 2009) reported that participants bettered their performance in the attentional blink task after a period of 3 months Vipassana (CDM-OM) retreat. This was associated to decrease in P300 amplitude, due to T1 leaving more free mental load for the detection of T2. Another characteristic waveform that has been analyzed through visual stimulus is the Contingent Negative Variation (CNV), a characteristic slow negative component observable when two stimuli are sequentially presented to the subject, and the first serves as an indication to the second. The CNV revealed differences between TM (NDM) and control groups: Travis et al. (2000) found higher CNV amplitudes on frontal and central midline. Also, meditators were less influenced by a distractor, introduced before the second stimulus. These findings were confirmed by further studies of the same group (Travis et al., 2002, 2009, 2018), in which they continued the evaluation of the CNV potential in more experienced meditators. The repetition of the task in non-meditators did not affect the CNV amplitudes (Pauletti et al., 2014), which suggests that this modulation might be a characteristic consequence of the practice. Lasaponara et al. (2019) further evaluated two variations of the task before and after a session of the Quadrato Motor Training. In the simpler task variation, the participant is only entitled to respond to the second stimulus, while in the more cognitively complex one, the subject is required to make a decision in the presentation of the second stimulus. Their result points to increased CNV amplitude in the simpler task, in agreement with Travis et al. (2000), but presented decreased amplitude in the more complex one, which is explained as evidence of attentional facilitation promoted by meditative practices.

Interestingly, Kornmeier et al. (2017, 2019) adapted a visual task with ambiguous images, i.e., although the stimuli remained constant; the participant's perception might be altered. They presented the Necker cube discontinuously, and evaluated the ERP response during perceptual reversal in two scenarios: (i) when participants simply observed and indicated an inversion; and (ii) when they performed the same task, but were instructed to try to sustain their perception. A group of CDM-OM meditators (from several traditions) was compared with controls, and the authors reported increase in the latency of a reversal related ERP, the centro-parietal event-related positivity, which occurs after ∽500 ms, suggesting that meditators have volitional control over the duration of percepts.

Finally, we recall that some studies report the effect of meditation practices in cortical waveforms known to precede an specific voluntary action, named readiness potential (RP), and that might be indicative of preconscious brain activity modulation. Jo et al. (2014) studied the voluntary movement link with sensory feedback in Mindfulness meditators (CDM-FA), and reported that lower perception delays (binding effect) were associated with negative deflections of slow cortical potentials, an ERP known to precede voluntary movements. In further studies of the group, the results suggest that mindfulness meditators also had a more reliable control over their slow cortical potentials (Jo et al., 2015). In a similar way, Radin et al. (2011) presented randomly visual and auditory stimuli, and described a distinction in the pre-stimulus waveforms when visual and auditory ERP were compared only in non-dual meditators (NDM).

Even though there are differences on stimuli nature, on the cognitive tests, as well in meditation categories and expertise levels and also in the number of repetitions, we observe that ERP studies suggest that, in general, meditation leads to modulation of several components, with emphasis on P300. Thus, ERP analysis is an interesting way of studying the cognitive processing and may provide a broad comprehension of the effects of meditation on the brain. Nevertheless, it is important to consider methodological limitations in several studies (Barnes et al., 2018), such as: (i) the lack of a control group; (ii) the lack of a control condition; and (iii) the fact that ERP are usually not measured during meditation sessions, which hinders the direct attribution of the modulations as a consequence of the meditative state, given that they can be trait effects or compensating attentional states. The incorporation of new methodological enhancements to ERP analysis involves the consideration of the entire scalp topography at a given time stamp instead of a single electrode, likewise the one performed by Bailey et al. (2019), Zanesco et al. (2019), and Payne et al. (2020), which has advantages, such as being reference-free (Michel et al., 2017).

## 6. Synchrony

There are experimental evidence that rhythmic oscillations have a link with cognitive phenomena and information processing in the brain (Buzsáki, 2006). Another important issue in rhythmical waves' characterization is their topological distribution on the head, i.e., how to quantify the relation between the neural activity of two distinct sites. Oscillatory signals are characterized by three distinct features: *frequency, amplitude* and *phase*. Frequency is associated with the waveforms rate of change; the amplitude, to the waveforms magnitude and energy; and the phase, with the correspondence between time and the waveform cycle. Therefore, given the features of oscillatory signals, there are several possible rhythmical interaction possibilities, as reviewed by Hyafil et al. (2015): phase-frequency coupling, phase-phase coupling, phase-amplitude coupling and amplitude-amplitude coupling. The power spectral analysis, performed in the great majority of meditation studies, cannot quantify the relation between frequencies, ignores the phase information and relies on the assumption that the signal results from a linear process, which conflicts with the non-linear dynamic of the electrical brain activity. Thus, phase-coupling phenomena, manifesting as interaction between components, are also dismissed and some attempts tried to quantify these types of interactions (Sigl and Chamoun, 1994).

The coherence, is one measure that quantifies phase-phase relationships between two signals. Coherence values range from zero, when the signals are unrelated, to one, when they are perfectly correlated or linearly related. Therefore, coherence can be understood as cross-correlation in the frequency domain. The spectral content of EEG signals, in particular, is characterized by uneven energy distribution within frequencies: lower frequencies have higher amplitudes, and thus more energy, which decays exponentially as the frequency increases. Therefore, by computing a simple correlation between gamma and delta bands, for example, one achieves a result that is substantially dominated by delta activity, i.e., the signal of higher energy. Thus, coherence computation represents evolution in signal processing, because it is robust to unequal energy distributions and allows the observation and/or interpretation of the phenomena in finer bandwidth granularity. Higher coherence was associated with potential information transfer (Dillbeck and Vesely, 1986), functional coupling and functional coordination (Murata et al., 2004).

Regarding meditation studies, Banquet (1973) reported visual observations on the distribution of rhythmical alpha activity spreading from occipito-parietal to anterior areas and on theta and beta waves, which appear in the frontal channels to subsequently diffusing to posterior areas. Transient asymmetry between right and left hemispheres was also observed appearing at the onset of meditation, and developing from slow to fast frequencies with the beta-dominant activity starting in the frontal left hemisphere and moving to the occipital channels. Such results motivated the application of coherence to characterize meditation correlates. The first studies, despite lacking homogeneous patterns among participants, reported coherence increase in frontal areas within the higher alpha band observed after the beginning of TM meditation (Levine et al., 1977; Dillbeck and Bronson, 1981). Levine et al. (1977) further reported coherence increase in central areas observing that coherence peaks during meditation generally spreading to lower frequencies, in the theta band, and that sometimes coherence would abruptly decrease after meditation end.

In addition coherence was further employed to analyze experimentally many meditation paradigms. Travis and Arenander (2006) reported asymmetric coherence findings in frontal areas when meditation (TM-NDM) was compared with either eyes-closed resting or with cognitive computation. In a complementary way, Travis et al. (2017) reported theta-2 plus alpha-1 frontal, parietal, and frontal-parietal coherence increases when participants listened to Vedic mantra recitation, in comparison to TM practice. Farrow and Hebert (1982) further evaluated breath suspension epochs in TM meditation, pointing to significant changes in mean coherence values in alpha, beta and theta bandwidths before and during meditation that were interpreted as increase in the long range orderliness of brain activity. Similarly, Badawi et al. (1984) also found significant differences in mean coherence; but they analyzed simultaneously all frequency bands; the study compared TM meditators with a control voluntarily breath-holding group. Moreover, Badawi et al. (1984) stated that neither their study nor Farrow and Hebert (1982) verified differences in the spectral power between meditation and breath holding groups. These findings would indicate that coherence could be more suited tool in characterizing neural correlates.

Additionally, bilateral frontal alpha and theta coherence were positively correlated with subject performance at a learning task, being increased in practitioners who learned an advanced TM technique between the first and second evaluations when compared with a group lacking those instructions (Dillbeck et al., 1981). Dillbeck and Vesely (1986) further explored the paradigm, conducting a controlled study with groups of meditators and non-meditators, reporting that meditators presented higher frontal alpha coherence. Besides, recordings of control subjects had more muscle artifacts. Travis and Wallace (1999) reported no significant differences in alpha power between eyes-closed resting and TM meditation. Nevertheless, the anterior-posterior and the frontal regions showed significant differences in higher alpha coherence. Travis et al. (2002) also reported significant differences when evaluating coherence in frontal areas of TM (NDM) practitioners. Travis (2001) working with TM and Qin et al. (2009) with Qigong (CDM-FA) complemented the findings in frontal and parietal areas, and Baijal and Srinivasan (2010) also noticed increased frontal theta coherence in CDM-FA meditators.

Regarding studies with less experienced practitioners, Murata et al. (2004) also reported significant differences in frontal areas alpha coherence in novice Zen (CDM-FA) practitioners. Complementing these findings, Gaylord et al. (1989) reported differences not only in frontal alpha coherence but also in theta during the meditation of a group that had a course on TM. However, after 1 year, in the follow up, no more longitudinal changes were observed, which might be due to the participants' lack of regular practice during that period. Travis et al. (2010) complemented with findings for alpha coherence in frontal and parietal areas as well as with inter-hemispheric differences, and Aftanas and Golocheikine (2001) confirmed increased frontal theta coherence in CDM-OM meditators. By contrast, Kim et al. (2014) computed averaged coherence over all channel combinations in CDM-FA meditation, and reported significant increases in alpha, beta, and gamma bands compared to the subjects' baseline.

Nevertheless, the coherence calculation embeds technical limitations that must be considered when one is interested in comparing results from different studies. Firstly, it is known that the coherence of EEG signals has low robustness to variations on the reference signal: mean activity or ear electrodes result in striking differences in connectivity patterns (good examples are provided by Lehmann et al., 2006), which hinders further comparisons among studies (Fein et al., 1988). The recommendation stresses the use of reference-free setups, like the double banana. The mean average reference is only advised when high-density setups (more than 128 electrodes) are available. Another possibility is to compute the coherence between the EEG's second-derivative, which is a reference-free signal. Still, not all distortions are corrected, since long-range coherences remain underestimated (Schiff, 2005). Moreover, caution is required regarding the number of points in the dataset, given that spurious coherence may arise and corrective models/bootstrapping techniques, such as the ones reviewed by Schiff (2005), are rarely used in meditation studies. Therefore, one should carefully analyze if the EEG references agree when comparing distinct studies on meditation. On top of that, we recall that the traditional coherence, calculated with the FFT, is unsuited to characterize non-stationary signals and, that there is growing evidence that distinct cortical areas interact through non-linear features and, in addition, that brain signals interdependencies may change rapidly (Stam and Van Dijk, 2002; Fingelkurts and Fingelkurts, 2008). Thus, the aforementioned considerations motivate and require the development of new processing techniques and further enhancements to coherence processing pipelines in order to characterize neuronal correlates. Furthermore, in coherence the effects of amplitude and phase interactions are not completely separated (Lachaux et al., 1999). This scenario was evaluated in a controlled simulation: limitations were found as deviations from expected values, the lack of ability to characterize intermittent synchronizations and its poor performance within increasing signal-to-noise ratios (Lowet et al., 2016). EEG signals are known for their low signal-to-noise ratio, which also motivates the use of new measurements to quantify phase-phase relationships.

One example in this sense is the Bispectrum estimation which basically corresponds to the analysis of the second order moment of the spectra, and measures the coupled energy between frequencies (quadratic phase coupling) (Sigl and Chamoun, 1994; Bullock et al., 1997). The method can also be expanded to compute higher order interactions. This approach was applied by Goshvarpour et al. (2012) to characterize CDM-FA meditation, and indicated increased magnitudes during meditation compared to baseline, incremented phase coupling in the occipital region (centered in the Pz electrode) and a shift in the harmonics to higher frequencies.

Another example is the Phase-Locking Value (PLV) (Lachaux et al., 1999). Basically, the PLV computes the phase difference between two signals and then its mean over several epochs. PLV reaches one, its peak, when the phase difference is zero, i.e., when the signals are phase-synchronized. We recall that some adaptations have already been proposed to contemplate constant phase-differences other than zero, which would relate biologically to signals with a constant delay (Martins et al., 2016). Moreover, in opposition to coherence, the PLV does not assume signal stationarity, and accounts for better phase-synchronization information flow among networks.

In meditation studies, Slagter et al. (2009) compared the ERP response of two participant groups to an attentional-blink task. Both groups were evaluated twice, in 3-months period, the first before and the second after a Vipassana retreat (CDM-OM). The control group would meditate daily for shorter time. Phase locking was observable within the theta band in the right frontal and in central scalp sites when subjects perceived the stimulus, and was absent either when the stimulus was unnoticed or absent. Therefore, theta phase-locking was regarded as a neural correlate for target perception. Furthermore, mental training has a clear relation with theta-phase locking increase and ERP amplitude decrease: when these features are plotted (Figure 4 in Slagter et al., 2009), two clusters are distinguishable, one for practitioners and one for novices. The decreased ERP amplitude was associated with reduced brain resources engaged in processing the stimulus; therefore, resources for further information processing are quickly available. Lutz et al. (2009) further analyzed the same subjects, but engaged in a distinct activity, the dichotic listening task. They have also employed PLV analysis and corroborated the findings on phase consistency increase in the theta oscillatory band but only in response to the target stimulus. Theta phase consistency was correlated with less variability in the response of the participant, which the authors related to increased cognitive control capacity and a possible noise reduction in attentional networks. Furthermore, meditators presented intensified ERP phase consistency in response to any deviant tone in a broad frequency band (1–30 Hz), but, in contrast with the findings of Slagter et al. (2009), it was not accompanied by changes in ERP amplitude. Nevertheless, amplitude differences were noticeable in ERD on the beta band, which was also interpreted as evidence that meditators reduced their cognitive effort. Similarly, Jo et al. (2017) evaluated mindfulness meditators (CDM-FA/OM) and controls ERP in a flanker type paradigm, also reporting an enhanced synchrony in theta oscillations: the PLV was higher in incongruent trials, but the connectivity was enhanced in the medial pre-frontal cortex (PFC) and motor cortex. Incongruent trials were also related to increased theta power, higher in meditators than in controls.

Berkovich-Ohana et al. (2013), used the PLV (under the name of mean phase coherence) to characterize the functional connectivity of the Default Mode Network—a brain-functioning network of spontaneous cognition that is active when we are not involved in any cognitive task– (DMN). The authors analyzed CDM meditators engaged in a time-production task vs. resting state. The EEG cap had 65 electrodes, which were collapsed in nine regions of interest, unlike usual approaches which consider channel-channel relationships. Then, the mean PLV was used as a network parameter to compare effects in distinct hemispheres within the theta, alpha and gamma bands. The authors reported hemisphere asymmetries in the alpha band, such as higher PLV in the right hand side and a PLV increase in alpha during meditation. They further reported PLV differences when comparing the resting state of meditators and control subjects. Furthermore, Yoshida et al. (2020) used a modification of the PLV (under another name) to assess synchrony: the single-trial PLV (Lachaux et al., 2000) is calculated across successive time-steps instead of being calculated as averaged of multiple trials. The participants were divided and a subgroup enrolled in a CDM-FA protocol, while the other received training in a relaxation protocol. Both groups were evaluated during an auditory oddball. The authors established a correlation between the synchrony of some pairs of electrodes and P300 amplitudes of meditators that was not existent in controls.

Lehmann et al. (2006) assembled functional networks considering many possible references for coherence calculation in comparison with PLV, showing that PLV and coherence networks resembled one another when the time series of model sources were considered. On the other hand, coherence networks were strikingly different depending on the chosen reference. The authors compared functional connectivity between resting and CDM-FA meditation, employing many epochs of an experienced subject's dataset. They reported decreased left-right intracerebral coherence in delta band, and augmented anterior-posterior intracerebral coherence in theta band. However, in this study, the dataset of only one subject was considered. Therefore, the authors conducted further analyses, comparing functional connectivity in all bandwidths in meditators of five different traditions (Lehmann et al., 2012). Notwithstanding, the method was improved by the attenuation of volume-conduction artifacts, through the removal of zero phase angle coherences, and is named lagged coherence. They reported a global coherence decrease in all five groups, which was interpreted as higher functional independence among areas; as well as similar functional topographies, with low variance across groups. Interestingly, the result may be interpreted as evidencing major commonality on neural correlates among distinct meditation traditions. Higher independence was hypothesized to explain subjective experiences, such as: non-involvement; detachment and letting go; diminished information handling; expansion of consciousness experiences; dissolution of ego borders and all-oneness. On the other hand, Kim et al. (2013) employed the same methodology on CDM-FA practitioners after 8 weeks of training and reported decrease in mean coherence averaged from all channels in the alpha band.

It is worth mentioning additional improvements that have arisen on coherence analysis as some have been translated to meditation studies: Dissanayaka et al. (2014, 2015) compared the functional connectivity during wakefulness, drowsiness and meditation using (i) the traditional coherence operator; (ii) another coherence estimator that enabled better spectral resolution (minimum variance distortionless response-MVDR); and (iii) with a directional measure of information flow (Direct Transfer Function-DTF). Shaw and Routray (2018) also employed DTF to characterize Kriya yoga meditation (CDM), in addition to the partial directed coherence (PDC). These studies use directional measures, which potentially allow further conclusions regarding brain connectivity. Nevertheless, we recall that its broad application requires caution, since the measure hypothesizes a linear and autoregressive model, which might disregard non-linear interactions. Moreover, Kopal et al. (2014) calculated coherence using the wavelet transformation instead of the traditional FFT. Furthermore, the group performed separate analyses on the real and imaginary part of the coherence operator, thus isolating volume conduction effects in the real part, and phase synchronization in the imaginary part. The groups were small (seven Vipassana meditators—CDM—and seven controls) and the results so far exploratory, but one could observe emergent connections among the frontal regions associated with volume conduction, and also in the frontal occipital associated with synchronization.

The usage of the coherence imaginary part is seen in other meditation studies, due to its robustness to spurious interactions related to volume conduction (Hauswald et al., 2015; Lee et al., 2018a; Jiang et al., 2020). Similarly, Stam et al. (2007) proposed to disregard zero-lagged/opposite-phase interactions to avoid volume conduction interferences at the expense of also ignoring actual instantaneous interactions. Their index, the Phase Lag Index (PLI) was used by van Lutterveld et al. (2017) to evaluate functional interactions in novice and experienced meditators. All these studies are further discussed in section 9. It is important to notice that while these approaches reduce type I errors by disregarding volume conduction effects, they also increase type II error susceptibility, missing actual connections that emerge due to small phase lags or frequency non-stationarities (Cohen, 2015).

Although the studies seem to indicate increased synchrony in meditators, we remark that neither PLV nor (traditional) coherence provide information regarding the directionality of the interaction. Even though the computation of phase-phase relationships by PLV proves useful when correcting some distortions inherent of traditional coherence computation, the reduction of natural phenomena exploration solely to phase relations may be an oversimplification. Most likely one should consider *simultaneously* the modulations of phase and amplitude. We remark the inexistence nowadays of a technique that can best compute the complexity of neuronal phenomena, and likewise, this also applies to meditation studies. Therefore, the experimenter must be fully aware of which signal effects are expected/of interest in characterizing the meditation experience/the meditator's response in the myriad of experimental paradigms already covered in previous works.

For example, Fingelkurts et al. (2007) and Fingelkurts and Fingelkurts (2008) stated that neuronal assemblies activity is translated into non-linearities appearing in EEG signals. These non-linearities can be characterized through abrupt amplitude changes in EEG signals. The amount of rapid amplitude variation events corrected by the number of chance-expected events was quantified in the so-called *operational synchrony index*. Then, through the detection of “quasi-simultaneous” non-linearities in signals within distinct sites, one could detect regions with related activity. Thus, sets of time-coupled EEG channels were called *operational modules* (OMs) and characterize a broader range association among neuronal activities; this cannot be gathered by phase-synchronization metrics. Under this perspective, Fingelkurts and Fingelkurts (2011) reported evidence suggesting that DMN consists of three distinct OMs with interleaved activity: two symmetrical occipito-parieto-temporal and one frontal. In addition, the functional connectivity within these modules was related to distinct arousal levels of the patients: it was higher on fully conscious healthy patients, decreasing in the minimally conscious patients and was even lower on the ones in vegetative state. Therefore, Fingelkurts et al. (2016) investigated functional connectivity changes between the alpha band of the anterior and posterior modules as a function of meditation training. They reported a significant decrease in the overall DMN, in agreement with previous functional magnetic resonance imaging (fMRI) findings. the authors proceeded to extend the analyses to separated OMs and found that operational synchrony increased in the frontal module and decreased in the left and right posterior ones. The results indicate that the effects of meditation in DMN dynamics is complex, but are suitable for characterization with EEG. Furthermore, it is suggested that the information distribution within OMs is uneven, indicating that OMs may be related to distinct aspects of self-referential thought.

## 7. Source Analysis

The determination of which brain regions modulate their activity in association with cognitive/behavioral processes is of main importance in neuroscientific studies. Due to major advances in the signal processing field, the EEG signal has been increasingly used as a neuroimaging tool, providing space-time information on brain function (Michel and Murray, 2012). In this context, mathematical models have been created to deduce, from the voltage potentials measured with EEG (Biasiucci et al., 2019), the spatial distribution of current sources in the brain. More specifically, current source time-series are reference-free and already account for the similar electric patterns captured by neighboring EEG channels. Source analyses computed from EEG outcomes has poor spatial resolution compared with that achievable with fMRI; nevertheless, the brain dynamics can be characterized with much better temporal resolution.

Currents are mathematically represented by entities called *dipoles*, which quantify the overall polarity within a brain region. The analysis of current source spatial distribution is initiated by the so-called *forward problem*, i.e., finding the voltage distribution resulting from a set of hypothetical dipoles and a volume conduction model of the head. The next step is the so-called *inverse problem*, which receives the voltage output computed in the forward problem and combines it with the actual EEG signal in different scalp sites. As result, the analysis estimates the current sources suited to these measurements (Grech et al., 2008). However, the inverse problem has no unique solution, i.e., several distinct current distributions suit the measured data. Therefore, further assumptions/constraints are added, in order to furnish an unique solution. Currently, many algorithms have already been proposed to perform EEG source analyses and, apart from other mathematical differences, they may adopt distinct assumptions/constraints when solving the inverse problem (Pascual-Marqui, 1999; Grech et al., 2008). Grech et al. (2008) classified these algorithms in two major categories (non-parametric/parametric), and the major difference between them has to do with the prior assumption (or not) of a fixed number of dipoles.

Non-parametric models assume that there are several dipole sources with fixed locations and possibly fixed orientations distributed over the brain/cortical area. The orientation is usually normally aligned and therefore, the algorithms focus on estimating the amplitudes of these dipoles (Grech et al., 2008). They have been largely employed in the meditation field, mostly using the *Low Resolution Brain Electromagnetic Tomography* (LORETA) family of algorithms. The LORETA algorithm approximates a solution to the inverse problem with the *a priori* assumption that adjacent sources tend to have the same magnitude and orientation. Thus, the algorithm selects the smoothest possible solution and the output has coarser spatial resolution. Furthermore, it is assumed that the white matter does not originate current (Pascual-Marqui et al., 1994, 2011). The original algorithm has limitations when trying to localize deeper sources (Grech et al., 2008), which led the authors to new proposals, such as the standardized LORETA (sLORETA) (Pascual-Marqui, 2002), which employs a normalization of current densities that enabled source characterization with unbiased localization error under noisy conditions. Later on, another derivation was proposed: the exact LORETA (eLORETA) (Pascual-Marqui, 2007; Pascual-Marqui et al., 2011), with a null localization error and no standardization. Another non-parametric solution complementary to LORETA that has been used in meditation is the Variable Resolution Electromagnetic Tomography (VARETA), which requires a very fine grid spacing, but incorporates both concentrated and dispersed sources disregarding points of no activity (Valdes-Sosa et al., 2000). Finally, we mention the Minimum Norm Estimates (MNE), which is well-suited for source activity extending over broader cortical surface areas although it favors weak and surface sources (Grech et al., 2008). Complementing, we cite the Multiple Sparse Priors (MSP) algorithm (Friston et al., 2008), which is further improved because it has better discriminative power of multiple active sources, even if there is a correlation between them.

By contrast, parametric models assume the existence of fewer and uncorrelated dipoles, but their number, location and orientation are unknown. Some algorithms are dynamic and consider that dipoles are changeable over time. Among the most used in meditation, are the *beamforming* approaches or spatial filters, that are spatially adaptive filters, i.e., they treat signals coming from the electrodes in a way that only those coming from sources of interest are preserved, while the rest is attenuated. This approach is reiterated to encompass distinct physical locations, and, therefore, does not require prior knowledge of the number of sources and anatomical information (Grech et al., 2008). Beamforming has the advantage of producing more spatially focused results. Some examples in this sense are the Dynamical Imaging of Coherent Sources (DICS), that comprehend the calculation of source strengths for each voxel in the frequency domain (Groß et al., 2001), and the Linear Constraint Maximum Variance algorithm (LCMV), that computes in the time domain.

We recall that the use of source analysis algorithms to characterize meditation also includes studies aimed at comparing the effects of the practice with other activities. For example, Chan et al. (2008) compared novices listening a recorded practice of the Triarchic body-pathway relaxation technique (TBRT, classified as ADM) and segments of classical music. They reported increased relative theta power in anterior and posterior regions of meditators, which was further explored with LORETA, pointing to a source with increased activity for this rhythm in the anterior cingulate cortex (ACC), an area linked with attentional processes. According to the authors, it reinforces the idea that attentional processes, and not cortical arousal, are the main psychological response evoked by the practice. In addition, Thomas et al. (2014) studied intermediate and advanced Satyananda Yoga practitioners (classified as NDM) during meditation, baseline and mental calculations. Intermediate practitioners had greater alpha-1 and theta activity during mental calculations and meditation, whereas the advanced had marked activity at higher frequencies (beta and gamma), which was particularly salient during the practice. These spectral findings were further explored with eLORETA: the comparison between groups in all experimental conditions suggests that meditation leads to increased differences in the right insula, right inferior frontal gyrus and right anterior temporal lobe. Thus, the authors interpreted the results as indicative of a core right-sided network. Given that the coverage of the network related areas greatly expanded during meditation practice to also include homologous regions of the left hemisphere, they state that this network is progressively modulated over the course of the meditation practice. Finally, they were expecting modulation in the DMN resulting from practice length, which was not confirmed by the experiment. Moreover, Travis and Parim (2017) used eLORETA to compare the sources of TM practitioners (NDM) at meditation, baseline condition and during an attentive task. The authors reported similar patterns at TM and resting, and that the comparison of TM with the attentive task indicated activation in the posterior cingulate cortex (PCC) (alpha/theta) and in the precuneus (alpha), which are both parts of the DMN. Moreover, there was deactivation in beta on ACC, ventral lateral and dorsolateral PFC. Therefore, given the activation of DMN parts, and the similarity between TM and resting, the authors argue that TM cannot be classified as an FA practice.

In the meantime, other studies described how the sources of brain activity modify during meditative states. Lehmann et al. (2001) evaluated the gamma bandwidth changes caused by three CDM-FA practices and two NDM in one experienced meditator. This individual did the five types of meditation in sequence (two visualizations, followed by verbalization—mantra, self-dissolution and self-reconstitution). Gamma activity was associated to focused arousal, supporting the hypothesis that this bandwidth is related with states of task-relevant neural circuitries (Kaiser and Lutzenberger, 2005; Jensen et al., 2007). The source gravity-center-location analysis in gamma pointed to different areas for each of the meditations, with the exception of self-dissolution vs. self-reconstruction. LORETA was also performed and they confirmed the activation in different regions for each meditation type: the visualization, the mantra and the self-dissolution/self-reconstruction meditations corresponded to the activation of brain areas related to vision, language and the right PFC, respectively. The study shows that distinct meditation techniques produce activation in different brain areas, promoting characteristic physiological states, even though they may be classified in the same category. Therefore, this work reinforces the difficulties in finding common correlates for different meditation techniques (Nash and Newberg, 2013). This statement agrees with the results of Travis (2011), who did a study using eLORETA to differentiate traditional TM from TM-Siddhi, which is considered a more advanced TM practice, but both were classified as NDM. A group of experienced meditators was randomly assigned into two groups: TM-only group, or TM-Sidhi group. Results showed significantly stronger sources of alpha-1 in the right and left parahippocampal gyri, the lingual gyrus, the right fusiform gyrus, and right inferior and medial temporal cortices for TM-Sidhi practice compared to TM-only group. Finally, Schoenberg et al. (2018) used LORETA to assess the sources of four states of the Essence of Mind Practice (NDM), observing marked amplitude decrease in the magnitude of current density vector on gamma bandwidth in the ACC when subjects entered meditation; this value progressively increased as the meditation states progressed. Therefore, the authors suggest that this is a potential neuronal correlate of a state of non-duality. Moreover, the transition between baseline and practice (first stage) also became evident by decreased amplitudes in alpha beta and gamma in DMN areas, ACC, insula, precuneus, PCC and in the superior and inferior parietal lobule. Interestingly, no activity was observed in frontal areas, dismissing a role of the frontoparietal control network in self-regulation mechanisms in this meditation practice.

Some studies further characterized the meditative state, i.e., the emergent features that characterize immediate effects of meditation in comparison to a baseline. Travis et al. (2010) used eLORETA in TM (NDM), reporting increased alpha-1 activity in the anterior, dorsal and posterior cingulate gyri, as well as in precuneus, left lingual gyrus and in the parahippocampus. The superposition of these areas with the DMN led the authors to suggest that there is a relation between the meditative experience and intrinsic default modes of brain function. Also, Faber et al. (2015) hypothesized that Zazen meditation (CDM-OM) and resting would activate different brain areas: the sLORETA showed that during meditation, alpha-1 and alpha-2 increased activity in a large cluster in the right hemisphere compared to resting, including the PFC, insula, somatosensory and motor cortices and anterior temporal areas. There was decreased activity in these bandwidths in the left angular gyrus. Considering Zazen less than resting, the activity in beta-1 and beta-2 bandwidths decreased during meditation in a bilateral posterior cluster, including the visual and somatosensory association cortices (pre-cuneus), as well as the PCC. These results reinforce the idea that meditation is a cognitive task. In this study, meditation exhibited less DMN activity compared to resting suggesting increased present moment awareness. Similar results were reported by Hauswald et al. (2015), that used DICS to contrast Zen practitioners (CDM-OM) at meditation and in a resting period after the practice, reporting a cluster with reduced activity during practice; this cluster included the somatosensory cortices, the right insula, right middle temporal gyrus, and right inferior, middle and superior frontal gyri. Finally, Jiang et al. (2020) studied the heartbeat-EP of CDM-FA/OM practitioners, using DICS. When the baseline recordings were compared between meditators and a control group, two clusters emerged: one included the PCC and the precuneus bilaterally, and another the medial PFC. In addition, they also evaluated meditative traits (refer to next paragraph), by comparing practitioners at practice and at baseline. A cluster was noticed bilaterally in the ACC, extending across the superior medial frontal gyrus. Given that many of these areas overlap with the DMN, these results suggest that the neuronal representation of visceral activity (heartbeat) in DMN may integrate neural mechanisms of meditation.

As previously exemplified by Jiang et al. (2020), some studies complemented the description characterizing the meditative traits by comparing practitioners at meditation with a control group. Lavallee et al. (2011) used sLORETA to study a group of concentrative meditators (CDM-FA) matched to an active control group (Bensons' relaxation response), meditation-naïve. Both groups showed decreased activity in the right inferior parietal lobule, in multiple frequencies compared to baseline. Moreover, compared to baseline, meditation increased beta-1 activity in the right ACC and beta-3 in the left precuneus (DMN areas), while decreasing gamma activity in the right inferior parietal lobule. On the other hand, when doing relaxation for the first time, the control group presented decreased beta-2 activity in the right parahippocampal gyrus of the limbic region, and decreased delta activity in the right inferior parietal lobe. The comparison between groups pointed to greater beta and gamma power in the right frontal regions of meditators, including the middle, superior, and inferior gyri. While the results point to areas associated with decreased external awareness and increased self-referential processing in meditators, in controls they indicate decreased activity in areas related to environmental information processing. Finally, Tei et al. (2009) used LORETA to compare sources between Qigong (CDM-FA/OM) experienced meditators and a control group during resting. In this study, only the delta bandwidth indicated a difference between groups, being stronger in meditators' PFC and ACC regions and weaker in motor, in visual and somatosensory association cortices, and also in the following areas: the left temporo-parietal junction, left precuneus, bilateral fusiform and right parahippocampal gyri. Moreover, the authors stated that the neuroelectric trait of these meditators was related with their practice time: Compared to controls, the practitioners presented similar brain activity at meditation and at rest, i.e., an inhibition of appraisal systems.

Another strategy to evaluate trait effects of meditation was to compare the meditators response/performance to/in a series of stimuli/activities and in the sources of ERP components. Moore et al. (2012) performed VARETA in ERP signals, comparing mindfulness meditators (CDM-FA) and controls in the Stroop task. They related decreased P300 amplitude in meditators to decreased activities in the lateral occipitotemporal and inferior temporal regions of the right hemisphere. Moreover, the increased N200 amplitudes in meditators for congruent stimuli corresponded to increased activity in the left medial occipitotemporal areas, in contrast with decrease in similar areas observed in controls. In an additional study, Malinowski et al. (2017) also used VARETA and the Stroop task to compare the responses of elderly volunteers to training in mindfulness and in mental arithmetic calculations; the authors corroborated previous findings of increased N200 amplitude in meditators and its association with the dorsal attention network. Nevertheless, they reported no changes in P300 between groups. Van Leeuwen et al. (2012) also evaluated P300 and N200 CDM-FA/OM meditators and controls, but in a version of the Navon paradigm and using MSP. They also report, in meditators, activation in the inferior temporal gyrus, inferior occipital/fusiform gyrus and dorso lateral PFC, which correspond to higher order processing areas and corroborate the better attentional allocation in this group as compared to controls. Finally, Kornmeier et al. (2019) also employed MSP in CDM-OM practitioners and controls to assess the differential responses to ambiguous stimuli. They reported an initial result in the Frontal Negativity—a differential ERP prominent after 160ms after stimulatory onset—when perception was reversed, corresponding to increased activities in the middle/posterior cingulate cortex, left supplementary motor area and right medial PFC.

Although many studies point to activities in the ACC, pre-cuneus, PFC, PCC, and parahippocampal gyri, there are inconsistencies in the aforementioned studies, which might be attributed to diverse meditation practices, varying levels of meditators' expertise and different acquisition protocols. The lack of standardization in dividing alpha and beta bandwidths, for example, brings additional difficulties to compare their findings. In addition, case studies (e.g., Lehmann et al., 2001) with few experienced meditators do not allow generalizations concerning the brain regions involved in different meditation practices because cognitive strategies and brain mechanisms may vary from one experienced individual to another. Comparing groups of experienced meditators who are familiar with different practices may bring a better understanding of the underlying neurobiology aspects (Travis, 2011). Moreover, it is important to recall that some technical parameters may hugely impact the outcomes (Brodbeck et al., 2011): electric source imaging can reach sensitivity and specificity values resembling the ones from MRI, PET, and SPECT if the recordings are performed with a high density cap (128–256 channels) and with an individual structural image as head model. However, the analytic potential drops substantially with smaller number of channels (<32 channels) and a template for head modeling.

Finally, we mention studies which combine source analysis to explore synchrony interactions. In these, the synchrony algorithms are no longer applied to the channel's signal, but to the resultant source time-series. For example, Lehmann et al. (2012) and Milz et al. (2014) applied lagged coherence to evaluate the functional connectivity between meditation and resting. The former analyzed practitioners from a variety of traditional NDM practices, and reported that lagged coherence was lower at meditation than at rest. These findings were partially confirmed by the latter authors, who tested CDM-FA practitioners and reported lower functional connectivity between cognitive control and sensory perception areas during meditation.

## 8. Non-linear Techniques

Furthermore, aiming at describing meditation physiology and possibly unveiling other neural correlates, new processing techniques based on non-linear signal dynamics, or *chaos*, and grounded on spectral findings have been incorporated to the field. Roughly, chaotic systems are characterized by a set of deterministic equations, but nonetheless with a dynamic largely susceptible to the initial conditions. One major feature of chaotic systems is the emergent recurrences, i.e., in a system regulated by a large number of related variables but which are impossible to measure (such as occurs in the brain), the chaos theory suggests that the observations of a single variable dynamics enables inferences about the dynamic structure of the entire system. The requisite is that the observations have enough accuracy and are registered for a sufficient length of time. For example, the membrane potential of individual neurons, under a set of circumstances, may resemble chaotic oscillations (Elbert et al., 1994). One property used in characterizing chaotic systems relates do the *dimension estimation*, which roughly calculates the number of variables required to describe the system's dynamic. Therefore, the higher the dimension of a system, the higher its *complexity* (Pritchard and Duke, 1995).

Regarding meditation studies, Aftanas and Golocheikine (2002) analyzed the datasets of experienced Sahaja Yoga practitioners (CDM-OM) at meditation and at rest. Initially, 62 EEG channels were gathered as 12 clusters on the head. Next, they established a significant correlation between the spectral signature (estimated with FFT) and the complexity analysis (by means of the so-called *dimensional complexity* index—DCx) in theta, alpha (negative correlations) and beta (positive correlation) bandwidths over the midline anterior cortical regions. Its inverse correlation with the spectral power in the low frequencies led the authors to hypothesize that a meditation feature would be its lower complexity because practitioners engage in less distraction during the practice. Gao et al. (2016), Sik et al. (2017) also reported decrease in complexity related to CDM meditation. They combined both the Wavelets Transform with the complexity and source analysis, to analyze the MBSR program. More specifically, the Wavelets Transform was used to compute the so-called signal *Wavelet Entropy*. Generally speaking, the higher the signal entropy, the higher the average information amount of the signal. The EEG Wavelet Entropy decreased during MBSR compared to the resting state; however, there was no significant difference when comparing early (2 weeks) and late stages of the program (8 weeks). Frontal and parietal-occipital regions had the greatest Wavelet Entropy decrease. The source analysis was performed with MNE and complemented the results acuity, indicating that the major brain areas affected by MBSR were the left middle occipital lobe, precuneus and the superior temporal lobe. Nevertheless, half of the recruited participants already had some experience in other meditation techniques. Furthermore, Kakumanu et al. (2018) also reported decreased complexity (as measured by permutation entropy and Higuchi's fractal dimension) in meditators of different levels of expertise in Vipassana (CDM and ADM). This study compared the meditators to novices, not only during meditation, but also at baseline, reinforcing the long term effects of the practice (trait). Moreover, an inverse relationship was established between meditation experience and complexity changes, i.e., high complexity positively correlated with delta, beta and low-gamma and negatively correlated with theta-alpha and conversely, long term experience negatively correlated with these bandwidths. Nevertheless, the authors indicated slightly increased complexity in more experienced practitioners during distinct meditative practices contrasted to less experienced ones (state effects), albeit all participants were considered proficient.

Another way to evaluate brain complexity is to measure the variability of the neuronal oscillations, which extends across a wide range of time scales with no characteristic or typical scale. This is done by testing for long-range temporal correlations (LRTC). LRTC are the scale-free decay of temporal (auto)correlations in the amplitude envelope of neuronal oscillations (Linkenkaer-Hansen et al., 2001; Hardstone et al., 2012). Indeed, several studies have observed hallmarks of critical dynamics, showing scale-free fluctuations in neuronal oscillations with long-range temporal correlations (Linkenkaer-Hansen et al., 2001) and spatio-temporal correlations (Eguiluz et al., 2005; Expert et al., 2010; Tagliazucchi et al., 2012). Irrmischer et al. (2018) tested whether subjectively perceived mental stability during CDM-FA meditation is reflected as neuronal dynamics alterations. The method used was the Detrended Fluctuation Analysis (DFA) (Peng et al., 1995), which provides a quantitative index of scale-free amplitude modulation of neuronal oscillations. The authors found that the temporal complexity of brain-activity fluctuations, quantified with LRTC, was strongly suppressed in experienced meditators and related to trait absorption, whereas meditation-naïve healthy volunteers were unable to achieve this brain state when given the same instruction to focus on their breath. A sustained practice over a period of a year increased the effect and affected normal waking brain dynamics, as reflected in increased LRTC during a closed-eyes resting state, indicating that brain dynamics were altered beyond the momentary meditative state.

Yet, Huang and Lo (2009) reported increased complexities (measured with the complexity index) over the occipital and temporal areas related with a higher beta activity in a group of Zen practitioners (CDM-FA) compared to a control group at rest. The authors suggested that higher complexity correlates with the strength of neural connectivity, which can be translated into a “higher state of consciousness” in Zen meditators. Likewise, Vivot et al. (2020) hypothesize an increase in the meditators' complexity due to the enlarged repertoire of subjective experiences of experienced practitioners. They used the Hilbert transform to separate amplitude and phases related to EEG bandwidths computing the sample entropy in a group of breath-focusing controls in comparison to Vipassana, Himalayan Yoga, and Isha Shoonya practitioners (CDM). The authors reported increased complexity in alpha, low and high gamma, for Vipassana practitioners compared to controls, and in gamma band in Himalaya Yoga vs. controls. The distribution of gamma entropy in the scalp of Vipassana practitioners was further used for a random forest classifier, and enabled the detection of other meditative states.

We observed that although these techniques allow the investigation of more complex features and relations between signals, the studies have adopted slightly different proposals. Therefore, though they are not directly comparable, we could observe results pointing to distinct directions. Further studies exploring these methods are required. There is growing claim stating that brain dynamic studies would be much improved by the combined use of both linear and non-linear variables. In this direction the association between spectral features and complexity measures may be a promising approach.

## 9. Network Analysis

A topic of major interest in neuroscience concerns brain *functional connectivity*, i.e., how brain regions dynamically modify their interactions to process information. In the actual framework, signal analysis starts with the quantification of their interactions, via definition of a relation measure. Most measures analyze pairwise interactions, i.e., the interactions between pairs of signals are quantified and stored in a relation matrix. As a consequence, higher order effects are generally dismissed. Several features of brain activity can be used to measure interactions, ranging from a simple correlation to other proposals which include more complex features and directional interactions, like the Granger causality (Granger, 1969).

The relation matrix is subsequently analyzed as a network descriptor, through a mathematical model called *graph*, as detailed below. A graph is a simplified representation of objects, also called *nodes*, and the relationships they present with one another are named *links*. There is a mathematical field that specifically concerns graphs and their relations, called *Graph Theory*. Some concepts of graph theory have been translated to analyze brain functional connectivity, in a broad range of experimental scenarios (Rubinov and Sporns, 2010).

The graph theory has a set of parameters that aim to quantify numerically network efficiency. The first set of parameters analyze all possible routes of information flow between nodes, called *paths*. They are based on the consideration that stronger links relate to stronger interactions and, therefore, are more likely to be employed in transference of information. Thus, shorter paths are considered more efficient than long paths. Among the considered parameters are *diameter*, which corresponds to the longest network path and inversely correlates with network integration. In addition, another parameter is network *mean path length* the smaller, the better. Similarly, the average within the inverse shortest path lengths is called *global efficiency* and this quantum as calculated for each node is called *local efficiency*. Another parameter is the *clustering coefficient* and computes the number of *motifs*, i.e., triangular organizations, within the network. The rationale is to estimate whether the node's neighbors are also neighbors to each other. Moreover, the *network small worldness* quantifies the balance among functional modules assembled in the network and the links that join them. Similarly, one can estimate the influence that each specific node has in the network, based on the assumption that central nodes participate in several short paths and have a major role in information traffic. Some related quantifiable parameters are *betweenness centrality*, which computes the fraction of all the shortest paths through one given node; and *eccentricity*, corresponding to the longest distance (in number of links) between two specific nodes. The *average eccentricity* corresponds to the mean value among all nodes. For more information, the reader is referred to Rubinov and Sporns (2010). In this section, we covered some meditation studies that aimed to comprehend the impact of meditation on brain interactions as analyzed by graph theory parameters.

Regarding the analyses of meditative states in comparison to baseline conditions, Hauswald et al. (2015) investigated network measurements in zen meditators during meditation (CDM-OM) and at rest with open eyes. Initially, they applied the DICS, followed by the use of the imaginary part of the coherence in order to quantify the interactions. The resultant relation matrix was pruned and the authors reported correlations between the scores of a mindfulness questionnaire with the resultant network small worldness and clustering coefficient in a high gamma bandwidth (160–170 Hz), a frequency little explored in EEG studies on meditation. Moreover, during meditation, the networks at another high gamma bandwidth (200–210 Hz) presented significant lower small worldness and increased clustering coefficients in paracentral, insular, and thalamic regions.

On the other hand, van Lutterveld et al. (2017) evaluated trait effects, by comparing the connectivity of effortless awareness (CDM-OM) practitioners vs. novices. Each electrode was considered a node and the PLI was used as a relation measure. In sequence, instead of establishing an arbitrary threshold to bin the matrix, they employed the Minimum Spanning Tree algorithm (Stam et al., 2014), to extract the network core. The algorithm derives a sub graph out of the original relation matrix, including the strongest interactions while avoiding loops. The betweenness centrality was higher in the alpha band for practitioners, whereas the diameter and average eccentricity were lower, supporting the notion that increased integration occurs in the brain network of meditators. These parameters did not change either in theta or in beta.

Jiang et al. (2020) evaluated both state and trait effects, by using the imaginary coherence to estimate the functional connectivity between voxels resultant of the Source reconstruction in heartbeat-EP of CDM practitioners and controls. These relation matrices were calculated for each frequency band (from delta to gamma), and each connection in the graph was evaluated for statistical significance by two sample *t*-tests using network-based statistics (Zalesky et al., 2010). The pruned networks indicated decreased mean connectivity in gamma band in the frontal-limbic network as a state effect (meditators at practice contrasted to baseline) and decreased parietal-central and parietal-limbic network connectivity in the theta band as trait effect (meditators at baseline contrasted to controls). The interactions between these two frequencies were analyzed by Cross-frequency directionality, which computes if the phase of a slow oscillation leads the amplitude of a faster one (Jiang et al., 2015), and the results indicated that the amplitude of gamma oscillations resets the phase of theta oscillations during meditation. Moreover, they were significant when characterizing state differences, but not trait.

Furthermore, some authors performed longitudinal analysis of network connectivity: Xue et al. (2014) analyzed the differences in the theta bandwidth in eyes-closed resting state meditation-based training (Integrative body-mind training—IBMT, classified as CDM-FA) and relaxation after 1 week of practice. They employed a relation measure called *synchronization likelihood* (Stam and Van Dijk, 2002). In this perspective, EEG signals are modeled as a set of chaotic systems and two systems are considered synchronized when the state of the response system is modulated by the state of the driving system. The synchronization likelihood is one proposal to measure the coupling between signals under this consideration. Next, the network was pruned at different thresholds and the authors reported larger clustering coefficient, global and local efficiency, and shorter average path length after training, which were related to meditation-induced functional network plasticity. Also, Lee et al. (2018a) used the imaginary coherence but in order to evaluate the DMN functional connectivity in controls and in a novice group before and after 4 weeks of mind-body training protocol (CDM). The cortical activity was estimated with DICS in different bandwidths, and their correspondent relation matrix was averaged between groups; thus, the higher these values, the stronger the functional connectivity. The results pointed to increased global DMN strength in meditators in the theta and alpha bands.

Finally, Ghaderi et al. (2018) used graph theory to evaluate time perception by comparing epochs of body scan meditation (CDM-FA) in two groups. The relation matrix was computed with the coherence operator in delta and in four beta sub-bands, taking each electrode as a node. After that, the matrix was pruned with several thresholds and the resultant graphs were assessed. The authors reported that the clustering coefficient was higher in the beta-3 sub-band in the group that overestimated time in contrast to the group that underestimated it. These results suggest that meditation alters time perception neuronal mechanisms, but the authors did not contrasted it with the baseline data.

To the date of this review, there are few published studies using network analysis to evaluate meditation practice, with considerate variation of methodologies. However, this is a promising approach to the understanding of functional relation between brain regions and of the influence that lie behavioral changes.

## 10. Multimodal Studies

A trend in neurophysiology studies aims at combining multiple techniques of signal acquisition, in a way that their individual strengths are combined to overcome their limitations. These experiments are called *multimodal*, and allow the experimenter to improve the characterization of phenomena (Keil et al., 2014).

In the meditation field, multimodal approaches are seen since the first studies appearing in the 70's, which aimed at incorporating complementary information from peripheral physiology, like skin resistance, heart rate and respiration (Wallace, 1970; Wallace et al., 1971; Elson et al., 1977; Fenwick et al., 1977; Hebert and Lehmann, 1977; Corby et al., 1978). Recently, new analyses have combined these signals (Gao et al., 2016; Chandra et al., 2017; Sik et al., 2017; Jiang et al., 2020).

Moreover, recent technology allowed the combination of EEG with other recording modalities of the central nervous system activity. Yamamoto et al. (2006) combined the magnetoencephalogram (MEG) with EEG, due to the former's higher spatial precision. In their study, a group performed TM meditation (NDM) while the other executed a sham mantra; the investigators searched for the source of the stronger frontal and central alpha activity seen in the meditation group. The activity was in the medial PFC and in the ACC, corroborating with the same areas reported in fMRI studies. Yu et al. (2011) also investigated the PFC, but instead combined EEG with near-infrared spectroscopy (NIRS), to evaluate regional hemodynamic changes during Zen meditation (classified as CDM-FA). Their spectral findings indicated increased alpha and decreased theta activities, which were accompanied by increased levels of oxyhemoglobin, and, therefore, pointed to increased brain activity in the anterior PFC. On the other hand, Tang et al. (2009) findings were in the ACC: they performed two distinct experiments comparing short CDM meditation and relaxation protocols, acquiring data before, during and after 5 days of training. Part of the participants (further subdivided in intervention and control) was imaged with single photon emission computed tomography (SPECT), and the data indicated stronger ACC activity in the meditation group. Physiological data were gathered from the other part of participants (also subdivided in two groups), and the frontal midline theta power was related with ACC activity.

Finally, as another approach, the EEG was combined with fMRI, allowing the relative lack of spatial resolution of the EEG to be compensated by the fMRI. Meanwhile, one can improve the fMRI temporal resolution. Hagerty et al. (2013) carried out a case-study of an experienced CDM-FA practitioner but still without simultaneous acquisitions. Panda et al. (2016) used EEG-informed fMRI in a simultaneous acquisition protocol to study experienced Raja Yoga (also classified as CDM-FA) meditators in comparison to healthy controls in resting (closed-eyes) and during meditation. The analysis used the so-called EEG *microstates*, transient scalp topographic patterns that last around milliseconds, that some authors associate to fundamental units of brain neural processing (Lehmann et al., 1987). They selected a particular microstate, observed in all subjects, and used it to inform the General Linear Model (GLM) of fMRI analysis pipeline. The authors identified a microstate class that had already been previously related to DMN and showed that both the duration and the frequency of this DMN-microstate increased in meditators at rest and during meditation. Moreover, they showed that the DMN-microstate duration in meditators when at rest and engaged in the practice converges with longer practice time, which suggests that practice leads to convergence of meditation state and trait. However, Faber et al. (2017) showed increased coverage and occurrence in the DMN-microstate during periods of undirected mentation of TM practice when compared to transcendent periods and baseline (closed-eyes). Thus, summarizing, these findings indicate that meditation influences the brain temporal dynamics.

In addition to the monitoring of central nervous systems physiology, we also recall some attempts to directly influence meditation neural correlates and enhance behavioral results. For example, Hunter et al. (2018) used transcranial direct current stimulation (tDCS) together with Mindfulness (CDM-FA/OM) as a way to improve working memory, and Luft et al. (2019) used transcranial alternating current stimulation (tACS) to improve visual imagery during stabilizing (CDM-FA) and analytical meditations (NDM).

## 11. Discussion

The practices of meditation are associated with improvements of mental and physical health and, thus, have received growing attention from the scientific community. These practices are particularly interesting to the field of public health, due to their low cost, non-invasiveness, and potential scalability. Most people benefit from these practices, however precautions should be taken for patients with mental disorders (Van Dam et al., 2018). Nevertheless, there are several fundamental conceptual and methodological issues, relevant for consideration during the scrutiny of meditation, mostly due to inherent subjective features that encompass the practice and the challenges to measure them (Davidson and Kaszniak, 2015). In parallel, there is also substantial disparity among studies regarding analytical approaches (Lomas et al., 2015; Van Dam et al., 2018). The myriad of experimental designs and analysis pipelines combined with the evaluation of a phenomenon with subjective characteristics should be properly coordinated in order to enable further generalizations.

Therefore, in this work, we performed a meticulous methodological review of the existing literature on electroencephalographic (EEG) recordings and meditation of healthy participants. The EEG is a non-invasive technique that allows researchers to record the electrical activity of the brain. Since it has a high temporal resolution it is suitable to understand how the instant activity of a myriad of neurons in the cortex is modified by external or internal stimuli. Meditation studies with EEG have been published since the 60's and the technique still remains broadly used as we extensively cited in the course of this work. The analysis methods used in meditation research are widely diverse, ranging from spectral analysis, event related potentials, synchrony, source analysis, non-linear analysis, network analysis to multimodal studies. Independent of analysis method or design, differences are consistently found between individuals who meditate and controls. The significance and interpretation of the findings depend on the background of the analysis method used.

As extensively reviewed in this work, the EEG offers a lot of experimental flexibility in terms of processing, but such great variety of parameters implies that results often cannot be compared. There is lack of standardization of signal processing techniques and of their parameters, of the meditation practices assessed with EEG, and also of the target population profile (scant description and definition). In fact, recent reviews have focused mostly on findings regarding the spectral outcomes (Lomas et al., 2015; Lee et al., 2018b) and were unable to generalize the findings which explore more complex features of the EEG signal. Lomas et al. (2015) stated in a systematic review of mindfulness meditation using EEG that one of the issues hindering a meta-analysis was the great diversity of methods: no more than three (3) works employed the same processing. These inconsistencies in the literature hinder the formulation of more specific hypotheses and restrict studies exploring complementary features of the EEG signal to an observational level.

Data arising from standardized protocols are essential to assess the persistence of effects, to address the incidence of possible negative outcomes, and to enable meta analyses of populational data (Davidson and Kaszniak, 2015; Fingelkurts et al., 2015; Van Dam et al., 2018). Guidelines for designing high quality randomized clinical trials which are the gold standard on healthcare studies, such as the CONSORT (Moher et al., 2001), should be adopted. By doing this, several methodological flaws in the meditation research area could be avoided, such as publication bias, small number of subjects, poorly selected or described control groups, randomization and data-dredging. Moreover, some published studies did not test a specific *a priori* hypothesis, with a yes/no answer, and/or did not state it clearly enough, establishing a direct relation to behavior, which compromise statistical analyses.

A standard protocol should extend beyond randomized clinical trial guidelines, but also encompass general recommendations and specifications for meditation practices, such as proposed by Van Dam et al. (2018), and specific EEG guidelines, such as the checklist elaborated by Keil et al. (2014). In summary, this checklist advises the definition and description of a minimal set of technical specifications, such as the number of electrodes, the cap setup (10–20, for example), the channels' impedance, the implementation or not of filtering and artifact removal techniques, the sampling rate, electrode types (active/passive), the reference, whether the montage is mono or bipolar, and the filters order/design. We emphasize the importance of equipment description, because some consumer-grade EEG devices have variability issues that may hugely impact their use in research (Maskeliunas et al., 2016). The protocol should also clearly define the type of meditation and the control condition (open or closed eyes, for example, greatly impacts EEG signals), the duration of the recordings, the subjects' meditation experience level and other populational variables of interest. Finally, we emphasize that some algorithms have slightly different outcomes depending on the selected data chunk, because of the intrinsic variations of EEG signals. Thus, it is also important to report meticulously the processing pipeline and how/if those variabilities were considered, aspects required to ensure robustness, and to enable discussions of the findings.

The application of distinct processing methodologies hinders the compilation of studies whose conclusions run in similar directions. Therefore, we firmly encourage experimental data and code sharing, a practice that would not only enable cross-check analysis by the community and spare resources, but also would allow new signal processing techniques to be evaluated on known datasets and with a larger number of subjects. Such actions become of major importance when we consider new trends on signal acquisition, like multimodal and synchronized acquisition, and their translation to meditation studies, thus requiring the standardization of a broader set of technical parameters.

Some efforts in this direction include *Machine Learning* algorithms, which consist of a series of proposals targeting pattern recognition and classification. For example, Hinterberger et al. (2011) used a linear classifier and could classify subjects in distinct pre-induced meditation states, while Ahani et al. (2013, 2014) using the Support Vector Machine (SVM) algorithm, associated EEG with respiration to discriminate whether stressed subjects engage or not in a 6-weeks intervention. In addition, Lee et al. (2017) also used the SVM and an artificial neuronal network (ANN) based on spectral features to classify meditation expertise of focused breathing practitioners (CDM-FA), and Sharma et al. (2019) employed ANN to differentiate meditators and non-meditators.

Notwithstanding the above experimental design aspects, the major emergent features common to the studies are: (i) the modulation of ERP components, in particular, the P300, reinforcing that meditation practice affects attention and emotional control; (ii) findings in alpha and theta bandwidths. The alpha bandwidth is associated with relaxation and memory consolidation (Klimesch et al., 1994; Hari and Salmelin, 1997; Başar et al., 1999; Nunez and Srinivasan, 2006) and the theta band is related with focused attention, cognitive processing, creativity, and memory but also with drowsiness (Schacter, 1977; Klimesch et al., 1994; Başar et al., 1999); and (iii) Modulations are found in areas associated to the DMN, which is related to emotional processing, autobiographical memories and self-referential mental activities (Raichle, 2015).

Findings in the gamma bandwidth become more commonly described after Lutz et al. (2004) reported increased phase synchrony during meditation. Nevertheless, back in 1973, Banquet (1973) had already reported increase in amplitude of some TM meditators at 40Hz. The gamma bandwidth is associated with complex cognitive states, such as attention, working memory, and perception, being also related to information processing and REM sleep (Jokeit and Makeig, 1994; Başar et al., 1999; Kopell et al., 2000; Steriade, 2006). However, some criticism is always present regarding findings in the gamma bandwidth, because of its intrinsic low signal to noise ratio.

We also consistently observed that meditation neural correlates differ from other consciousness states, such as sleep and hypnosis. Thus, we stress the importance of studies scrutinizing the features of the meditative state instead of analyzing it as an intervention. They focus on more specific aspects of the states of the mind during the practice, like using a mantra, attentional traits or the nourishment of positive feelings (e.g., love and kindness). These distinctions are of main importance and we feel that deeper probing, as well as including a larger and more diverse population, might enable us to better seek general neural correlates of the meditation experience or, at least, practice-specific correlates.

Different types of meditation are likely to have a different outcome in subjects. Within the framework of the neurophenomenological matrix model (Lutz et al., 2015) meditation practices differ in respect to phenomenological dimensions (e.g., clarity, stability, object orientation). Therefore, the skills being trained need to be specified, allowing the designation of their specific effects in neural correlates. Nevertheless, it has not yet been possible to gather, among published studies and techniques, sufficient standardized evidence. It is for this reason that distinct neural correlates were not evident in our review. We believe that due to the great variety of designs, only the most prominent features have so far emerged. Thus, standardized protocols and efforts in that direction should be coordinated, allowing a better resource management on meditation research to enable the description of more refined, complex and meditation-specific neural correlates.

## Author Contributions

CD and MR wrote the paper. RA, MA, and MI were responsible for the sections of source analysis, EP/ERP, and non-linear techniques, respectively. EK supervised the work. All authors revised the full article.

## Conflict of Interest

The authors declare that the research was conducted in the absence of any commercial or financial relationships that could be construed as a potential conflict of interest.

## Supplementary Material

Data Sheet 2 Overview of all articles analyzed in this review.
