# Supplementary material for: A Critical Analysis on Characterizing the Meditation Experience Through the Electroencephalogram
Source: Front Syst Neurosci. 2020 Aug 7;14:53. doi: 10.3389/fnsys.2020.00053 (PMC7427581; doi:10.3389/fnsys.2020.00053)
Supplement: Data Sheet 2 — Overview of all articles analyzed in this review. [file Data_Sheet_2.PDF]

Autors	Title	Year	Meditation	Meditation category (ADM,NDM,CDM)	Eyes open or closed?	Number electrodes	EEG reference	Number participants	Experience	Design	Method	Result
Anand, B., Chhina, G., and Singh, B	Some aspects of electroencephalographic studies in Yogis	1961	Raj Yoga	NDM<br>One practitioner CDM	NA (probably open)	NA	Both ear lobes	4	NA	Baseline vs meditation<br>2 Yogis meditated with hand in cold water and were compared to baseline	Visual inspection	Resting showed persistent alpha activity with increased amplitude modulation during meditation.<br><br>Alpha activity persisted with hands in the water.
Kasamatsu A, Hirai T	Electroencephalographic Study on the Zen Meditation (Zazen)	1966	Zen (Zazen)	CDM-FA	Eyes open	4	NA	48 (Zen) + 18 (Control)	20: 1-5 years<br>12: 5-20 years<br>16: over 16 years	Before, during and after meditation	Visual inspection	Appearance of alpha waves without regard to opened eyes: increase amplitude and decrease frequency with the progress of meditation.<br><br>Rhythmical theta train sometimes appear in the later stage of the meditation.<br><br>Findings correlated with experience in meditation
Wallace RK	Physiological effects of transcendental meditation	1970	TM	NDM	Eyes closed/ Eyes open	6	Ear	15	6 month-3 years	Pre - Meditation - Post<br>There were eyes open epochs before and after baselines	Multimodal<br>Spectral	Alpha: Increase in amplitude and uniformity. In 4 subjects alpha got slower and sometimes become theta for some time.<br><br>Rare theta trains<br><br>No habituation in Alpha blocking
Wallace RK, Benson H, Wilson AF	A wakeful hypometabolic physiologic state	1971	TM	NDM	Eyes open or closed	6	A2	36 (28 males)	0.25-108 months (mean 29.4)	Before, during and after meditation	Multimodal<br>Spectral	Increased amplitude in alpha waves - Central and frontal regions<br>occasinal theta wave activity
Banquet JP	Spectral analysis of the EEG in meditation	1973	TM	NDM	Eyes closed / eyes open	7	Linked ears	12 Meditations (9 males) and 12 control (3 control became meditition group after learning meditation)	9 mo - 5 ya	Baselines eyes open/closed-> Meditation -> Getting out of meditation/relaxation -> Eyes closed (focus on image or though)-> Baseline Eyes open	Spectral (FFT)	Alpha increase in amplitude and gets slower. Usually spread from ocipital to parietal<br><br>Theta burts. Start frontal and spread posterior<br><br>Beta appeared in 4 participants and was related with deeper states of meditition (trancendence).<br>Starts frontal and spread posterior<br><br>No habituation in Alpha blocking
Williams P, West M	EEG responses to photic stimulation in persons experienced at meditation	1975	TM	NDM	Eyes closed	4	bipolar recordings	10 control (all males)<br>9 meditators (all males)	10-72 months (average 31 months)	Photic stimulation in resting state eyes closed	NA (maybe visual inspection)	Smaller alpha blocking habituation in meditators than in control group
Tebecis, A. K.	A controlled study of the eeg during transcendental meditation: Comparison with hypnosis	1975	TM	NDM	Eyes closed/Eyes open	2	NA	Meditators: 14 (12 males)<br>Controls: 14 (8 males)	"at least some months". Mean 19.8 months	Meditation vs Hypnosis<br><br>they also acquired baseline with eyes open and closed	Spectral	Higher mean theta density, during both meditation and non-meditation. Nevertheless, the hypnosis group presented more theta than the meditation group.
Pagano RR, Rose RM, Stivers RM, Warrenburg S	Sleep during transcendental meditation	1976	TM	NDM	Eyes closed	3 (occipital, central and frontal)	M1	5	at least 2.5 years	Meditation vs Naps	NA (maybe visual inspection)	On average 19% of time in Sleep stage 1 and 17%, 23% in stage 3 and 27% in stages 3 and 4
Fenwick PBC, Donaldson S, Gillis PL, Bushman J, Fenton PGW, Perry I, Tilsley C, Serafinowicz H	Metabolic and EEG changes during transcendental meditation: An explanation.	1977	TM	NDM	Eyes closed	6	NA	11 Meditators<br>7 Controls (predominantly non-meditators)- Classic music<br>8 Controls-Fasting	"completed a course of instruction in TM"	Meditators: before, during and after meditation<br><br>Controls: fasting and listening classic music	Multimodal (oxygem consumption)<br>Visual inspection	No EEG findings- they were all related to drowsiness
Elson BD, Hauri P, Cunis D	Physiological changes in yoga meditation.	1977	Ananda Marga	NDM	Eyes Closed/ Eyes open	1	Ear	10 meditators and a professor with 54 months of practice; and 11 controls	(9-31 months of practice) and professor with 54 months	baselines with eyes closed and open-> Meditation -> baselines with eyes closed and open	Visual Criteria (technician experienced in using standardized sleep stage criteria)<br><br>Multimodal	Meditators had mor non-descending alpha-theta than controls and remained at a relative constant level
Hebert R, Lehmann D	Theta bursts: an EEG pattern in normal subjects practising the transcendental meditation technique	1977	TM	NDM	Eyes Closed	at least 10 scalp location (10/20 system) primarily in referential montages	NA	78 (60 males)	24-140 - mean 56.1 months<br>54 control subjects (35 males)	5-10 min EO before and after a period of about 25 min of meditation	Visual Observation<br>Spectral (Fourier)<br><br>Multimodal	From meditation records 286 criterionmeeting theta bursts were seen in 21 of the 78 subjects.
Akers TK, Tucker DM, Roth RS, Vidiloff JS	Personality correlates of EEG change during meditation	1977	Christian Meditation (listened to a tape recording of their regular instructor as he presented a topic of devotion)	CDM-FA	Eyes Closed	2 (O1 and O2)	NA	15 (all males)	NA	mental arithmetic, taped devotion, meditation in silence	NA (maybe visual inspection)	As many subjects decreased as increased their alpha production during meditation.
Morse DR, Martin JS, Furst ML, Dubin LL	A physiological and subjective evaluation of meditation, hypnosis, and relaxation	1977	TM	NDM	Eyes Closed	4 (right frontal, parietal, temporal and occipital)	Occipital region	Meditation 12<br>Hypnosys 12<br>Meditation and Hyp 12<br>Naive in both 12	Meditators (2 mo-5ye; mean 1.3ye)<br>Hypnosis (3mo-10ye; mean 1ye)<br>Meditators and Hypnosis (6mo-8ye; mean 3.8 ye)	6 conditions:<br>Alert<br>Relaxation<br>HeteroHypnosysRelaxation<br>HeteroHypnosysTask<br>AutoHypnosysRelaxation<br>Meditation (TM or simple word)	Multimodal<br>Visual inspection	Significant differences between task-hypnosis and relaxation-hypnosis. No significant differences were found between meditation and simple word meditation.

Autors	Title	Year	Meditation	Meditation category (ADM,NDM,CDM)	Eyes open or closed?	Number electrodes	EEG reference	Number participants	Experience	Design	Method	Result
Levine, P. H., Hebert, J. R., Haynes, C. T., and Strobel, U	Eeg coherence during the transcendental meditation technique	1977	TM	NDM	Eyes Closed/ Eyes open	10	No uniform convention on reference electrode placement was in force, with homolateral, contralateral, and linked ears being variously used.	7 Novice (5 males)<br>21 Experienced (10 males)	0-15 years	Eyes-open precontrol, eyes-closed precontrol, Meditation, eyes-closed postcontrol, eyes-open postcontrol	Coherence	Coherence between F3/F4, C3/C4, C3/F3 e C4/F4.<br><br>Increase in the height and/or incidence of coherence peaks in the alpha band with onset of meditation but without a marked decrease at the end of that period.<br><br>Spreading of coherence peaks to other - generally lower - frequencies during meditation.<br><br>Abrupt onset of strong coherence with the start of the meditation and abrupt decrease at the end of the technique.
Corby JC, Roth WT, Zarcone VP Jr, Kopell BS	Psychophysiological correlates of the practice of Tantric Yoga meditation	1978	Ananda Marga	NDM	Eyes Closed	1 (Cz)	Joined mastoids	3 groups of 10 naive subjects	Trainees - average of 2.1years<br>Experts - average of 4.4 years	3 conditions: 1) baseline;<br>2) withdraw condition - preparation;<br>3) Meditation	Multimodal<br><br>Spectral<br><br>EP (Auditory)	Meditators increase theta with meditation more than controls<br><br>Alpha power tended to increase as subjects meditated (n.s.)<br><br>EP: Compared N1, P2 and P3.<br>P2, P3 and N1 amplitudes progressively decreased for infrequent tones as subjects entered meditation, but P2 and P3 increased for frequent ones
Barwood TJ, Empson JA, Lister SG, Tilley AJ	Auditory evoked potentials and transcendental meditation	1978	TM	NDM	Eyes Open	1	Left mastoid	8	18 months - 6 years	3 Blocks of 50 stimuli<br>before, during and after meditation<br><br>1 block at sleep onset	EP (auditory)	Analysed: amplitude and latency of the N1, P1 and N2 peaks.<br>No consistent changes were noted between baseline and meditating AEPs and from meditation and light sleep
Warrenburg S, Pagano RR, Woods M, Hlastala M	A comparison of somatic relaxation and EEG activity in classical progressive relaxation and transcendental meditation	1980	TM	NDM	Eyes Closed	2 (C4, O2)	A1	Experienced meditators: 9 (3 males)<br>Experienced Relaxation: 9 (3 males)<br>Novice Relaxation: 9 (3 males)	Experienced meditators: mean 3.4 years<br>Experienced Relaxation: mean 6.4 years	Relaxation Group (experienced) X Meditation X novice relaxation<br><br>They had a baseline with eyes closed and red a book	Multimodal<br>Visual inspection	Subjects were awake even with eyes closed<br>Short duration, high amplitude theta bursts
McEvoy TM, Frumkin LR, Harkins SW	Effects of meditation on brainstem auditory evoked potentials	1980	TM	NDM	NA	1 (Cz)	Mastoid	5 (1 male)<br>5 controls	6-9 years	before and after meditation. Stimulus at different intensities	EP (auditory)	Compared: waves I, II, III, and V<br><br>No pre-postmeditation differences at low stimulus intensities.<br>Latency of the inferior collicular wave (wave V) increased following meditation at moderate intensities<br>Latency slightly decreased at higher stimulus intensities.
Banquet, J. and Lese´vre, N	Event-related potentials in altered states of consciousness	1980	Yoga meditation	NA	NA	9	Linked ears	10 experimental (meditators)<br>10 controls	NA	visual choice reaction time task.<br><br>choice reachtion time task pre and post meditation or resting states (for control group)<br>Go/No-Go task	ERP(Visual)	P200 was significantly higher in pre and post for the meditators group compared to controls<br><br>N120 was higher post meditation for the meditation group compared to controls<br><br>The average response time for the meditators group occur before the P300 peak and has a smaler latency when compared to controls
Dillbeck MC, Bronson EC	Short-term longitudinal effects of the transcendental meditation technique on EEG power and coherence	1981	TM	NDM	Eyes closed	4 (F3, F4, O1, O2)	Linked mastoids	15	2-week retreat<br>(2 practices a day)	Before and after retreat	Spectral (FFT)<br>Coherence	Increased coherence in frontal electrodes in alpha
Dillbeck, M. C., Orme-Johnson, D.W., andWallace, R. K	Frontal eeg coherence, h-reflex recovery, concept learning, and the tm-sidhi program	1981	TM	NDM	Eyes closed	11 (F3, F4, C3, C4, P3, P4, 01, 02, T3, T4, Cz, and EOG)	Linked ears	12 - Experimental<br>13 - Controls	Experimental group undertook a three month TM-Sidhi program	Subjects did a Concept-learning task before and after three months	Spectral (FFT)<br>Coherence<br>H-Reflex	Found a relationship in frontal EEG coherence and H-reflex recovery that improved after TM
Stigsby B, Rodenberg JC, Moth HB	Electroencephalographic findings during mantra meditation (transcendental meditation). A controlled, quantitative study of experienced meditators	1981	TM	NDM	Eyes Closed	10 (mas em montagens bipolares, needles)	NA	13 (8 males)	at least 2 years (up to 8 years)	baseline before and after, meditation, drawsiness, sleep onset and sleep	Multimodal<br><br>Visual Inspection<br><br>Spectral	The EEG frequency spectra constituted a continuum with increasing theta and delta activity and decreasing alpha activity as the participants tended to fall asleep.<br><br>No consistent EEG pattern associated with a successful or unsuccessful meditation
Becker DE, Shapiro D	Physiological responses to clicks during Zen, Yoga, and TM meditation	1981	Zen<br>Yoga<br>TM	NDM	Eyes closed	2 (Cz, Oz)	Right ear (A2)	30 meditators (10 TM, 10 Yoga, 10 Zen)<br>10 controls (attend)<br>10 controls (resting)	Zen: 3 to 20 yeas<br>Yoga: 3 to 8.5 years<br>TM: 3.5 to 10.5 years	meditation vs attend vs resting	EP (auditory)<br>Spectral<br>Multimodal	Alpha blocking and habituation in all groups<br>No difference for P200 and P300<br>Enhanced N100 amplitude in Yoga and TM
Farrow JT, Hebert JR	Breath suspension during the transcendental meditation technique	1982	TM	NDM	Eyes open/ Eyes closed	9 (F3, F4, C3, C4, T3, Pz, O1, O2, Oz)	Linked ears	1	16 years	Eyes open; eyes closed but not practicing TM; TM; Eyes closed but not practicing TM; Eyes open.	Spectral (FFT)<br>Coherence<br>Multimodal	Bursts of theta, sometimes accompanied by bursts in other frequencies<br>Theta power peaked after breath suspension<br>Alpha power increases<br><br>EEG coherence higher in theta, alpha and beta before and during pure consciousness, with a sharp decrease afterward

Autors	Title	Year	Meditation	Meditation category (ADM,NDM,CDM)	Eyes open or closed?	Number electrodes	EEG reference	Number participants	Experience	Design	Method	Result
Badawi K, Wallace RK, Orme-Johnson D, Rouzere AM	Electrophysiologic characteristics of respiratory suspension periods occurring during the practice of the Transcendental Meditation Program	1984	TM	NDM	Eyes closed	For some: 9 (F3, F4, C3, Cz, C4, T3, T4, O1, O2)<br><br>For others: 6 (F3, F4, C3, C4, O3, and O4)	Linked ears	54 meditators<br>30 meditators (active control- breath supression)<br>31 non-meditators	16-144 months<br>1-113 months (active control)	meditation vs rest	Spectral (FFT)<br>Coherence<br>Multimodal	Increased alpha power and theta spindles<br><br>EEG coherence increase during breath suspension (all bands) in meditation, not present in control
Heide FJ	Psychophysiological responsiveness to auditory stimulation during transcendental meditation	1986	TM	NDM	Eyes closed	1	NA	17 meditators<br>17 controls	8-66 months	Meditation (meditators) vs rest (controls)	Multimodal<br>Alpha blocking	No differences between groups
Dillbeck MC, Vesely SA	Participation in the transcendental meditation program and frontal EEG coherence during concept learning	1986	TM	NDM	Eyes closed	7 (F3, F4, C3, C4, O1, O2, EOG)	Linked ears	16 meditators<br>16 controls	47.2 months	Meditation (meditators) vs rest (controls)	Coherence	Higher alpha and beta coherence in frontal electrodes
Severtsen B, Bruya MA	Effects of meditation and aerobic exercise on EEG patterns	1986	"mantra meditation"	CDM - FA	Eyes open/ Eyes closed	NA	NA	5 meditators<br>5 exercice	Novice	Meditation vs Exercice group<br>All at recordings at rest	Spectral	No differences between groups
Gaylord, C., Orme-Johnson, D., and Travis, F	The Effects of the Transcendental Mediation Technique and Progressive Muscle Relaxation on Eeg Coherence, Stress Reactivity, and Mental Health in Black Adults	1989	TM	NDM	Eyes closed	6	Linked ears	Randomly assigned<br><br>Meditators: 25<br>Relaxation: 29<br>Control: 29	They took a 10 hours course over six days. They were encouraged to practice daily for 20 minutes and to meet a group once a week for 2.5 months.<br><br>They were posttested one year later	Resting with eyes open -> resting eyes closed -> practiced relaxation procedure and the control group was asked to relax as deeply as possible -> resting eyes closed second time-> resting eyes open second time	Multimodal<br>Coherence	No longitudinal changes in EEG
Zhang W, Zheng R, Zhang B, Yu W, Shen X	An observation on flash evoked cortical potentials and Qigong meditation	1993	Qigong-focusing on Dantian	CDM - FA	Eyes Open	1 (Oz)	Right ear (A2)	14 experienced<br>12 begginer experience<br>11 controls	experienced: 0.5-5.5 years<br>begginers: 0.5-3 months	Flashes before, during and after Qigong meditation	EP (visual)	VEP amplitudes increased during meditation in advanced practitioners
Telles S, Nagarathna R, Nagendra HR, Desiraju T	Alterations in auditory middle latency evoked potentials during meditation on a meaningful symbol--"Om"	1994	"Om" meditation	CDM-FA	Eyes closed	1 (Cz)	Left ear (A1)	9 meditators (all males)<br>9 naive (all males)	more than 10 years of experience (12.4 SD 1.8 years)	Sham meditation ("One"), Meditation ("Om"), baseline before and after	EP (auditory)	Evaluated wave V, Na and Pa<br><br>Meditators: increase in the peak amplitude of Na wave during meditation reduction in the Na wave peak amplitude during control sessions.<br><br>Naive: significant decrease in the Na wave peak amplitude during meditation sessions<br><br>No significant changes in short latency wave V or Pa wave
Travis F, Wallace RK	Autonomic patterns during respiratory suspensions: possible markers of Transcendental Consciousness	1997	TM	NDM	Eyes closed	19	NA	78 (60 males)	0.4-22.5 years (Mean 12.6)	three phases of TM: inward strole, Transcedental Consciousness, outward stroke	Multimodal<br>Spectral	Outward stroke not obtained for all participants<br><br>Difference between Transcendental Consciousness and inward stroke: decreased theta and increased alpha
Lee MS, Bae BH, Ryu H, Sohn JH, Kim SY, Chung HT	Changes in alpha wave and state anxiety during ChunDoSunBup Qi-training in trainees with open eyes	1997	Qigong - ChunDoSun Bup Qi	CDM-FA	Eyes open	6 (F3, F4, C3, C4, O1, O2)	Earlobes (A1 and A2)	13 (7 males)	1-3 years	Analysis of distinct states of Qigong. (2 of them have movements)	Spectral (FFT)	Increased alpha in occipital region
Anand, B., Chhina, G., and Singh, B	Some aspects of electroencephalographic studies in Yogis	1961	Raj Yoga	NDM<br>One practitioner CDM	NA (probably open)	NA	Both ear lobes	4	NA	Baseline vs meditation<br>2 Yogis meditated with hand in cold water and were compared to baseline	Visual inspection	Resting showed persistent alpha activity with increased amplitude modulation during meditation.<br><br>Alpha activity persisted with hands in the water.
Kasamatsu A, Hirai T	Electroencephalographic Study on the Zen Meditation (Zazen)	1966	Zen (Zazen)	CDM-FA	Eyes open	4	NA	48 (Zen) + 18 (Control)	20: 1-5 years<br>12: 5-20 years<br>16: over 16 years	Before, during and after meditation	Visual inspection	Appearance of alpha waves without regard to opened eyes: increase amplitude and decrease frequency with the progress of meditation.<br><br>Rhythmical theta train sometimes appear in the later stage of the meditation.<br><br>Findings correlated with experience in meditation
Wallace RK	Physiological effects of transcendental meditation	1970	TM	NDM	Eyes closed/ Eyes open	6	Ear	15	6 monthd-3 years	Pre - Meditation - Post<br>There were eyes open epochs before and after baselines	Multimodal<br>Spectral	Alpha: Increase in amplitude and uniformity. In 4 subjects alpha got slower and sometimes become theta for some time.<br><br>Rare theta trains<br><br>No habituation in Alpha blocking
Wallace RK, Benson H, Wilson AF	A wakeful hypometabolic physiologic state	1971	TM	NDM	Eyes open or closed	6	A2	36 (28 males)	0.25-108 months (mean 29.4)	Before, during and after meditation	Multimodal<br>Spectral	Increased amplitude in alpha waves - Central and frontal regions occasinal theta wave activity
Banquet JP	Spectral analysis of the EEG in meditation	1973	TM	NDM	Eyes closed / eyes open	7	Linked ears	12 Meditators (9 males) and 12 control (3 control became meditition group after learning meditation)	9 mo - 5 ya	Baselines eyes open/closed-> Meditation -> Getting out of meditation/relaxation -> Eyes closed (focus on image or though)-> Baseline Eyes open	Spectral (FFT)	Alpha increase in amplitude and gets slower. Usually spread from ocipital to parietal<br><br>Theta burts. Start frontal and spread posterior<br><br>Beta appeared in 4 participants and was related with deeper states of meditition (trancendence).<br>Starts frontal and spread posterior<br><br>No habituation in Alpha blocking

Autors	Title	Year	Meditation	Meditation category (ADM,NDM,CDM)	Eyes open or closed?	Number electrodes	EEG reference	Number participants	Experience	Design	Method	Result
Williams P, West M	EEG responses to photic stimulation in persons experienced at meditation	1975	TM	NDM	Eyes closed	4	bipolar recordings	10 control (all males)<br>9 meditators (all males)	10-72 months (average 31 months)	Photic stimulation in resting state eyes closed	NA (maybe visual inspection)	Smaller alpha blocking habituation in meditators than in control group
Tebecis, A. K.	A controlled study of the eeg during transcendental meditation: Comparison with hypnosis	1975	TM	NDM	Eyes closed/Eyes open	2	NA	Meditators: 14 (12 males)<br>Controls: 14 (8 males)	"at least some months". Mean 19.8 months	Meditation vs Hypnosis<br><br>they also acquired baseline with eyes open and closed	Spectral	Higher mean theta density, during both meditation and non-meditation. Nevertheless, the hypnosis group presented more theta than the meditation group.
Pagano RR, Rose RM, Stivers RM, Warrenburg S	Sleep during transcendental meditation	1976	TM	NDM	Eyes closed	3 (occipital, central and frontal)	M1	5	at least 2.5 years	Meditation vs Naps	NA (maybe visual inspection)	On average 19% of time in Sleep stage 1 and 17%, 23% in stage 3 and 27% in stages 3 and 4
Fenwick PBC, Donaldson S, Gillis PL, Bushman J, Fenton PGW, Perry I, Tilsley C, Serafinowicz H	Metabolic and EEG changes during transcendental meditation: An explanation.	1977	TM	NDM	Eyes closed	6	NA	11 Meditators<br>7 Controls (predominantly non-meditators)- Classic music<br>8 Controls-Fasting	"completed a course of instruction in TM"	Meditators: before, during and after meditation<br><br>Controls: fasting and listening classic music	Multimodal (oxygem consumption)<br>Visual inspection	No EEG findings- they were all related to drowsiness
Elson BD, Hauri P, Cunis D	Physiological changes in yoga meditation.	1977	Ananda Marga	NDM	Eyes Closed/ Eyes open	1	Ear	10 meditators and a professor with 54 months of practice; and 11 controls	(9-31 months of practice) and professor with 54 months	baselines with eyes closed and open-> Meditation -> baselines with eyes closed and open	Visual Criteria (technician experienced in using standardized sleep stage criteria)<br><br>Multimodal	Meditators had mor non-descending alpha-theta than controls and remained at a relative constant level
Hebert R, Lehmann D	Theta bursts: an EEG pattern in normal subjects practising the transcendental meditation technique	1977	TM	NDM	Eyes Closed	at least 10 scalp location (10/20 system) primarily in referential montages	NA	78 (60 males)	24-140 - mean 56.1 months<br>54 control subjects (35 males)	5-10 min EO before and after a period of about 25 min of meditation	Visual Observation<br>Spectral (Fourier)<br><br>Multimodal	From meditation records 286 criterionmeeting theta bursts were seen in 21 of the 78 subjects.
Akers TK, Tucker DM, Roth RS, Vidiloff JS	Personality correlates of EEG change during meditation	1977	Christian Meditation (listened to a tape recording of their regular instructor as he presented a topic of devotion)	CDM-FA	Eyes Closed	2 (O1 and O2)	NA	15 (all males)	NA	mental arithmetic, taped devotion, meditation in silence	NA (maybe visual inspection)	As many subjects decreased as increased their alpha production during meditation.
Morse DR, Martin JS, Furst ML, Dubin LL	A physiological and subjective evaluation of meditation, hypnosis, and relaxation	1977	TM	NDM	Eyes Closed	4 (right frontal, parietal, temporal and occipital)	Occipital region	Meditation 12<br>Hypnosys 12<br>Meditation and Hyp 12<br>Naive in both 12	Meditators (2 mo-5ye; mean 1.3ye)<br>Hypnosis (3mo-10ye; mean 1ye)<br>Meditators and Hypnosis (6mo-8ye; mean 3.8 ye)	6 conditions:<br>Alert<br>Relaxation<br>HeteroHypnosysRelaxation<br>HeteroHypnosysTask<br>AutoHypnosysRelaxation<br>Meditation (TM or simple word)	Multimodal<br>Visual inspection	Significant differences between task-hypnosis and relaxation-hypnosis. No significant differences were found between meditation and simple word meditation.
Levine, P. H., Hebert, J. R., Haynes, C. T., and Strobel, U	Eeg coherence during the transcendental meditation technique	1977	TM	NDM	Eyes Closed/ Eyes open	10	No uniform convention on reference electrode placement was in force, with homolateral, contralateral, and linked ears being variously used.	7 Novice (5 males)<br>21 Experienced (10 males)	0-15 years	Eyes-open precontrol, eyes-closed precontrol, Meditation, eyes-closed postcontrol, eyes-open postcontrol	Coherence	Coherence between F3/F4, C3/C4, C3/F3 e C4/F4.<br><br>Increase in the height and/or incidence of coherence peaks in the alpha band with onset of meditation but without a marked decrease at the end of that period.<br><br>Spreading of coherence peaks to other - generally lower - frequencies during meditation.<br><br>Abrupt onset of strong coherence with the start of the meditation and abrupt decrease at the end of the technique.
Corby JC, Roth WT, Zarcone VP Jr, Kopell BS	Psychophysiological correlates of the practice of Tantric Yoga meditation	1978	Ananda Marga	NDM	Eyes Closed	1 (Cz)	Joined mastoids	3 groups of 10 naive subjects	Trainees - average of 2.1years<br>Experts - average of 4.4 years	3 conditions: 1) baseline;<br>2) withdraw condition - preparation;<br>3) Meditation	Multimodal<br><br>Spectral<br><br>EP (Auditory)	Meditators increase theta with meditation more than controls<br><br>Alpha power tended to increase as subjects meditated (n.s.)<br><br>EP: Compared N1, P2 and P3.<br>P2, P3 and N1 amplitudes progressively decreased for infrequent tones as subjects entered meditation, but P2 and P3 increased for frequent ones
Barwood TJ, Empson JA, Lister SG, Tilley AJ	Auditory evoked potentials and transcendental meditation	1978	TM	NDM	Eyes Open	1	Left mastoid	8	18 months - 6 years	3 Blocks of 50 stimuli<br>before, during and after meditation<br><br>1 block at sleep onset	EP (auditory)	Analysed: amplitude and latency of the N1, P1 and N2 peaks.<br>No consistent changes were noted between baseline and meditating AEPs and from meditation and light sleep

Autors	Title	Year	Meditation	Meditation category (ADM,NDM,CDM)	Eyes open or closed?	Number electrodes	EEG reference	Number participants	Experience	Design	Method	Result
Warrenburg S, Pagano RR, Woods M, Hlastala M	A comparison of somatic relaxation and EEG activity in classical progressive relaxation and transcendental meditation	1980	TM	NDM	Eyes Closed	2 (C4, O2)	A1	Experienced meditators: 9 (3 males)<br>Experienced Relaxation: 9 (3 males)<br>Novice Relaxation: 9 (3 males)	Experienced meditators: mean 3.4 years<br>Experienced Relaxation: mean 6.4 years	Relaxation Group (experienced) X Meditation X novice relaxation<br><br>They had a baseline with eyes closed and red a book	Multimodal<br>Visual inspection	Subjects were awake even with eyes closed<br>Short duration, high amplitude theta bursts
McEvoy TM, Frumkin LR, Harkins SW	Effects of meditation on brainstem auditory evoked potentials	1980	TM	NDM	NA	1 (Cz)	Mastoid	5 (1 male)<br>5 controls	6-9 years	before and after meditation. Stimulus at different intensities	EP (auditory)	Compared: waves I, II, III, and V<br><br>No pre-postmeditation differences at low stimulus intensities.<br>Latency of the inferior collicular wave (wave V) increased following meditation at moderate intensities<br>Latency slightly decreased at higher stimulus intensities.
Banquet, J. and Lese´vre, N	Event-related potentials in altered states of consciousness	1980	Yoga meditation	NA	NA	9	Linked ears	10 experimental (meditators)<br>10 controls	NA	visual choice reaction time task.<br><br>choice reachtion time task pre and post meditation or resting states (for control group)<br>Go/No-Go task	ERP(Visual)	P200 was significantly higher in pre and post for the meditators group compared to controls<br><br>N120 was higher post meditation for the meditation group compared to controls<br><br>The average response time for the meditators group occur before the P300 peak and has a smaler latency when compared to controls
Dillbeck MC, Bronson EC	Short-term longitudinal effects of the transcendental meditation technique on EEG power and coherence	1981	TM	NDM	Eyes closed	4 (F3, F4, O1, O2)	Linked mastoids	15	2-week retreat<br>(2 practices a day)	Before and after retreat	Spectral (FFT)<br>Coherence	Increased coherence in frontal electrodes in alpha
Dillbeck, M. C., Orme-Johnson, D.W., andWallace, R. K	Frontal eeg coherence, h-reflex recovery, concept learning, and the tm-sidhi program	1981	TM	NDM	Eyes closed	11 (F3, F4, C3, C4, P3, P4, 01, 02, T3, T4, Cz, and EOG)	Linked ears	12 - Experimental<br>13 - Controls	Experimental group undertook a three month TM-Sidhi program	Subjects did a Concept-learning task before and after three months	Spectral (FFT)<br>Coherence<br>H-Reflex	Found a relationship in frontal EEG coherence and H-reflex recovery that improved after TM
Stigsby B, Rodenberg JC, Moth HB	Electroencephalographic findings during mantra meditation (transcendental meditation). A controlled, quantitative study of experienced meditators	1981	TM	NDM	Eyes Closed	10 (mas em montagens bipolares, needles)	NA	13 (8 males)	at least 2 years (up to 8 years)	baseline before and after, meditation, drawsiness, sleep onset and sleep	Multimodal<br><br>Visual Inspection<br><br>Spectral	The EEG frequency spectra constituted a continuum with increasing theta and delta activity and decreasing alpha activity as the participants tended to fall asleep.<br><br>No consistent EEG pattern associated with a successful or unsuccessful meditation
Becker DE, Shapiro D	Physiological responses to clicks during Zen, Yoga, and TM meditation	1981	Zen<br>Yoga<br>TM	NDM	Eyes closed	2 (Cz, Oz)	Right ear (A2)	30 meditators (10 TM, 10 Yoga, 10 Zen)<br>10 controls (attend)<br>10 controls (resting)	Zen: 3 to 20 years<br>Yoga: 3 to 8.5 years<br>TM: 3.5 to 10.5 years	meditation vs attend vs resting	EP (auditory)<br>Spectral<br>Multimodal	Alpha blocking and habituation in all groups<br>No difference for P200 and P300<br>Enhanced N100 amplitude in Yoga and TM
Farrow JT, Hebert JR	Breath suspension during the transcendental meditation technique	1982	TM	NDM	Eyes open/ Eyes closed	9 (F3, F4, C3, C4, T3, Pz, O1, O2, Oz)	Linked ears	1	16 years	Eyes open; eyes closed but not practicing TM; TM; Eyes closed but not practicing TM; Eyes open.	Spectral (FFT)<br>Coherence<br>Multimodal	Bursts of theta, sometimes accompanied by bursts in other frequencies<br>Theta power peaked after breath suspension<br>Alpha power increases<br><br>EEG coherence higher in theta, alpha and beta before and during pure consciousness, with a sharp decrease afterward
Badawi K, Wallace RK, Orme-Johnson D, Rouzere AM	Electrophysiologic characteristics of respiratory suspension periods occurring during the practice of the Transcendental Meditation Program	1984	TM	NDM	Eyes closed	For some: 9 (F3, F4, C3, Cz, C4, T3, T4, O1, O2)<br><br>For others: 6 (F3, F4, C3, C4, O3, and O4)	Linked ears	54 meditators<br>30 meditators (active control- breath supression)<br>31 non-meditators	16-144 months<br>1-113 months (active control)	meditation vs rest	Spectral (FFT)<br>Coherence<br>Multimodal	Increased alpha power and theta spindles<br><br>EEG coherence increase during breath suspension (all bands) in meditation, not present in control
Heide FJ	Psychophysiological responsiveness to auditory stimulation during transcendental meditation	1986	TM	NDM	Eyes closed	1	NA	17 meditators<br>17 controls	8-66 months	Meditation (meditators) vs rest (controls)	Multimodal<br>Alpha blocking	No differences between groups
Dillbeck MC, Vesely SA	Participation in the transcendental meditation program and frontal EEG coherence during concept learning	1986	TM	NDM	Eyes closed	7 (F3, F4, C3, C4, O1, O2, EOG)	Linked ears	16 meditators<br>16 controls	47.2 months	Meditation (meditators) vs rest (controls)	Coherence	Higher alpha and beta coherence in frontal electrodes
Severtsen B, Bruya MA	Effects of meditation and aerobic exercise on EEG patterns	1986	"mantra meditation"	CDM - FA	Eyes open/ Eyes closed	NA	NA	5 meditators<br>5 exercice	Novice	Meditation vs Exercice group<br>All at recordings at rest	Spectral	No differences between groups
Gaylord, C., Orme-Johnson, D., and Travis, F	The Effects of the Transcendental Mediation Technique and Progressive Muscle Relaxation on Eeg Coherence, Stress Reactivity, and Mental Health in Black Adults	1989	TM	NDM	Eyes closed	6	Linked ears	Randomly assigned<br><br>Meditators: 25<br>Relaxation: 29<br>Control: 29	They took a 10 hours course over six days. They were encouraged to practice daily for 20 minutes and to meet a group once a week for 2.5 months.<br><br>They were posttested one year later	Resting with eyes open -> resting eyes closed -> practiced relaxation procedure and the control group was asked to relax as deeply as possible -> resting eyes closed second time-> resting eyes open<br>second time	Multimodal<br>Coherence	No longitudinal changes in EEG

Autors	Title	Year	Meditation	Meditation category (ADM,NDM,CDM)	Eyes open or closed?	Number electrodes	EEG reference	Number participants	Experience	Design	Method	Result
Zhang W, Zheng R, Zhang B, Yu W, Shen X	An observation on flash evoked cortical potentials and Qigong meditation	1993	Qigong-focusing on Dantian	CDM - FA	Eyes Open	1 (Oz)	Right ear (A2)	14 experienced<br>12 begginer experience<br>11 controls	experienced: 0.5-5.5 years<br>begginers: 0.5-3 months	Flashes before, during and after Qigong meditation	EP (visual)	VEP amplitudes increased during meditation in advanced practitioners
Telles S, Nagarathna R, Nagendra HR, Desiraju T	Alterations in auditory middle latency evoked potentials during meditation on a meaningful symbol--"Om"	1994	"Om" meditation	CDM-FA	Eyes closed	1 (Cz)	Left ear (A1)	9 meditators (all males)<br>9 naive (all males)	more than 10 years of experience (12.4 SD 1.8 years)	Sham meditation ("One"), Meditation ("Om"), baseline before and after	EP (auditory)	Evaluated wave V, Na and Pa<br><br>Meditators: increase in the peak amplitude of Na wave during meditation reduction in the Na wave peak amplitude during control sessions.<br><br>Naive: significant decrease in the Na wave peak amplitude during meditation sessions<br><br>No significant changes in short latency wave V or Pa wave
Travis F, Wallace RK	Autonomic patterns during respiratory suspensions: possible markers of Transcendental Consciousness	1997	TM	NDM	Eyes closed	19	NA	78 (60 males)	0.4-22.5 years (Mean 12.6)	three phases of TM: inward strole, Transcedental Consciousness, outward stroke	Multimodal<br>Spectral	Outward stroke not obtained for all participants<br><br>Difference between Transcendental Consciousness and inward stroke: decreased theta and increased alpha
Lee MS, Bae BH, Ryu H, Sohn JH, Kim SY, Chung HT	Changes in alpha wave and state anxiety during ChunDoSunBup Qi-training in trainees with open eyes	1997	Qigong - ChunDoSun Bup Qi	CDM-FA	Eyes open	6 (F3, F4, C3, C4, O1, O2)	Earlobes (A1 and A2)	13 (7 males)	1-3 years	Analysis of distinct states of Qigong. (2 of them have movements)	Spectral (FFT)	Increased alpha in occipital region
Travis F, Wallace RK	Autonomic and EEG patterns during eyes-closed rest and transcendental meditation (TM) practice: the basis for a neural model of TM practice	1999	TM	NDM	Eyes Closed	6 (F3, F4, C3, C4, P3, P4)	Linked mastoids	20 (13 males)	1.9-25.5 ya (Mean 9.9)	meditation vs resting (order counterbalanced)	Spectral (FFT)<br>Coherence	FFT: No significant main effects for condition with alpha percentage<br><br>Coherence: Higher anterior-posterior and frontal alpha coherence during TM practice
Dunn BR, Hartigan JA, Mikulas WL	Concentration and mindfulness meditations: unique forms of consciousness?	1999	"Concentration (focus on breath) and Mindfulness (letting one's attention freely move to varios objects and to notice these objects clearly)"	CDM - FA/OM	Eye Closed	19	Linked ears	10	8-10 weeks in concentration followed by 8-10 weeks in mindfulness	baseline -> Concentration Meditation -> Mindfulness	Spectral (FFT)	Mindfulness produced more delta, theta, alpha and beta 1 than concentration condition.
Travis F, Tecce JJ, Guttman J	Cortical plasticity, contingent negative variation, and transcendent experiences during practice of the Transcendental Meditation technique	2000	TM	NDM	Eyes Closed	3 (Fz, Cz and Pz)	NA	Non meditators: 14 (7 males)<br>Novice meditators: 13 (6 males)<br>Experencied meditators: 14 (8 males)	Novice: mean 1.1 year<br>Experencied: mean 8.53	3 groups.<br>No meditators<br>Novice meditators<br>Experencied meditators	ERP (CNV)	CNV has smallst value in non meditators and largest in experencied meditators
Kubota Y, Sato W, Toichi M, Murai T, Okada T, Hayashi A, Sengoku A	Frontal midline theta rhythm is correlated with cardiac autonomic activities during the performance of an attention demanding meditation procedure.	2001	Zen (Su-soko)	CDM-FA	Eyes Open	19	Electrodes in right hemisphere and Fz, Cz and Pz reffered to the right earlobe Electrodes in left hemisphere referred to the left earlobe	25 (11 males) - but only 12 were analysed (6 males)	none	Meditation vs baseline	Multimodal<br>Spectral (FFT)	Increased power in Theta signals over the mid-frontal areas.<br><br>No difference in occipital alpha
Travis F	Autonomic and EEG patterns distinguish transcending from other experiences during Transcendental Meditation practice	2001	TM	NDM	Eyes Closed	9	Linked ears	30 (21 males)	5.4 years	Recordings during meditation. There was a sound bell at spaced time intervals	Multimodal<br>Spectral<br>Coherence	In experiences classified as 'transcendence'<br><br>higher Alpha amplitude<br>higher Alpha coherence
Travis F, Olson T, Egenes T, Gupta HK	Physiological patterns during practice of the Transcendental Meditation technique compared with patterns while reading Sanskrit and a modern language	2001	TM	NDM	Eyes closed	6	Linked ears	18 (7 males)	NA	Meditation vs reading Sanskrit and other modern languages	Multimodal<br>Spectral (FFT)<br>Coherence	Alpha power and coherence were significantly higher when reading Sanskrit and during meditation practice, compared to reading modern languages.
Arambula P, Peper E, Kawakami M, Gibney KH	The physiological correlates of Kundalini Yoga meditation: a study of a yoga master	2001	Kundalini Yoga	CDM- FA	Eyes Closed	2 (O2, P4)	Right earlobe	1 male	32 years	Meditation vs baseline before and after practice	Multimodal<br>Spectral	Increase in alpha activity compared to pre and post baseline<br>Increase in theta immediately following meditation

Autors	Title	Year	Meditation	Meditation category (ADM,NDM,CDM)	Eyes open or closed?	Number electrodes	EEG reference	Number participants	Experience	Design	Method	Result
Aftanas LI, Golocheikine SA	Human anterior and frontal midline theta and lower alpha reflect emotionally positive state and internalized attention: high-resolution EEG investigation of meditation.	2001	Sahaja Yoga	CDM-OM	Eyes closed	62	Tip of the nose	Short-term: 11 (5 males)<br>Long-term: 17 (7 males)	Short-term: less than 6 months<br>Long-term: 3-7 years	Meditation vs baseline before and after practice	Spectral (FFT)<br>Coherence	Increased anterior frontal and midline theta synchronization as well as theta long range connectivity between prefrontal and posterior association cortex.
Lehmann D, Faber PL, Achermann P, Jeanmonod D, Gianotti LR, Pizzagalli D	Brain sources of EEG gamma frequency during volitionally meditation-induced, altered states of consciousness, and experience of the self	2001	Several tibetan meditations	CDM- FA<br>NDM	Eyes Open	27	NA	1	Long-term buddhist lama	5 different practices<br>1) Buddha in front meditator<br>2) Buddha above meditator<br>3) Internal verbalization of mantra<br>4) Self-dissolution<br>5) Self-reconstruction	Source (Dipole and LORETA)	Analysis in Gamma<br>Dipole: the locations differed from each other significantly along at least one of the three dimensions in the comparisons between all meditations except for self-dissolution vs. self-reconstitution.<br><br>LORETA: Each meditation has different sources (except self-dissolution and self-reconstruction).
Aftanas LI, Golocheikine SA	Non-linear dynamic complexity of the human EEG during meditation	2002	Sahaja Yoga	CDM-OM	Eyes closed	62	Tip of the nose	20 (9 males)	experienced (NR)	Resting vs Meditation	Non-Linear (Dimensional Complexity-DCx)	DCx estimates negatively correlate with theta-2 and alpha-1, and positively with beta-3.
Travis F, Tecce J, Arenander A, Wallace RK	Patterns of EEG coherence, power, and contingent negative variation characterize the integration of transcendental and waking states	2002	TM	NDM	Eyes closed	9	Linked ears	51<br>No meditators: 17 (9 males)<br>Median experience: 17 (9 males)<br>Most experience: 17 (9 males)	Median experience: mean 7.8 year<br>Most experience: mean 24.5 years	Analysis of periods away from meditation, to asses only long-term effects.<br><br>CNV task -> rest eyes closed/ meditatiom	ERP (CNV)<br>Spectral (FFT)<br>Coherence<br>Linear regression	In most experienced individuals, CNV was higher in simple but lower in choice<br>trials, and 6/12 Hz EEG amplitude and broadband frontal EEG coherence were higher during choice trials.<br><br>Increased EEG amplitude and coherence, characteristic of TM practice, appeared to become a stable EEG trait during CNV tasks in these subjects. These significant EEG differences may underlie the inverse patterns in CNV amplitude seen between groups.<br><br>CNV amplitudes are highest in the simple trials and lowest in the choice trials for the most experienced group relative to the median experience one
Aftanas LI, Golosheikin SA	Changes in cortical activity in altered<br>states of consciousness: the study of meditation by high-resolution EEG.	2003	Sahaja yoga	CDM-OM	Eyes closed	62	Tip of the nose	Novice meditators (NMs): 11 (5 males)<br>Experienced Meditators (EMs): 16 (6 males)	Novice meditators (NMs): < 6months<br>Experienced Meditators (EMs): 3-6 years	Rest vs<br>three sequential meditation stages:<br>1- enter into meditation<br>2- deep meditation<br>3- exit from meditation	Spectral (IAF)<br>Coherence	EMs in resting state: Lower IAF<br>Higher θ-, $\alpha 1$-, $\alpha 2$-, and $\alpha 3$-band power values compared to NMs<br><br>Effective achievement of altered states of consciousness in EMs: associated with an increase in the local θ- and $\alpha 1$ powers in the anterior cortical areas, as well as long-distance coherence between the prefrontal and posterior associative cortex with the formation of a center of gravity in the left prefrontal region (lead AF3)
Davidson RJ, Kabat-Zinn J, Schumacher J, Rosenkranz M, Muller D, Santorelli SF, Urbanowski F, Harrington A, Bonus K, Sheridan JF	Alterations in brain and immune function produced by mindfulness meditation	2003	MBSR	CDM - FA/OM	Eyes open/ Eyes closed	27 (only used 8)	Linked ears	25 meditation group (6 males)<br>16 waiting list (6 males)	8 weeks	randomized controlled study<br><br>before, immediately after, and 4 months after program	Spectral (α asymmetry)	Anterior activation asymmetry in alpha as a function of meditation training
Murata T, Takahashi T, Hamada T, Omori M, Kosaka H, Yoshida H, Wada Y	Individual trait anxiety levels characterizing the properties of zen meditation	2004	Zen (Su-soko)	CDM- FA	Eyes open	4	linked ears	22	No experience	Supine position<br><br>Meditation vs synchronized breath	Multimodal<br>Coherence	Slow alpha interhemispheric coherence in the frontal region significantly increased during meditation
Lutz A, Greischar LL, Rawlings NB, Ricard M, Davidson RJ	Long-term meditators self-induce high-amplitude gamma synchrony during mental practice	2004	Loving-kindness	ADM	NA	128	Cz	8 Long term pratitioners<br>10 healthy students	15-40 years<br>No experience	30s Resting - 60s meditation (4x)	Spectral (FFT)	High amplitude gamma oscillations in EEGs, not present in baseline, in long-term pratitioners
Takahashi T, Murata T, Hamada T, Omori M, Kosaka H, Kikuchi M, Yoshida H, Wada Y	Changes in EEG and autonomic nervous activity during meditation and their association with personality traits	2005	Zen (Su-soko)	CDM-FA	Eyes open	6	Linked ears	20 all males	Novices	Supine position<br><br>Meditation vs synchronized breath	Multimodal<br>Spectral (FFT)	Power Spectra. Increase in power of Alpha 1 and theta 2, in particular on frontal electrodes.<br><br>No Alpha power change in occipital electrodes, indicating the subjects were not drawsy
Aftanas L, Golosheykin S	Impact of regular meditation practice on EEG activity at rest and during evoked negative emotions	2005	Sahaja Yoga	CDM-OM	Eyes open/ Eyes closed	62	Tip of nose	25 meditators (12 males)<br>25 controls (13 males)	5-10 years	Eyes closed, eyes open, neutral and emotional movie clip conditions	Spectral (FFT)	EEG data collapsed into 9 regional means<br><br>At eyes closed the meditation group had larger power values in theta1, theta 2 and alpha1 bands.<br><br>Theta 2 and alpha 1 remmained higher with eyes open<br><br>Alpha 1 remained higher when viewing neutral movie clip

Autors	Title	Year	Meditation	Meditation category (ADM,NDM,CDM)	Eyes open or closed?	Number electrodes	EEG reference	Number participants	Experience	Design	Method	Result
Lehmann D, Faber PL, Gianotti LR, Kochi K, Pascual-Marqui RD	Coherence and phase locking in the scalp EEG and between LORETA model sources, and microstates as putative mechanisms of brain temporo-spatial functional organization	2006	Ch'an	CDM - FA	NA	27	Recomputation for different references Coherence was computed between all 351 possible pairs of the 27 electrodes, and for each electrode combination.	1	"very experencied"	Resting vs Meditation	Source (LORETA)<br>Synchrony (PLV)<br>Coherence in LORETA ROI	Coherence is higly dependent on reference, should use intracerebral time series.
Yamamoto S, Kitamura Y, Yamada N, Nakashima Y, Kuroda S	Medial profrontal cortex and anterior cingulate cortex in the generation of alpha activity induced by transcendental meditation: a magnetoencephalographic study	2006	TM	NDM	Eyes closed	More than 6	Linked ears	8 control (4 males)<br>8 meditators (4 males)	Average 3.9	Sham meditation vs TM meditation (20 min)	Multimodal (MEG)<br>Spectral (FFT)	Higher Alpha in frontal area during TM
Travis F, Arenander A	Cross-sectional and longitudinal study of effects of transcendental meditation practice on interhemispheric frontal asymmetry and frontal coherence	2006	TM	NDM	Eyes closed	9	Linked ears	Study 1:<br>13 control group (5 male)<br>12 TM group (6 male)<br><br>Study 2:<br>14 (9 male)	Study 1<br>Average 21.9 years	eyes closed resting<br>TM practice<br>Reaction task	Spectral (Power, α asymmetry)<br>Coherence	Frontal coherence increased with time during TM practice
Sarang, S. and Telles, S	Changes in p300 following two yoga-based relaxation technique	2006	Cyclic meditation	CDM	Eyes closed	3 (Fz, Cz, Pz)	Linked earlobes	42 (all male)	More than 3 months (15.5 months SD 13.3)	Oddball (recorded before and after meditation or resting state)	ERP (Auditory)	Significant decrease in P300 peak latencies and increase in the amplitude post meditation practice compared to pre.<br>During resting state they observed a significant decrease in peak latency but no changes in the amplitude in post compared to pre.<br>Peak latencies were not difference on the pre measurement comparing meditation and resting, but they were significantly different on the post measurement
Slagter, Heleen A. and Lutz, Antoine and Greischar, Lawrence L. and Francis, Andrew D. and Nieuwenhuis, Sander and Davis, James M. and Davidson, Richard J.	Mental training affects distribution of limited brain resources	2007	Vipassana meditation	CDM-OM	Eyes open	1 (Pz)	average of both mastoids	17 practitioners (7 male)<br>23 matched controls (9 males)	3 months of intensive Vipassana meditation retreat for practitioners. Controls received a 1hr meditation class and were asked to meditate for 20 min daily for 1 week prior to each session	attentional-blink task (pre and post retreat)	ERP (Visual)	Significant decrease of P3b amplitude for the first stimulli after the retreat for the practitioners. The data was corroborated also with behavioral results. Where intensive mental training was associated with reduction in attentional blink size.<br>ERP was calculated ofer Pz
Srinivasan N, Baijal S	Concentrative meditation enhances preattentive processing: a mismatch negativity study	2007	Sudarshan Kriya Yoga	CDM-FA	NA	64	NA	10 meditators<br>10 non-meditator controls	3-7 years	Mismatch negativity<br>Recordings performed before and after each of 3 stages of meditation practice. The first 2 are breathing exercices and the last one is concentrative meditation.<br><br>Controls were asked to relax and read a book	ERP (auditory)	Meditators has larger mismatch negativity (MMN) amplitude than non meditators, with incease after the concentrative meditation.
Chan AS, Han YM, Cheung MC	Electroencephalographic (EEG) measurements of mindfulness-based Triarchic body-pathway relaxation technique: a pilot study	2008	Triarchic body-pathway relaxation technique (TBRT)	ADM	Eyes closed	19	Mean of electrodes	19 (8 males)	novices	Meditation and Baseline vs Music and Baseline	Spectral (FFT)<br>Source (LORETA)	TBRT with greater relative theta power in both anterior and posterior regions of<br>the brain compared with the music condition.<br><br>LORETA: increased theta activity during TBRT was generated by the anterior cingulate cortex<br><br>TBRT and music: both with increases in left-sided anterior activation
Eskandari P, Erfanian A	Improving the performance of brain-computer interface through meditation practicing	2008	TM	NDM	Eyes closed	NA	Right Earlobe	10<br>5 meditators<br>5 Control	about 1 year	BCI	ERP (Event Related Spectral Perturbation - ERSP)	The classification accuracy of the meditation group was 98% against 78% in control group
Cahn BR, Polich J	Meditation (Vipassana) and the P3a event-related brain potential	2008	Vipassana	CDM-OM	Eyes closed	19 (only uses 3)	Balanced linked earlobes	16 (11 males)	2.5-40<br>Mean: 20	Oddball	ERP (auditory)	N1 amplitude decreased in meditation compared to control to distracter stimuli. N1 did not differ for oddball stimuli.<br>P3a amplitude reduce in meditation for distracter.
Qin Z, Jin Y, Lin S, Hermanowicz NS	A forty-five year follow-up EEG study of Qigong practice	2009	Qigong	CDM-FA	Eyes closed	19	Linked earlobes	3 meditators (all males)<br>6 controls (4 males)	Practitioner 1: since late nineteen-fifties.<br>Practitioner 2: 26 years<br>Practitioner 3: 25 years	Meditation vs resting with closed eyes	Spectral (FFT)<br>Coherence	Dominant frontal alpha-1 EEG during eyes-closed rest<br><br>Increase in Fronto parietal alpha coherence in meditation
Slagter HA, Lutz A, Greischar LL, Nieuwenhuis S, Davidson RJ	Theta phase synchrony and conscious target perception: impact of intensive mental training	2009	Vipassana	CDM-OM	Eyes closed	64	NA	17 (7 males) meditators<br>23 (9 males) control	3 months retriet	Attentional Blink	ERP (visual)<br>Synchrony (PLV)	Increase in theta phase-locking during target detection and lower P3b amplitude after retreat

Autors	Title	Year	Meditation	Meditation category (ADM,NDM,CDM)	Eyes open or closed?	Number electrodes	EEG reference	Number participants	Experience	Design	Method	Result
Travis F, Haaga DA, Hagelin J, Tanner M, Nidich S, Gaylord-King C, Grosswald S, Rainforth M, Schneider RH	Effects of Transcendental Meditation practice on brain functioning and stress reactivity in college students	2009	TM	NDM	Eyes closed	32	Right/left earlobes average-ear	50 (13 males)	10 weeks	Pre-post test	ERP (CNV)<br>Coherence<br>Spectral (FFT)	No P300 diference<br>No significant Alpha/Gamma ratio
Huang HY, Lo PC	EEG dynamics of experienced Zen meditation practitioners probed by complexity index and spectral measure	2009	Zen	CDM- FA	Eyes closed	30	Linked mastoids	23 pratitioners (6 males)<br>23 control (15 males)	2-12 (mean 8.4) years	meditation vs rest (meditators) relaxation	Non-linear (Averaged complexity index)	Increase in frontal alpha and occipital beta<br>Increased Averaged complexity index, correlated to beta activity
Tang YY, Ma Y, Fan Y, Feng H, Wang J, Feng S, Lu Q, Hu B, Lin Y, Li J, Zhang Y, Wang Y, Zhou L, Fan M	Central and autonomic nervous system interaction is altered by short-term meditation	2009	Integrative body–mind training (IBMT)	CDM	Eyes closed	19	Average reference	86 (42 males)<br><br>Assigned in two groups (experimental and control)<br><br>Only 40 recorded EEG and other physiological measures. The remaining collected SPECT<br><br>34 subjects (17/group) had usable EEG data	5 days of training, 20 minutes a day	Rest before, during and after 5 days of training<br><br>Control group performed relaxation	Multimodal (SPECT- acquired in distinct group)<br>Multimodal<br>Spectral (FFT)	Imaging data demonstrated stronger subgenual and adjacent ventral anterior cingulate cortex (ACC) activity in the meditation group.<br><br>Frontal midline ACC theta was correlated with high-frequency HRV, suggesting control by the ACC over parasympathetic activity.
Tei S, Faber PL, Lehmann D, Tsujiuchi T, Kumano H, Pascual-Marqui RD, Gianotti LR, Kochi K	Meditators and non-meditators: EEG source imaging during resting	2009	Qigong	CDM-FA/OM	Eyes open/ Eyes closed	19	Left ear	8 meditators (3 males)<br>9 control (3 males)	3-30 (mean 11.5)	Comparison between resting state of both groups	Source (Loreta)	Meditators had significantly stronger delta activity in prefrontal cortex (BAs 9, 10, 11, 44, 45, 46, and 47) and anterior cingulate cortex (BA 32).<br>Meditators had significantly weaker delta activity in motor and somatosensory association cortices (BAs 4, 6, and 7), visual association cortex (BAs 18 and 19), left temporo-parietal junction (BA 22, 39, 40), left precuneus (BA 31), and bilateral fusiform gyrus and right parahippocampal gyrus (BA 30).
Subramanya P, Telles S	Changes in midlatency auditory evoked potentials following two yoga-based relaxation techniques	2009	Cyclic meditation	CDM-FA	Eyes closed	1 (Cz)	Linked earlobes	47 males	6-48 months	Response after practice vs Response after rest in supine position	EP (auditory)	Midlatency<br>Following practice compared to before:<br>Increase in Pa and Nb waves peak latencies<br>Increase peak amplitude of Nb wave<br><br>After rest in supine compared to before:<br>Increase in the peak latency of the Na wave
Lutz A, Slagter HA, Rawlings NB, Francis AD, Greischar LL, Davidson RJ	Mental training enhances attentional stability: neural and behavioral evidence	2009	Vipassana<br>Some training in Metta	CDM- FA/OM<br>ADM	NA	64	Average of mastoids	17 (7 males)<br>23 controls (9 males)	2967+/-3162h	baseline -> meditation-><br><br>Attention blink task<br>Dichotic listening task (fucused attention version): attend to tones presented in one ear<br>Dichotic listening task (open attention version): mark deviant tones in either ears<br><br>Tasks performed before and after a 3 month retreat	ERP (Auditory and visual)<br>Synchrony (PLV)	Theta band: Phase consistency increase only in response to the target stimulus, that correlated with less variability in the response of the participant.<br><br>Intensified ERP phase consistency in response to any deviant tone within a broad frequency band (1-30 Hz). No changes in ERP amplitude.<br><br>Amplitude differences were noticeable within ERD in the beta band
Lagopoulos J, Xu J, Rasmussen I, Vik A, Malhi GS, Eliassen CF, Arntsen IE, Saether JG, Hollup S, Holen A, Davanger S, Ellingsen Ø	Increased theta and alpha EEG activity during nondirective meditation	2009	Acem meditation	NDM	Eyes closed	20	Linked Mastoid	18 (13 males)	9-14 years	Meditation Vs resting	Spectral (FFT)	Increase in theta and alpha power across all brain regions
Cahn BR, Delorme A, Polich J	Occipital gamma activation during Vipassana meditation	2010	Vipassana	CDM- FA/OM	Eyes closed	19	Linked ear lobes	16 (11 males)	2.5-40 years (mean 20)	Meditation vs neutral thinking state	Spectral (FFT)	Spectral power: delta (decrease in bilateral frontal regions), Theta increase (Fz - midline) gamma (increase in parieto-occipital regions).

Autors	Title	Year	Meditation	Meditation category (ADM,NDM,CDM)	Eyes open or closed?	Number electrodes	EEG reference	Number participants	Experience	Design	Method	Result
Baijal S, Srinivasan N	Theta activity and meditative states: spectral changes during concentrative meditation	2010	Sahaj Samadhi	CDM- FA	Eyes Closed	64		20 meditators<br>10 control	3-7 years	Baseline (eyes closed and open): meditators vs non-meditators meditation vs relaxation (eyes closed in both)	Spectral (FFT) Coherence	Spectral data in 16 cortical zones<br>In meditation separated by 3 blocks: beggining, middle and end<br><br>Comparing resting:<br>Porcentage: Higher theta in meditators both sides in frontal central sites and right frontal. Lower theta in meditators in left posterior-occipital. And differences in delta and alpha for main effects.<br>Absolute power: Lower alpha in meditators<br><br>Comparing meditation vs relaxation:<br>Increase in theta during deep meditation (middle block) in frontal areas<br>Higher coherence in theta for frontal areas in meditators during meditation
Kumar S, Nagendra H, Naveen K, Manjunath N, Telles S	Brainstem auditory-evoked potentials in two meditative mental states	2010	dharana (focusing on the symbol "OM") and dhyana (effortless single-thought "OM")	CDM- FA/OM	Eyes closed	1 (Cz)	Linked ear lobes	30 males	30 min per day, 4 days in a week, for a minimum of 1 year	Two meditation and two control sessions.<br>Control sessions: (i) ekagrata, i.e. single-topic lecture on meditation and (ii) cancalata, i.e. non-targeted thinking.<br><br>Meditation sessions: (i) dharana, i.e. focusing on the symbol "OM" and (ii) dhyana, i.e. effortless single-thought state "OM".	EP (auditory)	Ep: Analyzed wave V<br><br>The present results showed that the wave V peak latency significantly increased in cancalata, ekagrata and dharana, but no change occurred during the dhyana session
Travis F, Haaga DA, Hagelin J, Tanner M, Arenander A, Nidich S, Gaylord-King C, Grosswald S, Rainforth M, Schneider RH	A self-referential default brain state: patterns of coherence, power, and eLORETA sources during eyes-closed rest and Transcendental Meditation practice	2010	TM	NDM	Eyes closed	32	Linked ears	Experimental group: 19 (5 males)<br>Control group: 19 (4 males)	Novices<br><br>Experimental group received instructions and were expected to meditate for 20 min a day for 10 weeks.	Randomly divided in two groups: Meditation and Control<br><br>Both had baseline recordings.	Spectral (FFT) Coherence Source (eLoreta)	Spectral: higher alpha 1 frontal, lower beta 1 and gamma frontal and parietal in meditation<br><br>Coherence: higher frontal and parietal alpha 1 coherence; higher frontal and frontal-central interhemispheric coherence<br><br>eLORETA: Control group compared to Meditation led to greater alpha1 activity in anterior BA33, dorsal (BA24) and posterior (BA30) cortices, in precuneus (BA31) and in left lingual gyrus and parahippocampus (BA18 e 19). Greater beta 2 activity in right lingual gyrus (BA18 e 19)
Yu X, Fumoto M, Nakatani Y, Sekiyama T, Kikuchi H, Seki Y, Sato-Suzuki I, Arita H.	Activation of the anterior prefrontal cortex and serotonergic system is associated with improvements in mood and EEG changes induced by Zen meditation practice in novices	2011	Zen (Tandem)	CDM-FA	Eyes open	3	A1 and A2	15 (14 males)	Novices	Baseline -> Meditation -> Rest	Multimodal (NIRS) Spectral (FFT)	Increased alpha activity and decreased theta activity during and after meditation.<br><br>NIRS pointed to a marked increase in oxyHb levels in the anterior PFC (BA10 and 9).
Lakey CE, Berry DR, Sellers EW	Manipulating attention via mindfulness induction improves P300-based brain-computer interface performance	2011	Mindfulness meditation induction	CDM- FA	Eyes closed	16	left mastoid	18	naive	9 subjects did a short meditative mindfulness induction (6min) and 9 were in a non-meditative group. Oddball	ERP (visual)	Larger p300 amplitudes in meditators than in control at Cz and PO7<br>Meditation group reach minimun accuracy fasten than control group
Lavallee CF, Hunter MD, Persinger MA	Intracerebral source generators characterizing concentrative meditation	2011	Concentrative meditation	CDM- FA	Eyes closed	19	Ears	7 meditators (3 males)<br>7 control (4 males)	Average: 4.17 years	Meditation or relaxation post meditation baseline	Source (sLoreta)	baseline vs meditation:<br>increase beta1 source at BA33<br>increase beta3 source at BA 31<br>decrease gamma at BA40<br><br>meditation vs relaxation:<br>decrease Beta1 at BA10<br>decrease Gamma at BA11 and BA45<br><br>meditation vs relaxation (controled for baseline):<br>increase Beta2 at BA34 and BA 20<br>increase Beta1 at BA20<br>increase Beta3 at BA21
Sobolewski A, Holt E, Kublik E, Wróbel A	Impact of meditation on emotional processing--a visual ERP study	2011	Budhist practices	CDM-OM	NA	8 (Fp1, F3, F4, Fz, Cz, Pz, O1, O2)	Ear lobes	13 meditators (7 males)<br>13 controls (5 males)	5 years with minimum 5h/week	Neutral, Positive and Negative pictures of IAPS were presented	ERP (visual)	Differences were observed in the late positive potential (LPP). LPP amplitude is typically greater in ERPs evoked by emotionally arousing scenes, specifically negative images, compared to neutral scenes.<br><br>This effect replicatedy, but not in case of meditators' frontal scalp regions, who differed significantly in this respect from control subjects.
Travis F	Comparison of coherence, amplitude, and eLORETA patterns during Transcendental Meditation and TM-Sidhi practice	2011	TM	NDM	Eyes Closed	32	Ears lobes	26 (12 males)	average 25.6 year TM and 19.4 years TM Sidhi	TM group: 2 sessions of TM<br>TM Sidhi group: 1 section of TM followed by another of TM Sidhi	Spectral (FFT) Coherence Source (eLoreta)	Spectral Analysis in grouped frontal and parietal (7 electrodes each group): Interaction in frontal alpha1 and frontal beta1 amplitudes, but no significant differences between groups.<br>Coherence: No interaction<br>eLoreta: higher source for TM-Sidhi for alpha1 at BA19, BA18, BA 37, BA20 and BA21
Radin DI, Vieten C, Michel L, Delorme A	Electrocortical activity prior to unpredictable stimuli in meditators and nonmeditators	2011	Nondual meditation	NDM	Eyes closed	32	vertex	8 meditators<br>8 controls	minimum of 3000 hours	2 tasks (Each one doing during meditation or not), with 5 min rest between each run<br>1) Participants can hear a sound, see a flash, both or nothing and press a button to begin<br>2) Participants can hear a sound or see a flash, pressing the button at sound stimulus	ERP (auditory and visual)	Post-Stimulus shown different patterns for audio and light stimuli in both groups<br><br>Prestimulus: no difference between audio and flash in non meditators;<br><br>In 5 electrodes there was a difference prior to light versus audio stimuli, mostly over occipital region, with main difference occuring at the free-running task.

Autors	Title	Year	Meditation	Meditation category (ADM,NDM,CDM)	Eyes open or closed?	Number electrodes	EEG reference	Number participants	Experience	Design	Method	Result
Ren J, Huang Z, Luo J, Wei G, Ying X, Ding Z, Wu Y, Luo F	Meditation promotes insightful problem-solving by keeping people in a mindful and alert conscious state	2011	Susoku	CDM-FA	"Slightly closed"	2 (bilateral prefrontal cortex)	A1 and A2	48 (23 males) in 3 groups of 16 each	Naive	Pre-test: 10 insight problems<br>Test: Meditation (counting breaths - 10 or 100) or Auditory judgment task<br>Post-test: insight problems not solve at pre-test	Spectral (Wavelets)	A one-way within-groups ANOVA showed a significant difference in the percentage of alpha waves among the three groups.
Hinterberger T, Kamei T, Walach H	Psychophysiological classification and staging of mental states during meditative practice	2011	Zen, Qi-Gong, Sahaja Yoga, Western contemplative methods, and spiritist or mediumistic	CDM-FA<br>NDM	Differs acording to meditation practice	62	shoulder	49	31 > 1000h<br>11 <40	1-baseline (eyes open, eyes closed, reading book or computer screen)<br>2- meditation session<br>3- guided meditation (4 different meditations)	Linear classifier	Classification among 7 possible states (3 resting and 4 guided meditation)<br><br>About 83% of epochs correctly classified. The personal meditation of each meditator was not included.
Moore A, Gruber T, Derose J, Malinowski P	Regular, brief mindfulness meditation practice improves electrophysiological markers of attentional control	2012	Mindfulness	CDM-FA	Eyes Open	64	Average reference	Meditation Group: 12<br>Control Group: 16	All started meditation naive. Meditation group received instruction and was advised to practice for at least 10 minutes a day for 16 weeks	Stroop Task performed at beggining after a 2 hour mindfulness training (T1), after 8-10 weeks (T2) and at the end (T3)	ERP (Stroop)<br>Source (VARETA)	Meditators had increase of lateral posterior N2 amplitudes over both hemispheres, irrespective of stimulus congruency.<br><br>Neural sources (VARETA) of N2: increased activity in the left medial and lateral occipitotemporal areas for congruent stimuli, contrasted by decreased activity in similar brain areas in the control group.<br><br>Changes of the P3 for incongruent stimuli. Control group exhibited an increase of the P3 amplitude and a decrease was observed for the meditation group. The P3 decrease in electrode space was accompanied by significantly decreased signal strength in source space, which comprised lateral occipitotemporal and inferior temporal regions of the right hemisphere.
Lehmann D, Faber PL, Tei S, Pascual-Marqui RD, Milz P, Kochi K	Reduced functional connectivity between cortical sources in five meditation traditions detected with lagged coherence using EEG tomography	2012	Tibetan Buddhists<br>QiGong<br>Sahaja Yoga<br>Ananda Marga<br>Zen	NDM	Eyes closed/ half closed	19	NA	Tibetan Buddhists (13)<br>QiGong (15)<br>Sahaja Yoga (14)<br>Ananda Marga (14)<br>Zen (15)	Tibetan Buddhists (1-25; mean 12.2 years)<br>QiGong (2-13; mean 6.6 years)<br>Sahaja Yoga (1-20; mean 8.5 years)<br>Ananda Marga (5-33; mean 16.9 years)<br>Zen (5-21; mean 12.3 years)	Initial Resting<br>Meditation<br>Final resting	Spectral (FFT)<br>Coherence (Loreta-based lagged/Classical coherence)	Lagged corehence was always lower in meditation than in initial or final rest. Classical coherence also show predominantly lower coherence in meditation.<br>Power spectra among the 5 groups differed among them.
van Leeuwen S, Singer W, Melloni L	Meditation increases the depth of information processing and improves the allocation of attention in space	2012	Budhist meditation	CDM- FA/OM	Eyes open	129	Vertex	Cross-sectional EEG study.:<br>8 monks (5 males)<br>8 novices (5 males)<br><br>Longitudianl study:<br>6 meditators (2 males)<br>6 controls (2 males)	Cross-sectional EEG study.:<br>mean 5 years<br><br>Longitudianl study (4-day retreat):<br>mean 3 years	Navon<br>A large number (global) formed by small numbers (local). With 1 and 2 as targets and 3 and 4 as distarctors	ERP (Visual)<br>Source (MSP)	Differences in longitudinal was provided in the RT, for EEG data only says about meditators vs control group.<br><br>ERP:<br>P1 amplitude was higher for local than global at left hemisphere in control group and in both hemispheres for meditation group.<br>N1 amplitude was higher in global than local at right hemisphere for meditators, but not found hierarchical selection in both hemispheres of control group.<br>N2 amplitude was higher for global than local in right hemisphere and opposite in lef hemisphere. In control group no hierarchical processing found in either hemisphere.<br>P3 was higher for global than local in both groups.<br><br>Source<br>250-400ms<br>bilateral clusters in temporal, occipital, and frontal regions, corresponding to the inferior temporal gyrus, inferior occipital/fusiform gyrus and DLPFC activation in the meditation group but not in the control group
Ravnik-Glavač M, Hrašovec S, Bon J, Dreo J, Glavač D.	Genome-wide expression changes in a higher state of consciousness	2012	First practitioner: Zen and Kundalini meditation<br>Second practitioner: mental quietness and visualization of Buddha	CDM- FA	Meditator 1: Eyes open<br>Meditator 2: Eyes closed	128	Average reference	2	1: 23 years<br>2: 25 years	EEG was recorded during rest and meditation	Genome<br>Spectral (Complex Demodulation)	Meditator 1: Power increased in theta and alpha in continuous manner during mediation, mainly in parieto-occipital and frontal or fronto-central regions. Power changes in alpha 1 (8–10 Hz) were most consistent with the time course of meditation<br><br>Meditator 2: Power increased in theta and alpha in continuous manner during mediation, mainly in occipital regions. Power changes in alpha 1 were consistent with the time course of meditation.

Autors	Title	Year	Meditation	Meditation category (ADM,NDM,CDM)	Eyes open or closed?	Number electrodes	EEG reference	Number participants	Experience	Design	Method	Result
Telles S, Raghavendra BR, Naveen KV, Manjunath NK, Subramanya P	Mid-latency auditory evoked potentials in 2 meditative states	2012	meditative focusing and meditation based on specific instructions. Participants with prior experience with Sanskrit syllable OM	CDM	Eyes closed	4 (Cz, FPz and earlobes)	Earlobes	60	Minimum of 6 months mean (20.95 +/- 14.21 months)	Components of the Mid-latency auditory evoked potentials (MLAEPs) measured in 4 different mental states:<br>1- meditative focusing<br>2- meditative de-focusing or effortless meditation (meditative)<br>3- random thinking<br>4- nonmeditative focusing (nonmeditative) sessions.<br><br>The participants were randomly assigned to the meditive or nonmeditative sessions. Each session was divided into: pre sessions, session and post session.	EP (Auditory)	Analyzed peak latency and amplitude of the MLAEPs components (Na, Pa, Nb) compared pre, during and post portion of one session<br><br>Peak latency increase in Na and Pa during meditation and Nb on nonmeditation comparing post-session vs pre-session.<br><br>Peak amplitude decrease in Pa during meditative and nonmeditative sessions compared to respective pre-sessions
Saggar M, King BG, Zanesco AP, Maclean KA, Aichele SR, Jacobs TL, Bridwell DA, Shaver PR, Rosenberg EL, Sahdra BK, Ferrer E, Tang AC, Mangun GR, Wallace BA, Miikkulainen R, Saron CD	Intensive training induces longitudinal changes in meditation state-related EEG oscillatory activity	2012	Focused attention meditation techniques	CDM-FA	Eyes closed	88-channel equidistant montage.	Average of channels	22: initial retreat (10 males)<br>22: control group (11 males)	Retreat: M = 2855.6 h, SD = 2994.1<br>Control: M = 2272.7 h, SD = 2326.3	Acquisition during practice at three assessment points during the retreat:<br>- beggining (pre)<br>- Middle (mid)<br>- End (post)	Spectral (IAF)	Replicable and robust reductions in beta-band power over central-parietal regions and decreased IAF following three months of intensive FA meditation.
Goshvarpour A, Goshvarpour A, Rahati S, Saadatian V	Bispectrum estimation of electroencephalogram signals during meditation	2012	Sit quietly focusing on the breath	CDM-FA	Eyes closed	3 (Fz, Cz, Pz)	Linked ear lobes	Guided:11 experienced, 4 novices<br>Unguided: 10 experienced<br><br>all females	Guided:experienced (5 to 7 years)<br>Unguided: mean 7 years	Before and during meditation in guided and unguided practice	Synchrony (Bispectrum estimation)	The maximum value of the magnitudes of bispectrum (power) is increased during<br>meditation. The mean Bispectrum<br>magnitude of each channel is increased during<br>meditation.<br><br>Increments of phase coupling are more obvious in occipital region (Pz) than frontal and central regions (Fz and Cz). Besides that phase-coupled harmonics are shifted to the higher frequencies during meditation.
Park YJ, Park YB	Clinical utility of paced breathing as a concentration meditation practice	2012	Paced breathing (PB)	CDM	Eyes closed	6 (F3, F4, T3, T4, P3, P4)	NA	58	naive participants	The experiment consisted answering a short version of the Temperament Character Inventory (TCI-RS) followed by the session: resting and successive spontaneous breathing; vs paced breathing (following auditory cues)<br><br>The authors summed the score of each subscale for the TCI-RS to examine which subscales among the seven subscales were indicative ofthe percent changes in heart rate variability (HRV) and EEG parameters during PB.	Spectral (FFT)	Increased alpha power and local decreases in theta power during PB.<br><br>Authors also suggests that personality traits mediate neurophysiological changes more prominently during PB than do autonomic changes.
Ahani A, Wahbeh H, Miller M, Nezamfar H, Erdogmus D, Oken B	Change in physiological signals during mindfulness meditation	2013	Mindfulness meditation	CDM-FA	Eyes closed	32	NA	34	naive	6 week intervention<br>Listening 15 min podcast eyes closed<br>Meditation 15 min	Spectral (PSD)<br>Classification(SVM)	Spectral<br>Increase in alpha, beta and theta during meditation<br><br>SVM<br>70% accuracy using EEG<br>80% using EEG+respiration
Teper R, Inzlicht M	Meditation, mindfulness and executive control: the importance of emotional acceptance and brain-based performance monitoring	2013	various meditation backgrounds (i.e. Vipassana, Shambhala, concentrative , etc).	CDM	NA	32	Average ears	20 meditators (9 males)<br>18 non-meditators (2 males)	at least one year (mean 3.19)	Stroop task<br><br>Analysis of error-related negativity (ERN), a neurophysiological response that occurs within 100 ms of error commission in FCz while the error positivity (Pe) was defined as the maximum peak between 150 and 250 ms postkey press at FCz electrode.	ERP (Stroop)	Meditators showed a higher ERN and had fewer errors.<br><br>Meditators do not exhibit stronger Pe's.
Cahn BR, Delorme A, Polich J	Event-related delta, theta, alpha and gamma correlates to auditory oddball processing during Vipassana meditation	2013	Vipassana	CDM- FA/OM	Eyes closed	19	Linked ear lobes	16 (11 males)	2.5-40 years (M=20, SD=12.1)	Meditation vs mind wandering<br><br>Auditory oddball	ERP (Auditory)<br>Spectral (Wavelets-Morlet)<br>Coherence (complex Gaussian wavelet)	Decreased evoked delta power to distracter stimuli concomitantly with a greater ER reduction of late (500–900 ms) alpha-1 (8–10 Hz) activity<br><br>Standard stimuli : increased early ER alpha phase synchrony (inter-trial coherence) and evoked theta phase synchrony<br><br>During meditation: greater differential early-evoked gamma power to the different stimulus classes. Correlation analysis indicated that this effect stemmed from a meditation state-related increase in early distracter-evoked gamma power and phase synchrony specific to longer-term expert practitioners.

Autors	Title	Year	Meditation	Meditation category (ADM,NDM,CDM)	Eyes open or closed?	Number electrodes	EEG reference	Number participants	Experience	Design	Method	Result
Tsai JF, Jou SH, Cho W, Lin CM	Electroencephalography when meditation advances: a case-based time-series analysis	2013	Breath meditation disappearance of self-boundaries, blissful feeling, and complete quiescence during meditation.	CDM/FA<br>NDM	Eyes closed	3 (Fp1, Fp2, Oz)	Left activity: Fp1-Oz<br>Right activity: Fp2-Oz	1	2 years	Rest X ordinary meditation X advanced meditation	Spectral (FFT)	Meditation X baseline: higher activities compared with baseline were found only in bilateral theta,but not in alpha bands.<br>Advanced meditation X breath meditation: increased activities were found both in bilateral alpha and theta bands compared against breath meditation.<br>Advanced meditation X baseline:bilateral alpha and theta bands revealed significantly increased activities.
Hagerty MR, Isaacs J, Brasington L, Shupe L, Fetz EE, Cramer SC	Case study of ecstatic meditation: fMRI and EEG evidence of self-stimulating a reward system	2013	Ecstatic meditations - jhanas	CDM-FA	NA	256	Vertex (Cz)	1	17 years	Recordings of each sequential jhana state, marqued by the participant	Multimodal (fMRI- not simultaneous)<br>Spectral (FFT)	EEG signal shifted to the lower-power bands of theta and alpha1<br>The fMRI show changes in brain activity in 11 regions shown to be associated with the subjective reports, and these changes occur promptly after jhana is entered.
Moynihan JA, Chapman BP, Klorman R, Krasner MS, Duberstein PR, Brown KW, Talbot NL	Mindfulness-based stress reduction for older adults: effects on executive function, frontal alpha asymmetry and immune function	2013	MBSR	CDM	Eyes open and closed	19 (FP1/2, F3/4, F7/8, C3/4, T7/8, P7/8, P3/4, O1/2, Fz, Cz, Pz)	Digitally derived linked-mastoids	110	naive	Participants were randomly divided into two groups: MBSR and wait list<br>EEG recorded prior to the start of the program, 8 weeks later just after program completion and also 32 weeks after the first recording.	Spectral (FFT,α asymmetry)	Leftward alpha asymmetry at F3/4 was reported to be significantly greater in the MBSR group when compared to waiting list immediatly after the completion of the MBSR programe. MBSR participants exhibit a relatively constant alpha asymmetry and waiting list participants a significant rightward shift in alpha asymmetry
Delgado-Pastor LC, Perakakis P, Subramanya P, Telles S, Vila J	Mindfulness (Vipassana) meditation: effects on P3b event-related potential and heart rate variability	2013	Vipassana	CDM- FA/OM	Eyes closed	3	interconnected earlobes	10 (all male)	Min 2 years<br>Average 7.5	5 min resting<br>pre-intervention oddball task<br>30 min meditation/random thinkin<br>Post-intervention oddball	ERP (auditory)	Larger P300 after meditation than before meditation and after random thinking.
Berkovich-Ohana A, Glicksohn J, Goldstein A	Studying the default mode and its mindfulness-induced changes using EEG functional connectivity	2013	Theravada	CDM- FA/OM	Eyes closed	64	Cz	48 (3 meditation group with 12 participants each + control group)	Short ~900h (12 participants)<br>Intermediate ~2570h (12 participants)<br>Long-term ~7500h (12 participants)	Resting state, a time producing task and meditation	Synchrony (Mean phase coherence - MPC)	Increased alpha MPC in left hemisphere
Kim DK, Lee KM, Kim J, Whang MC, Kang SW	Dynamic correlations between heart and brain rhythm during Autogenic meditation	2013	Autogenic meditation	CDM-FA	Eyes closed	19	Linked ear lobes	13 (6 males)	Completed 8 weeks autogenic meditation course and have daily meditated for at least 1 month (Mean ± SD = 0.8 ± 0.5 years, range = 0.1–2.0 years)	Baseline vs meditation	Spectral (FFT)<br>Coherence (Lagged Coherence)<br>Multimodal	Alpha power averaged over all channels increased during meditation<br>Alpha band coherence averaged over all channels increased during meditation
Tan LF, Dienes Z, Jansari A, Goh SY	Effect of mindfulness meditation on brain-computer interface performance	2014	Mindfulness	CDM - FA	Eyes-closed	64	NA	76<br>20 meditation training<br>20 music training	12 weeks	Meditation learning group<br>Guitar learning group<br>Control group	BCI	Meditation group has a higher accuracy than guitar learning group and control group
Xue SW, Tang YY, Tang R, Posner MI	Short-term meditation induces changes in brain resting EEG theta networks	2014	Integrative Body-Mind Training	CDM - FA	Eyes-closed	28	FCz	45 (29 males)	24 Meditation Group<br>21 Relaxation group	1 week of meditation/relaxation training (30 min; 3.5h in total)	Graph Theory (Funcional connectivity by synchronization likelihood)	Decreased average path lenght and increased clustering coeficient in Meditation group after training, not shown in relaxation group.
Hinterberger T, Schmidt S, Kamei T, Walach H	Decreased electrophysiological activity represents the conscious state of emptiness in meditation	2014	different kinds of spiritual traditions such as Zen-Buddhism (11), Qi-Gong (4), Tibetan Buddhism (4), Sahaja Yoga (8), Western contemplative methods (7), spiritists or mediumistic practice (5), or were spiritual healers or shamans (3).	CDM- FA/OM	Eyes closed/open	64	Common average	Meditation specific states: 30 (19 males)<br>Influence of meditation experience: 50	Meditation specific states: more than 5 year or 1000 hours (mean 6498 h)<br>Influence of meditation experience 3 equal sized subgroups: For the between-group analyses, the group of 17 most experienced meditators (MEM) was compared to the group of 17 least experienced meditators (LEM), the intermediate group was omitted. The 17 LEM (13 males) had less than 500 h of meditation practice (mean practice of 111 h and 3.4 years); the 17 MEM (12 males,) had at least 3800 h of meditation practice (mean practice of 9716 h and 27 years).	Baseline session: eyes opened, eyes closed, silently reading a text from a book or a computer screen.<br>Meditation session: meditate in their own usual way (idiosyncratic). Controls received instructions to focus on the breath<br>Guided meditation session: Presence/Monitoring, Thoughtless emptiness (TE), Focused attention,Spatial connectedness	Multimodal<br>Spectral (IAF)	30 experienced meditators:<br>Normal resting is associated with increased delta and theta waves compared to meditative states (Idiosyncratic meditation, presence/monitoring, and TE). Furthermore, compared to resting, presence/monitoring had an increased alpha activity and beta 1 a lower activity in Idiosyncratic meditation and TE<br>MEM vs LEM:<br>IAF difference between groups small and not significant<br>Only MEM showed significant delta and theta power during idiosyncratic meditation compared to resting. The MEM also have decreased power in beta1
Kim DK, Rhee JH, Kang SW	Reorganization of the brain and heart rhythm during autogenic meditation	2014	Autogenic meditation	CDM-FA	Eyes closed	19	Earlobes	14	Completed 8 weeks autogenic meditation course and have daily meditated for at least 1 month (Mean ± SD = 0.8 ± 0.5 years, range = 0.1–2.0 years)	Baseline followed by flexible time of meditation all with eyes closed.<br>Compare baseline vs meditation	Spectral (FFT)<br>Coherence	EEG Power: Significant decreases in the delta and near significant decreases in the high beta band absolute power during meditation compared to the baseline<br>EEG Coherence: Significant increases in the alpha, beta, high beta, gamma, and the high gamma band coherence averaged over 171 channel combinations during meditation compared to the baseline.

Autors	Title	Year	Meditation	Meditation category (ADM,NDM,CDM)	Eyes open or closed?	Number electrodes	EEG reference	Number participants	Experience	Design	Method	Result
Ahani A, Wahbeh H, Nezamfar H, Miller M, Erdogmus D, Oken B	Quantitative change of EEG and respiration signals during mindfulness meditation	2014	Mindfulness	CDM - FA	Eyes closed	32	Cz	34 (6 males)	6-week	Listening podcast (Eyes-closed)<br>Guided meditation (Eyes-closed)	Power Spectral Density<br>Synchrony (PLV)<br>Classification (Suport Vector Machine)	PSD:<br>Increase in all regions for Beta and theta only not at frontal region. Increase in Occipital and Right temporal alpha.<br><br>PLV:<br>Increase in number of pairs of electrodes with higher PLV than treshold in Theta, Alpha and Beta<br><br>SVM:<br>EEG only was able to differentiate between meditation and non-meditation, however with addition of respiratory feature was even better.
Milz P, Faber PL, Lehmann D, Kochi K, Pascual-Marqui RD	sLORETA intracortical lagged coherence during breath counting in meditation-naïve participants	2014	Breath counting	CDM - FA	Eyes closed	58	Cz	23 (all males)	Naive	3 Resting vs 2 breath counting	Spectral (FFT)<br>Source (sLoreta)<br>Coherence in Loreta sources	FFT: Only alpha1 at F1 was higher in meditation than resting.<br>Loreta Source: No statistical diference after correction for multiple tests.<br>Loreta Coherence: Lower lagged coherence with left BA10 and right BAs 3, 10, 17, 40.<br>Conventional coherence: Higher coherence at 17 pairs and lower at 7 pairs.
Kopal J, Vyšata O, Burian J, Schätz M, Procházka A, Vališ M	Complex continuous wavelet coherence for EEG microstates detection in insight and calm meditation	2014	Vipassana	CDM- FA/OM	NA	19	linked earlobes	meditors 7 (all males)<br>control 7 (6 males)	> 1000h	Calm meditation (30 min)<br>Insight meditation (30 min)	Coherence (Complex continuous wavelet -Morlet)	Real parts of wavelet coherence in frontal regions and imaginary part in frontal-occipital pairs of electrodes shown higher accuracy for differentiate between groups.
Jo HG, Wittmann M, Hinterberger T, Schmidt S	The readiness potential reflects intentional binding	2014	Mindfulness	CDM	Measurement not performed during meditation	64	linked mastoids	20 meditators<br>20 matched controls	Medidators had least 3 years of regular mindfulness meditation	Intentional binding paradigm (attentional paradigm).<br>Interested in the behavior of meditators vs non meditators	ERP (readiness potential)	No significant difference between meditators and control in behavioral and EEG responses.
Thomas J, Jamieson G, Cohen M	Low and then high frequency oscillations of distinct right cortical networks are progressively enhanced by medium and long term Satyananda Yoga meditation practice	2014	Satyananda Yoga meditation	NDM	Eyes closed	25	Left mastoid	12	6 intermediate practitioners (mean 4 years)<br>6 experienced practitioners (mean 30 years)	The EEG was recorded during the following conditions: body-steadiness meditation, mantra meditation, and non-meditation mental calculation condition.	Source analysis (eLoreta)	Intermediate meditators exhibit greater activity particularly in alpha1 and theta during mental calculation, body-stediness and mantra meditation. The number of significant voxels was double in these conditions; These differences were greatest in right (R) superior frontal and R precentral gyri and extended back to include the R parietal and occipital lobes.<br><br>Advanced Yoga meditators showed greater activity in high frequencies (beta and especially gamma) in all conditions but greatly expanded during meditation practice.<br><br>Across all conditions (meditation and non-meditation) differences were greatest in the same regions: R insula, R inferior frontal gyrus and R anterior temporal lobe. Distinct R core networks were identified in alpha1 (8–10 Hz) and gamma (25–42 Hz) bands, respectively. The voxels recruited to these networks greatly expanded during meditation practice to include homologous regions of the left hemisphere.
Reva NV, Pavlov SV, Loktev KV, Korenyok VV, Aftanas LI	Influence of long-term Sahaja Yoga meditation practice on emotional processing in the brain: An ERP study	2014	Sahaja Yoga meditation	CDM	Recording was not during meditation	recorded 62 used 25	NA	40 (all males)	20 experienced (mean = 11.45 years, SD = 4.35)<br>20 naive controls	Participants were required to do 7 min resting period and then look at a sequence of facial expressions randomly assigned as positive, negative or neutral. Each picture was shown for 5s.	ERP (Visual)	The meditators N170, P200 and P300 were attenuated for both positive and negative pictures and this effect was more prominent over the right hemisphere.<br><br>No differences found in LPP associated with meditation practices<br><br>Stronger ERP negativity for N200 and P300(P3a) for meditators compared to the controls, regardless of picture valence.
Fan Y, Tang YY, Tang R, Posner MI	Short term integrative meditation improves resting alpha activity and stroop performance	2014	Integrative bodymind training (IBMT)	CDM	Eyes Open	16	FCz	43	naive to meditation before the protocol	Participants were separated into two groups.<br>Group one: 5 days of IBMT<br>Group two: 5 days of relaxation training<br><br>EEG was recorded before and after the 5-day training. Each session started with 5 min eyes-open followed by Stroop task.	Spectral (FFT)	The IBMT group decreased its stroop reaction time (RT) and showed increased resting mean alpha power.<br>The higher the enhancement of resting alpha power, the stronger the improvement of conflict RT.
Tanaka GK, Peressutti C, Teixeira S, Cagy M, Piedade R, Nardi AE, Ribeiro P, Velasques B	Lower trait frontal theta activity in mindfulness meditators	2014	mindfulness	CDM-OM	NA	20	Earlobes	20	10 experienced meditators with at least 5 years of practice (11.61 ± 7.40 years)<br>10 without anz meditation experience	The sessions were composed by:<br>- 4 min resting state<br>- 40 min Mindfulness meditation<br>- 4 min resting state	Spectral (FFT)	Frontal theta (FT) power was statistically significantly greater during the meditation compared to rests 1 and 2 for the F3, F4, F7, and F8 derivations and significantly reduced during rest 2 compared to rest 1 for the Fp1, F7, and F8 derivations).<br><br>In the control group, the FT power was statistically significantly higher during the meditation compared to rest 1 for all of the derivations studied, although it remained unchanged after the meditation (rest 2) for the Fp1 and F8 derivations.
Hawkes TD, Manselle W, Woollacott MH	Tai Chi and meditation-plus-exercise benefit neural substrates of executive function: a cross-sectional, controlled study	2014	Tai Chi	NA	NA	256	referenced to VREF	54	Practitioners were required to have at least 5 years or more of practice for three times/week during 30 minutes/session	4 groups: sedentary, aerobic, tai-chi and meditation<br>Each group performed the Visuo-spatial task switch (VSTS)	ERP (Visual)	The groups comprehended of Tai-Chi and Meditation+exercise participants showed a larger P3b (late P300) amplitude on switch trials compared to sedentary controls.

Autors	Title	Year	Meditation	Meditation category (ADM,NDM,CDM)	Eyes open or closed?	Number electrodes	EEG reference	Number participants	Experience	Design	Method	Result
Dissanayaka C, Ben-Simon E, Gruberger M, Maron-Katz A, Hendler T, Chaparro-Vargas R, Cvetkovic D	Information flow and coherence of EEG during awake, meditation and drowsiness	2014	NA	NA	Eyes closed	30 (only 6 utilized)	between Fz and Cz	10 awake<br>10 drowsy<br>10 meditating	NA	Baseline vs<br>Fall Sleep or meditate or remain awake (Eyes closed)	Coherence (based on Welch's method)<br>Minimum Variance Distortionless Response coherence (MVDR)<br>Direct Transfer Method (DFT)	Diferences in coherence and DTF comparing meditation with other attentive conditions in several frequency bands.
Faber PL, Lehmann D, Gianotti LR, Milz P, Pascual-Marqui RD, Held M, Kochi K	Zazen meditation and no-task resting EEG compared with LORETA intracortical source localization	2015	Zazen	CDM-OM	Eyes open/ eyes closed	58	NA	15 (9 males)	5-21 (average 5.6)	Resting (Eyes Open and Eyes Closed)<br>Zazen (Eyes 2/3 closed)	Source (sLoreta)	In comparison with rest:<br>Increase in alpha1 and alpha2 (right hemisphere frontal areas)<br>Decrease in beta1 and beta2 (bilateral posterior clusters)
Lee YH, Shiah YJ, Chen SC, Wang SF, Young MS, Lin CL	Improved emotional stability in experienced meditators with concentrative meditation based on electroencephalography and heart rate variability	2015	Tibetan Nyingmapa	CDM-FA	Eyes open	6	Earlobes	Experencied: 10 (6 males)<br>Novice: 10 (5 males)	Experencied: mean 20.5 years<br>novice: 1-5 years	Baseline (Eyes open and closed; 4x 60 seconds)<br>5 min meditation (3x with 2 min interval)<br>IAPS pictures with meditation (2x 9 minutes)	Spectral (FFT)<br>Multimodal	Alpha and theta power increased at frontal and central regions; theta increased at posterior sites; decreased gamma and beta when comparing resting with meditation.<br><br>At visual stimuli experencied meditators increased alpha in frontal and central regions and reduced gamma in central region. Novice meditators alpha, beta and gamma decreased in all recorded channels.
Dissanayaka C, Ben-Simon E, Gruberger M, Maron-Katz A, Sharon H, Hendler T, Cvetkovic D	Comparison between human awake, meditation and drowsiness EEG activities based on directed transfer function and MVDR coherence methods	2015	NA	NA	Eyes closed	30 (only 6 utilized)	between Fz and Cz	10 awake<br>10 drowsy<br>10 meditating	at least 1000h	Baseline vs condition	Coherence (based on Welch's method)<br>Minimum Variance Distortionless Response coherence (MVDR)<br>Direct Transfer Method (DFT)	Significant increase in the flow of information (effective connectivity) in delta was found only in the meditation condition, originating from frontal, parietal and occipital regions.
Cosme D, Wiens S	Self-reported trait mindfulness and affective reactivity: a motivational approach using multiple psychophysiological measures	2015	NA	NA	Eyes open	128	CP1	51 (26 male)	7 participants had experience (1-8 years; mean 3.1 years)	51 IAPS figures	Multimodal<br>ERP (Visual)	LLP (400-800ms) amplitudes to pleasant and unpleasant pictures were greater than to neutral pictures. There were no statistically significant main effects of trait mindfulness nor Emotion x Mindfulness interactions.<br>P3 shown no main effect of emotion. There was a main effect of trait mindfulness (FFMQ-NR) on P3 amplitudes, suggesting that P3 amplitudes were generally decreased with higher mindfulness. However, none of the Emotion x Mindfulness interactions was statistically significant.
Jo HG, Hinterberger T, Wittmann M, Schmidt S	Do meditators have higher awareness of their intentions to act?	2015	mindfulness meditation	CDM	NA	64	P9	20 (7 males) meditators<br>20 (7 males) control	3-32 years (Average 10.1 years)	Two Libet-type tasks	RP (readiness potential)	Early RP was different in control group for the two tasks, but not in meditators group. So meditators has a more consistent pattern pf readiness potential (RP)
Pasquini HA, Tanaka GK, Basile LF, Velasques B, Lozano MD, Ribeiro P	Electrophysiological correlates of long-term Soto Zen meditation	2015	Soto zen	CDM-OM	Eyes open	128	NA	17 (11 male) meditators<br>15 (11 male) control group	>2 years (average 1430h)	Active and passive ERP visual	ERP (Visual)	Correlations between the frequency of weekly meditation practice at passive observvation task and:<br>Increased theta and theta/beta induced relative power
Hauswald A, Übelacker T, Leske S, Weisz N	What it means to be Zen: marked modulations of local and interareal synchronization during open monitoring	2015	Zen	CDM-OM	Eyes open	128		11 (5 males)	1 mo - 25 years (Average 12 years)	Meditation vs rest post meditation	Network analysis<br>Source (DICS)	Correlation between 160-170Hz graph measeures with MAAS score.<br>Higher levels of mindfulness with lower small wordedness, global and local clustering in paracentral, insular and thalamic regions during meditation.
Berman AE, Stevens L	EEG manifestations of nondual experiences in meditators	2015	TM, Vipassana or Mindfulness meditation, breath/body awareness, mantra meditation and visualization.	CDM-FA<br>CDM-OM<br>NDM	NA	19	Linked ears	44 (17 males)	0.5-44 years (average 12.64 years)<br><br>up to 1239.94 hours of meditation are considered low experience<br>above 1239.94 hour of meditation are considered high experience	7.5 min pre meditation (eyes closed) baseline<br>30-60 min eyes closed meditation<br>7.5 min post meditation (eyes closed) baseline<br><br>During meditation participants should wink their left eyes when become aware the they have transitioned from a state of non-thought)or tancendent, mental silence, or nonduality), back into a state of thought. Analysis of 30s before wink with 30 after and with hole meditation	Spectral (FFT)	Pre Baseline:<br>Frontal theta and theta2 are higher among participants with higher experience<br>Post baseline:<br>Higher theta2, frontal theta, parietal theta2 and central theta 2 in higher experienced meditators.<br><br>Non duality experience:<br>20 participants shown clear signal of non-duality experiences, in a total of 48 epochs.<br>No difference between 30s before and 30s after winks (moment when participant realize returning from a nondual experience).<br>Higher averal delta, theta and alpha in pre and lower gamma in the 30s before winks comparing with hole meditation period.

Autors	Title	Year	Meditation	Meditation category (ADM,NDM,CDM)	Eyes open or closed?	Number electrodes	EEG reference	Number participants	Experience	Design	Method	Result
Telles S, Deepeshwar S, Naveen KV, Pailoor S	Long Latency Auditory Evoked Potentials during Meditation	2015	Meditative focusing (dharana) and Effortless meditation (dhyana)	CDM-FA<br>CDM-OM	Meditative focusing (Eyes open)<br>Effortless meditation (Eyes closed)	1 (Cz)	Linked ears	48	>3 months<br>20.9 months average	4 different interventions<br>Random Rhinking<br>Nonmeditative Focused Thinking<br>Meditative Focusing<br>Meditative Defocusing or Effortless Meditation<br><br>Recording before, during (4 segments) and after intervention.<br><br>Auditory (click) stimuli	EP(Auditory)	Long latency auditory evoked potential (LLAEP)<br>P1 (40-60ms after click)<br>N1 (80-115ms after click)<br>P2 (140-180ms after click)<br>N2 (200-280ms after click)<br><br>Decrease in latency of the P2 wave during and after meditation<br>P1, P2 and N2 decreased amplitude peak during random thinking and nonmeditative focused thinking.
Saggar M, Zanesco AP, King BG, Bridwell DA, MacLean KA, Aichele SR, Jacobs TL, Wallace BA, Saron CD, Miikkulainen R	Mean-field thalamocortical modeling of longitudinal EEG acquired during intensive meditation training	2015	breath focused FA meditation	CDM-FA	Eyes closed	88-channel equidistant montage.	Average of channels	22: initial retreat (10 males)<br>22: control group (11 males)	Retreat: M = 2855.6 h, SD = 2994.1<br>Control: M = 2272.7 h, SD = 2326.3	Acquisition during practice at three assessment points during the retreat:<br>- beggining (pre)<br>- Middle (mid)<br>- End (post)	Mean-field computational modeling	First, the intrathalamic gain parameter decreased significantly with training<br><br>Second, the corticothalamic delay parameter increased with training, indicating an increase in transmission delay between modeled cortical and thalamic cells.<br><br>Lastly, intra-TRN connectivity analysis implicated a clear anterior–posterior connectivity-based segregation in the modeled TRN layer. While this anterior–posterior connectivity remained stable over training assessments, an increase in between-cluster anterior–posterior connectivity was observed in both retreat groups following training.
Tanaka GK, Maslahati T, Gongora M, Bittencourt J, Lopez LC, Demarzo MM, Budde H, Teixeira S, Basile LF, Campayo JG, Cagy M, Ribeiro P, Velasques B	Effortless Attention as a Biomarker for Experienced Mindfulness Practitioners	2015	Open Monitoring	CDM-OM	NA	20 (only 8 frontals are analysed)	Earlobes	11 experencied (7 males)<br>10 naive (5 males)	at least 5 years (average 12.23 years)	4 min pre baseline<br>40 min meditation (with analysis in the period 30-34min)<br>4 min post baseline	Spectral (FFT)	Lower frontal power beta in experencied group in all analysed epochs
Saunders B, Rodrigo AH, Inzlicht M	Mindful awareness of feelings increases neural performance monitoring	2016	Mindfulness	CDM	N/A	7	Bilateral earlobes	41 participants	N/A (it seems that they were naive)	Participants were randomly assigned to emotion-focused or tought-focused gorup.<br>All participants did a go/no-go experiment pre and post introductory meditation instructions according to the assigned group-	ERP (Visual)	The participants from the emotion focused group had a higher ERN which had no effect on the thought focused group.<br>None of the groups showed alterations in the Pe activity.
Fingelkurts AA, Fingelkurts AA, Kallio-Tamminen T	Long-term meditation training induced changes in the operational synchrony of default mode network modules during a resting state	2016	Kriya Yoga	CDM	Eyes closed	19		10	4 months	Resting state	Syncrony (Alpha DMN Operational synchrony)	Decrease of operational synchrony within the DMN after 4 months of training.
Hinterberger T, von Haugwitz A, Schmidt S	Does a Healing Procedure Referring to Theta Rhythms Also Generate Theta Rhythms in the Brain?	2016	Theta healing	CDM	Eyes open and closed	32	Common reference between 2 persons	10 healers<br>10 clients	Healers were experienced trainers and clients were naive to the technique	4 pre and post resting states (eyes open and eyes closed) and 6 sessions of Theta Healing	Spectral (FFT)	Significand decrease in Theta-2 band in the healers. Small correlation in the amplitudes of theta-2 band between healer and client and a small phase synchrony in the theta frequencies
Bing-Canar H, Pizzuto J, Compton RJ.	Mindfulness-of-breathing exercise modulates EEG alpha activity during cognitive performance.	2016	Mindfulness of breathing	CDM-FA	Eyes open	10	Right mastoid	23 mindfulness group (9 males)<br>21 control group (5 males)	NA	Audio Listening and Stroop Task	Spectral (FFT)<br>ERP(Visual)	Higher alpha in mindful group listening the audio.<br>No change in alpha during stimuli processing.<br>Error Related Alpha Supression on stroop task was higher in mindful group than control.<br>ERP: no significant effects involving groups
Jo HG, Schmidt S, Inacker E, Markowiak M, Hinterberger T	Meditation and attention: A controlled study on long-term meditators in behavioral performance and event-related potentials of attentional control	2016	Various meditation centers	NA	NA	64	Fpz	20 meditators (8 males)<br>20 controls (8 males)	More than 5 years (Average 13.7 years)	Attentional Network Test that comprises cued detection (i.e. tasks involving different types of warning cues in advance) and a flanker-type paradigm (congruent and incongruent target stimuli).	ERP (Visual)	Higher parietal P3 amplitude in meditators during incongruent target in comparison with control.
Gao J, Fan J, Wu BW, Zhang Z, Chang C, Hung YS, Fung PC, Sik HH	Entrainment of chaotic activities in brain and heart during MBSR mindfulness training	2016	MBSR	CDM	Eyes closed	128	Left mastoid	11 (6 males)	8 weeks (some have previous experience in contemplative practices, but not reported the lenght of this experience)	Resting<br>Mindful breathing<br><br>2 weeks after begning of course and less than on month after the course	Spectral (FFT)<br>Non-linear (Wavelet entropy)<br>Source<br>Multimodal	FFT:<br>Higher alpha and beta and lower delta in meditation in comparison with resting. With no difference between early-stage and post stage.<br><br>Wavelet entopy:<br>Decreased during meditation compared with resting. No difference between early-stage and post-stage.<br>Correlation between hear and brain correlated during meditation, but not during resting.
Atchley R, Klee D, Memmott T, Goodrich E, Wahbeh H, Oken B.	Event-related potential correlates of mindfulness meditation competence.	2016	Mindfulness	CDM-FA	Eyes closed	32	Online recordings referenced a common mode sense (CMS) and driven right leg (DRL) electrodes, halfway between Cz and C3/4	42	Three groups:<br>Naive subjects<br>Novel to meditation (2.4 y SD 2.5)<br>Expert meditations (22.6y SD 13.2)	Oddball<br><br>Participants were instructed to respond to target tones with a button press in the first task (Tones), and then ignore the primed tones while breath counting.	ERP (Auditory)	The p3 amplitude was higher for target tones in meditators than in controls, on the other hand, meditators showed greater attenuation of the p3 amplitude during Breath Counting, when they were instructed to ignore the tones

Autors	Title	Year	Meditation	Meditation category (ADM,NDM,CDM)	Eyes open or closed?	Number electrodes	EEG reference	Number participants	Experience	Design	Method	Result
Tomljenović H, Begić D, Maštrović Z	Changes in trait brainwave power and coherence, state and trait anxiety after three-month transcendental meditation	2016	TM	NDM	eyes closed	8	NA	12 (7 males)	3 participants had prior experience (on average 2 months) all participants did a 3 months retreat	Before and after 3 months retreat<br><br>Supine position at resting (no meditating) with eyes closed (except in short eyes open epoch - excluded from this study)	Spectral (FFT) Coherence	Results: Changes in EEG patterns after meditation practice were found mostly in the theta band. An interaction effect was found on the left hemisphere ($p<0.10$). Theta power decreased on the left, but not on the right hemisphere. Increased theta coherence was found overall and in the central, temporal and occipital areas ($p<0.10$). Decrease in alpha power was found on channels T3 ($p<0.10$), O1 ($p<0.05$) and O2 ($p<0.10$). An interaction effect was found in the delta frequency band ($p<0.06$), too. Also, power decreased on channel O1 ($p<0.10$). In the beta frequency band, a decrease was found on channel O2 ($p<0.10$). Trait anxiety did not differ, but a decrease in state anxiety and cognitive worry was found ($p<0.05$).
Panda R, Bharath RD, Upadhyay N, Mangalore S, Chennu S, Rao SL	Temporal Dynamics of the Default Mode Network Characterize Meditation-Induced Alterations in Consciousness	2016	Raja yoga	CDM/FA	Eyes closed	31	FCz	20 meditators (all males)<br>20 controls (all males)	10-22 years (average 15.2 years)	9.24 min Resting state (meditators and control)<br>9.24 min Meditation (meditators)	Spectral (FFT)<br>Multimodal (fMRI)<br>Microstates	FFT:<br>Higher alpha, theta and beta in meditation when compared to resting (Meditators group only).<br><br>Microstates:<br>At rest meditator has increased duration and higher frequency of DMN microstate in comparison with controls.<br>During meditation duration and frequency where higher than in resting for meditators.<br>Positive correlation of DMN-microstate duration at rest and meditation with years of practice. Net increase (from resting to meditation) of duration of DMN-microstate was negatively with years of meditation.
DeLosAngeles D, Williams G, Burston J, Fitzgibbon SP, Lewis TW, Grummett TS, Clark CR, Pope KJ, Willoughby JO	Electroencephalographic correlates of states of concentrative meditation	2016	BreathMindfulness Discourse (Type of minfulness directed to breathing only)	CDM/FA	Eyes closed	116 EEG electrodes	left ear reference	12 meditators<br>12 paired naive participants	4 to 34 years of experience	Both groups had to follow randomized audio guided instructions on meditation(~29min), perception experimemt (~11min) and a steady-state response experiment (~18min). Baseline and mind-wandering was also recorded. Recording were done inside a Faraday cage	Spectral (Power)	Overall increase in alpha power independet of meditation depth. In deeper meditation states the authors observed decreased central scalp beta and central low gamma and increase in theta power. The difference in brain states and meditation depth was only seen in meditators
Biedermann B, de Lissa P, Mahajan Y, Polito V, Badcock N, Connors MH, Quinto L, Larsen L, McArthur G	Meditation and auditory attention: An ERP study of meditators and non-meditators	2016	Zen and Tibetan Mahayana practice	CDM<br>NDM	NA	30	Referenced to the online do the left mastoid and offline to the right mastoid	12 meditators<br>14 paired naive participants	10 to 35 years	Two blocks of 13 minutes of 666 randomized and jittered pure tones of 175ms duration (10ms rise and fall time).<br>One block was during meditation and the other during a non-meditation condition.<br><br>Each block presented 566 1000-Hz "standard" tones (85% of trials) interspersed with 100 1200-Hz "deviant" tones (15% of trials).	ERP (Auditory)	MMN was significantly larger in meditators than non-meditators in the meditative and non-meditative conditions.<br><br>N1 amplitude was significantly attenuated in non-meditators in the meditation condition but this was not observed for the meditators group.
Brandmeyer T, Delorme A	Reduced mind wandering in experienced meditators and associated EEG correlates	2016	Himalayan Yoga	CDM-FA/OM	Eyes closed	64	NA	Experts (9 males)<br>Non-experts (2 males)	Experts > 2h daily meditation at least 1 year<br>Non-experts (average 3.2h meditation weekly)	Question about meditation deep, mind wandering and how tired they are randonly spaced between 30 and 90s. Recorded from 45-90min. Minimum 30 probes per participant.	Spectal (Wavelets)	Experts had higher theta in frontal cortex and alpha over somatosensory cortex during meditation in comparison with mind wondering. No differences in non-experts.
Chandra S, Sharma G, Sharma M, Jha D, Mittal AP.	Workload regulation by Sudarshan Kriya: an EEG and ECG perspective	2017	Sudarshan Kriya Yoga	CDM-FA	NA	14	Right and Left mastoids	Randomly assigned between control and experimental groups<br>10: Control<br>15: Experimental<br>all males	Novices	Data acquired in a baseline and in a worload task (Low and High)<br>Pre and post meditation data acquired over a period of 30-90 days	Multimodal<br>Spectral (Wavelets)	Increase in the alpha and beta energies and root mean square of the EEG signal for the experimental group
Travis F, Parim N	Default mode network activation and Transcendental Meditation practice: Focused Attention or Automatic Self-transcending?	2017	TM	NDM	Eyes closed	32	Left and right earlobes	87 (45 males)	1 month - 5 years (Average: 1.3 years)	5 min Eyes closed resting<br>4 min attention task<br>5 min TM session	Source (eLORETA)	Results from TM vs resting eyes closed<br>Higher theta in BA25, 47 during TM<br>Lower in beta1 BA 20, 21 during TM
Braboszcz C, Cahn BR, Levy J, Fernandez M, Delorme A	Increased Gamma Brainwave Amplitude Compared to Control in Three Different Meditation Traditions	2017	Vipassana, Himalayan Yoga and Isha Shoonya	CDM-FA/OM	Eyes closed	64	right mastoid	16 (Vipassana) (11 males)<br>16 (Hymalayan Yoga) (14 males)<br>16 (Isha Shoonya Yoga) (14 males)<br>16 Controls (14 males)	Averages (in hours)<br>Hymalayan: 15457<br>Isha: 2625<br>Vipassana: 9201	Instructed Mind Wandering (20 min)<br><br>Meditation (each meditator practiced its own method and controls were asked to pay attention to their breath) (20 min)<br><br>For both only the last 10 min were analysed	Spectral (FFT)	Gamma power (60-110Hz) was higher in meditators over parieto-occipital electrodes with 2 conditions (meditation and mind wandering) combined, and Isha Yoga practitioners additionally increase over central and frontal electrodes.<br>When comparing meditation condition the gamma power in all meditator groups where higher than controls ouver fronta, midline and occipital electrodes site.<br>Inter-group comparison resulted in higher Gamma over 2-11Hz power ratio for Himalayan yoga group compared to control group, both during meditation and during mind wandering.<br><br>Correlation between hours of meditation and gamma power were found combining both conditions and with mind wandering, but not with gamma power during meditation only.<br><br>Alpha: All groups has alpha power higher than control group during meditation and mind-wandering, and Vipassana group were higher than all other groups.

Autors	Title	Year	Meditation	Meditation category (ADM,NDM,CDM)	Eyes open or closed?	Number electrodes	EEG reference	Number participants	Experience	Design	Method	Result
Nair AK, Sasidharan A, John JP, Mehrotra S, Kutty BM	Just a minute meditation: Rapid voluntary conscious state shifts in long term meditators	2017	Brahma Kumaris Rajayoga	CDM	Eyes open and closed	128	NA	25 - controls (meditation naive); 36 long term; 25 short term.	36 long term meditators: 14240 h meditation; 25 short term meditators: 1095 h.	Two rest and meditation protocols were performed. Each session contained rest eyes open, rest eyes closed. meditation eyes open and meditation eyes closed. A cognitive test was applied. Next, two more rest and meditation sessions were held.	Spectral (FFT)	Long term meditators showed enhanced upper theta power in the frontocentral and central electrodes during eyes open meditation and lower theta power and upper theta power to most regions except the frontal and frontopolar electrode during eyes closed meditation, compared to rest. There was increased lower alpha power in the frontal and occipital regions in short term meditators during eyes closed meditation compared to rest. Long term meditators showed increased lower alpha power during eyes open meditation vs rest, in the session after cognitive test. Considering eyes closed meditation vs rest there was enhanced lower and upper theta power in the session before cognitive test. Long term meditators had changes during subsequent states in delta, lower theta, lower alpha and lower beta power within each rest and meditation session compared to baseline rest and changes between rest and meditation in lower theta and lower beta power. Short term meditators and controls showed alterations in lower and upper theta and lower beta power during eyes closed.
Kornmeier J, Friedel E, Wittmann M, Atmanspacher H	EEG correlates of cognitive time scales in the Necker-Zeno model for bistable perception	2017	meditation types who have a dominant orientation toward awareness of the present moment (mindfulness meditation, Vipassana meditation, Soto Zen).	CDM-OM	Eyes open	32	Average of TP9 and TP10	12 experienced meditators (3 males)<br>15 controls (5 males)	at least 3 years of continuous practice and had practiced at least 2 h per week over the last 8 weeks.	Experiments 1 and 2: Spaced presentations of ambiguos necker Cube figures<br><br>Condition 1: Indicate when an inversion is perceived<br>Condition 2: Try to sustain one perception<br><br>Experiment 3: oddball	ERP (Visual)	Experiments 1 and 2: CPP- Centro-Parietal Positivity ~500ms after stimuli<br>Experienced meditators can volionally control duration of meta-stable percepts<br><br>Experiment 3: P300<br>No difference between latency of meditators and controls
van Lutterveld R, van Dellen E, Pal P, Yang H, Stam CJ, Brewer J	Meditation is associated with increased brain network integration.	2017	Effortless awereness (mindfulness based)	CDM-OM	Eyes open	128 + EOG	NA	16 novice (11 male)<br>16 experienced meditators (13 male)	novice: no practice or less than 20 hours in the entire lifetime<br>expericended:at least 30 min per day for at least 5 days per week over the past 5 years	30s baseline - participants viewed traid-adjectives and assessed if the words described themselves<br>3.5 min effortless meditation	Network (PLI)	experienced meditators had a maximum betweennes centrality in alpha significantly higher than novices.<br>Novice participants had a significantly lower diameter and average eccentricity in the alpha band,<br>Theta and beta bands showed no significant differences among groups<br><br>Functional network topology for the alpha band is more integrated in experienced meditiators than in novice
Jo HG, Malinowski P, Schmidt S.	Frontal Theta Dynamics during Response Conflict in Long-Term Mindfulness Meditators	2017	Mindfulness	CDM- FA/OM	Eyes Open	64 + EOG	Initial ref at FPz	22 meditadors (15 females)<br>23 matched controls (15 females)	average meditation period 13.1 years (SD = 5.9) and 247.8 min (SD=104.9) per week	flanker-type paradigm	ERP (Visual)<br>Synchronization (PLV)	Meditators responded significantly more accurately than controls especially in incongruent trials.<br>Increased theta power during incongruent trials compared to congruent in FCz. Meditatoes showed higher power in both incongruent and congruent trials than controls.<br>Enhanced synchrony during incongruent compared to congruent trials and significant interaction congruency x group (before response onset) in particular for functional connectivity between medial frontal cortex and motor cortex.
Sik HH, Gao J, Fan J, Wu BWY, Leung HK, Hung YS	Using Wavelet Entropy to Demonstrate how Mindfulness Practice Increases Coordination between Irregular Cerebral and Cardiac Activities	2017	Mindful breathing (MBSR)	CDM- FA/OM	Eyes closed	128	Average of all channels	11 (6 males)	novice	Before and after 8 week MBSR	Multimodal<br>Non-Linear (Wavelet Entropy)<br>Source (MNE)	The wavelet entropy of the brain EEG decreased during the MBSR mindful breathing state as compared to that during the closed-eye resting state. No difference in wavelet entropy during MBSR mindful breathing was found between the pretest and posttest.
Faber PL, Travis F, Milz P, Parim N	EEG microstates during different phases of Transcendental Meditation practice	2017	TM	NDM	Eyes closed	32	Mean reference	20	11.6 years; SD=16.1 years range 1 month-43.3 years	resting vs undirected mentation (UM) vs transcending (TR)	Microstates	UM compared to both resting and TR: higher coverage and occurrence of Class A, and lower coverage and occurrence of Class D<br><br>Compared to TR, UM was marked by significantly higher coverage and occurrence of Class C.<br><br>Microstates during TR did not differ from those during no-task resting before the meditation practice.
Lee YH, Hsieh YJ, Shiah YJ, Lin YH, Chen CY, Tyan YC, GengQiu J, Hsu CY, Chen SC	A cross-sectional evaluation of meditation experience on electroencephalography data by artificial neural network and support vector machine classifiers	2017	Focused breathing	CDM-FA	NA	6	earlober (A1 and A2)	10 Senior (7 males)<br>10 Junior (5 males)<br>10 Novice (3 males)	Senior: 10-30 years (Average: 17.10 years)<br>Junior: 1-7 years (Average=6.00 years)<br>Novice: no experience	Baseline: 4 blocks of 60s in counterbalanced random sequential of 30s open-eyes and 30s closed-eyes in each block.<br>Meditation: 3 runs of 5 minutes with 2 min rest between each meditation period.<br>FFT of Alpha were used to feature extraction. Peak, Frequency of peak and mean value were utilized. 2 normalized scales were testes (0-1, N1, and 0-10 N10)<br>SVM and ANN classifier were tested in this datatset	Spectral (FFT)<br>Classifier (SVM and ANN)	Best classification were obtained with an 1.5 minute window size, step of 2 seconds.<br><br>ANN had a higher than 98% of the true-positive rate is achieved for each group’s classification in 100 runs and <0.8% of false-positive rate is detected for misclassification, that is, 0.57% and 0.45% of the senior experienced meditators are assigned to junior and novice groups, respectively; 0.32% and 0.33% of junior experienced meditators are assigned to senior and novice groups, respectively; 0.77% and 0.40% of novice meditators are assigned to senior and junior groups, respectively.<br><br>SVM shows 100% of the true-positive rate and 0% of false-positive rate at classification under such a specific circumstance.

Autors	Title	Year	Meditation	Meditation category (ADM,NDM,CDM)	Eyes open or closed?	Number electrodes	EEG reference	Number participants	Experience	Design	Method	Result
Henz D, Schöllhorn WI	EEG Brain Activity in Dynamic Health Qigong Training: Same Effects for Mental Practice and Physical Training?	2017	Qigong	CDM-FA/OM	Eyes open/ Eyes closed	19	NA	25 (12 males)	At least 1h practice per week for 1 year and participation on a Qigong workshop (30h)	2 min resting with eyes open and 2 min resting with eyes closed<br>Before training and after 3 randomly selected interventions:<br>1) 15 min Qigong physical exercice<br>2) 15 min Qigong mental training (participants were required to perform the movement sequence mentally, applying the same breathing technique in the mental practice condition as in the physical training)<br>3) a video showing practitioners performing the Qigong practice of the physical and mental exercice	Spectral (FFT)	Theta: increased after physical and mental Qigong training compared to the video control condition. Differences between eyes-open, eyes-closed. Significant results for training x condition. Decreased in the eyes-closed condition compared to physical training, video control, and baseline rest. Theta power increased at frontal, central, parietal electrodes, and occipital electrodes.<br><br>Alpha-1: increased after physical and mental Qigong training compared to the control condition. No difference was obtained between physical and mental Qigong practice. Differences between eyes-open, eyes closed. Alpha-1 power was higher at central, and parietal electrodes than frontal, temporal, and occipital electrodes.<br><br>Alpha-2: higher after physical Qigong training compared to mental practice, video control. Differences between eyes-open, eyesclosed. Alpha-2 power at central, and parietal electrodes was higher than that of frontal, temporal and occipital electrodes. No difference between mental practice and video control.<br><br>Beta: increased in the video control condition, compared to mental practice, physical training. Differences between eyes-open, eyes-closed. Significant results for training x condition. Beta power central electrodes higher than frontal, temporal, parietal, and occipital electrodes.
Rodin E, Bornfleth H, Johnson M	DC-EEG recordings of mindfulness	2017	Yoga Nidra	NDM	Eyes closed	128	NA	10 (5 males)	NA	1h guided Yoga nidra	Spectral (FFT)<br>Source (Beamforming)	Delta (0.5-3.3Hz)<br>InfraSlow Activity (0.0002-0.1Hz)<br><br>Exploratory analysis. Show that are ICA components that starts negative and become posive during this 1 hour period, as well rhythmic wave durations from about 10 to 35 min. The topographic distribution was mainly in frontal near midline areas and the inferior protions of the hemispheres.<br><br>data demonstrate that the normal alert human brain does indeed produce rhythmic as well as more random activity well below the Infraslow band.
Travis F, Parim N, Shrivastava A	Higher theta and alpha1 coherence when listening to Vedic recitation compared to coherence during Transcendental Meditation practice.	2017	TM	NDM	Eyes closed	32	Left and Right ear lobes	37 (17 males)	Average: 34.0 years of TM and 28.6 years of TM-Shidhi	15 min TM practice<br>1h live recitation of Vedic literature	Coherence	Theta2 and alpha1 frontal, parietal, and frontal-parietal coherence were significantly higher when listening to Vedic recitation, than during TM practice.
Malinowski P, Moore AW, Mead BR, Gruber T	Mindful Aging: The Effects of Regular Brief Mindfulness Practice on Electrophysiological Markers of Cognitive and Affective Processing in Older Adults	2017	Mindfull breath awareness	CDM-FA	NA	64	Common Mode Sense (Additional Electrode)	18 per group<br>Meditator group: 3 males<br>Control group: 4 males	No experience. Two randomized groups, one received training and was asked to meditate regularly for a minimum of 10 min/day, 5 days/week. For 8 weeks, they attended 4 90 minutes group sections<br><br>Active control group performed brain training, which encompasses mental arithmetic calculations.	Meditators versus controls, with acquisitions of the Emotional-Counting Stroop task before and after training	ERP (Visual)<br>Source (VARETA)	N2 (270 to 340 ms) at FCz: increased N2 for meditators as compared to a relative (albeit not significant) decrease for controls. Furthermore, changes in the N2 amplitude from before and after training were positively correlated with reductions in reaction times<br><br>P300 in Pz did not have difference in groups.<br><br>VARETA: N2-related effect is primarily associated with changes in the right angular gyrus and other areas of the dorsal attention network (right superior, parietal lobe, right inferior temporal lobe, and the left lingual gyrus).
Schoenberg PLA, Ruf A, Churchill J, Brown DP, Brewer JA.	Mapping complex mind states: EEG neural substrates of meditative unified compassionate awareness	2018	Essence of mind	NDM	Eyes open and closed alternated	128	NA	20 (14 males)	Meditation capacity and advancement was rated by two independent teachers of the method (including the teacher who provided the meditation guidance within the experiment), via a 0–10 scaling system.<br><br>Score above 6 was necessary for participating on the study and average of 8.09 of meditation capacity and advancement	3 min of eyes open followed by 3 min eyes closed baseline<br>4 blocks of 15 min of meditation (4 different states of meditation) - 5 min guided meditation followed by 10 min silent meditation<br><br>2.5 min of each baseline and 10min of silent meditation were analysed	Source (LORETA)	Baseline vs Meditative stage 1:<br>Decreased activity in DMN, ACC, Insula, Precuneus, PCC, Superior and inferior parietal lobule in Alpha, Beta and Gamma<br>Decresed activity in mvPFC in Alpha<br><br>Increased current density magnitude in beta between meditation states (S1to S4)<br>Increased gamma1, gamma2 and beta current density from S1 to S4
Irrmischer, M., Houtman, S. J., Mansvelder, H. D., Tremmel, M., Ott, U., and Linkenkaer-Hansen, K	Controlling the temporal structure of brain oscillations by focused attention meditation	2018	Focused attention meditation	CDM-FA	Eyes closed	Experiment 1:128<br>Experiment 2: 32	common average	Experiment 1:8 (4 males)<br>Experiment 2: 20 meditators (10 males)<br>10 controls (5 males)	Experiment 1: minimum 5 years (M=18; SD=10.7)<br>Experiment 2: 1 mont to 33 years (M=10.8; SD = 10.2)	Meditation vs baseline	Non Linear (Detrended Fluctuation Analysis - DFA)	Temporal complexity of brain-activity fluctuations suppressed in experienced meditators.<br><br>A sustained practice over a period of a year increased the effect and affected normal waking brain dynamics, as reflected in increased LRTC during an eyes-closed resting state.
Sharma K, Chandra S, Dubey AK	Exploration of Lower Frequency EEG Dynamics and Cortical Alpha Asymmetry in Long-term Rajyoga Meditators	2018	Rajyoga	ADM	eyes open	61	CPz	31 Long Term meditators (all males)<br>31 Control group (all males)	Long Term meditators: > 10 years<br>Controls: No experience	Baseline (5 min)<br>Meditation (10 min); controls sit in a relaxed wakefulness condition with eyes open	Spectral (FFT, α asymmetry)	Results reveal high-band power in alpha and theta spectra in meditators. Cortical asymmetry calculated through EEG power was also found to be high in frontal as well as parietal channels.<br>No correlation was found between the total number of hours spent in meditation and any of the EEG band power variations.

Autors	Title	Year	Meditation	Meditation category (ADM,NDM,CDM)	Eyes open or closed?	Number electrodes	EEG reference	Number participants	Experience	Design	Method	Result
Wong KF, Teng J, Chee MWL, Doshi K, Lim J	Positive Effects of Mindfulness-Based Training on Energy Maintenance and the EEG Correlates of Sustained Attention in a Cohort of Nurses	2018	Mindfulness	CDM	Eyes open during activity and eyes closed during meditation	64	FCz	36 nurses (2 males)	No previous experience	Participants took a 8-week mindfulness based training (MBT) and data were acquired before and after the intervention.	ERP (Visual)	P300 and alpha ERD<br><br>Alpha power significantly decreases during meditation after intervention correlated with MBT attendance.<br><br>Small decrese in P300 over time in post intervention.<br>ERD in alpha was significantly negatively correlated with the number of MBT sessions arrended. Reduced alpha power synchronizaton with more sessions of MBT
Kakumanu RJ, Nair AK, Venugopal R, Sasidharan A, Ghosh PK, John JP, Mehrotra S, Panth R, Kutty BM	Dissociating meditation proficiency and experience dependent EEG changes during traditional Vipassana meditation practice	2018	Vipassana (Anapana + Vipassana + Mettabhavana)	CDM + ADM	Eye closed	128	average	Novice: 24 (12 males)<br>Senior: 22 (11 males)<br>Teachers: 21 (11 males)	Novice: 2-3 ten-day courses with less than 3 years of practice; (average 2.2years)<br>Senior: at least one long retreat (20-90 days - only allowed after several 10 days retreats), with more than 7 years of daily practice (average: 13.0 years)<br>Teachers: Instructor of Vipassana with daily practice of more than 7 years and several long retreats (average 16.3 years)	Pre-Rest Eyes Open (RO) and Eyes Closed (RC)<br>Anapana (Ana); Vipassana (Vipa); Metta<br>Post-Rest (RO and RC)	Spectral (FFT)<br>Non-linear (Permutation Entropy, Higuchi's Fractal dimension)	Seniors and Teachers (vs Novices) showed trait increases in delta (1–4 Hz), theta-alpha (6–10 Hz) and low-gamma power (30–40 Hz) at baseline rest; state-trait increases in low-alpha (8–10 Hz) and low-gamma power during concentrative and mindfulness meditation; and theta-alpha and low-gamma power during loving-kindness meditation.<br><br>Post-hoc analyses showed no differences between senior and teachers group in any bands and meditation types. However the novice group showed lower low-alpha and increased low-gamma when compared to the other groups<br><br>Median power spectra and hours of meditation were positively correlated with theta-alpha and negatively correlated with delta, beta and gamma bands.<br><br>Permutation entropy and Higuchi fractal dimension measures further dissociated high proficiency from duration of experience as only Teachers showed consistent increase in network complexity from baseline rest and state transitions between the different meditation states.
Barnes LJ, McArthur GM, Biedermann BA, de Lissa P, Polito V, Badcock NA	No meditation-related changes in the auditory N1 during first-time meditation	2018	Breath counting meditation	CDM - FA	NA	(FP1, FP2, Fz, FCz, Cz, CPz, Pz, Oz, O1, O2, AFz (GND)) + EOG	online reference to mastoid (M1), re-referenced to M2.	Experiment 1 - 8 participants<br>Experiment 2 - 12 participants<br>Experiment 3- 8 participants	No previous experience	Measurements done after one first-time meditation.<br>Experiment 1 - breath counting meditation and vizualization control condition	ERP (Auditory)	N100<br><br>Refute previous studies that found N100 in first-time meditators smaller during meditation than non-meditation condition.<br><br>Found the same result on experiments 1 and 2 but differ in 3. Found that the first result was due to the order of the conditions.<br><br>N100 reduction on the firsts studies could be a result of repeated exposure to stimuli.
Ghaderi AH, Moradkhani S, Haghighatfard A, Akrami F, Khayyer Z, Balcı F	Time estimation and beta segregation: An EEG study and graph theoretical approach	2018	Mindfulness - body scan	CDM-FA	Eyes open	19	Linked-ear	Experiment 1 (exploratory): 42 (25 males)<br>Experiment 2 (confirmatory): 17 (10 males)	NA	Study of time perception during meditation.	Spectral (FFT)<br>Network (Coherence)	Evaluation in delta and beta<br><br>FFT: No differences between two groups during rest state (control) condition<br><br>Graph: In the exploratory experiment, there was a difference in overestimator (OE) and underestimator (UE) groups in clustering coefficient in the beta sub-bands. In the beta2 and beta3 sub-bands, the UE group exhibited a higher clustering coefficient than the OE group. No significant difference was observed between the two groups in the beta1, high beta and delta bands. In the confirmatory experiment, striking differences of clustering coefficient were observed at the beta3 band respective to low thresholds.
Shaw L, Routray A	Topographical assessment of neurocortical connectivity by using directed transfer function and partial directed coherence during meditation	2018	Kriya Yoga Meditation	CDM	NA	64 - 8 used for analysis<br>F4, Fz, F3—frontal lobe, C3, C4 —central lobe, and P3, P4 and Pz—parietal lobe,	Earlobes	23 (17 males)	9.66 ± 15.31	Recordings only during meditation	Coherence (DTF, Partial directed coherence PDC)	PDC-based connectivity gives a better understanding of the non-symmetric relation in EEG obtained during Kriya Yoga meditation in comparison to DTF.
Harne BP, Hiwale AS	EEG Spectral Analysis on OM Mantra Meditation: A Pilot Study.	2018	OM chanting	CDM-FA	Eyes closed	16	right earlobe	23 (9 males)	No experience	2 min baseline recording (laying down with eyes closed)<br>30 min OM chanting (no eeg recording)<br>2 min post-chanting recording (laying down with eyes closed)	Spectral (FFT)	No specific band involved into the OM chanting effect.
Travis F, Valosek L, Konrad A 4th, Link J, Salerno J, Scheller R, Nidich S	Effect of meditation on psychological distress and brain functioning: A randomized controlled study	2018	TM	NDM	Eyes open	NA	Left and right earlobe	31 TM group<br>36 Control Group	No experience	Randomized clinical trial<br>4 instruction sessions; individual guidence once a month and weekly meetings.<br>EEG recordings pre and 4 months posttest.<br>Recording in 2 reaction time tasks (1 simple and one choice)<br>Coherence and Power where calculated on choice task (more demandant)	ERP (CNV)<br>Coherence<br>Spectral (FFT)	Broadband fronta (F3-F4) Coherence<br>CNV difference (CNVsimple-CNVchoice)<br>Frontal, central and parietal alpha relative power (Alpha power/Total power).<br><br>Frontal coherence, frontal, central and parietal relative alpha power, and the frontal and central simple/choice difference scores were converted to z-scores, compared to the normative database and summed to yield a single value for each person tested (Brain Integration Scale)<br><br>Increase in Brain Integration Scale

Autors	Title	Year	Meditation	Meditation category (ADM,NDM,CDM)	Eyes open or closed?	Number electrodes	EEG reference	Number participants	Experience	Design	Method	Result
Fucci E, Abdoun O, Caclin A, Francis A, Dunne JD, Ricard M, Davidson RJ, Lutz A	Differential effects of non-dual and focused attention meditations on the formation of automatic perceptual habits in expert practitioners	2018	Open Presence (OP) and Focused Attention (FA)	CDM-FA<br>NDM	Eyes open	128	Vertex	16 Long Term pratictioners (12 males)<br>15 control	> 10000h<br>Average: 28990<br><br>Controls 1 week (30 min per day)	Oddball in three conditions (3 runs):<br>Open Presence<br>Focused Attention<br>Reading<br>RE1-OP1-FA1, FA2-OP2-RE2, OP3-FA3-RE3	Spectral<br>ERP (Auditory)	Increase in alpha for both groups during meditation, between practices, OP has the most powerfull alpha activity in frontal ROI.<br>Increase in frontal gamma oscillatory power during PO meditation states in expert practitioners only when compared to RE. And significant increase in gamma oscillatory power in experts, compared to novices, during OP and FA, but not RE.<br><br>We did not find a relationship between spontaneous gamma activity and hours of meditation practice in expert practitioners.<br><br>ERP:Mismatch Negativity (MMN) and the Late Frontal Negativity (LFN)<br>We found a significant correlation between MMN and LFN amplitude and the frontal gamma activity in experts, but not novices and this correlation was marginally different between the two group.<br><br>Using a linear mixed model the authors showed that novices and experts modulates the amplitude of MMN differently in different meditative states
Dentico D, Bachhuber D, Riedner BA, Ferrarelli F, Tononi G, Davidson RJ, Lutz A	Acute effects of meditation training on the waking and sleeping brain: Is it all about homeostasis?	2018	Theravada and/or Tibetan Buddhist tradition	CDM-FA/OM<br>ADM	Eyes open and closed	256	mastoid	29 Long term practitioners(14 males)<br>24 control (12 males)	>3 years and<br>>3 one week retreat<br>Average 15.6 years<br><br>Control: no experience	resting waking and whole night sleep hd-EEG<br>At baseline;<br>After a full day of Vipassana practice ;<br>After a full day of Metta (LK) practice	Spectral (FFT)	LTM exhibited an increased power compared to baseline session over midline prefrontal and left centro-parietal electrodes in several frequency bands ranging from delta to low-gamma, with the higher spatial extent in the theta range.<br><br>This pattern was not present in controls
Hunter MA, Lieberman G, Coffman BA, Trumbo MC, Armenta ML, Robinson CSH, Bezdek MA, O'Sickey AJ, Jones AP, Romero V, Elkin-Frankston S, Gaurino S, Eusebi L, Schumacher EH, Witkiewitz K, Clark VP	Mindfulness-based training with transcranial direct current stimulation modulates neuronal resource allocation in working memory: A randomized pilot study with a nonequivalent control group	2018	Guided Mindfulness Meditation	CDM-FA/OM	NA	128	NA	16 Meditation Group<br>13 Control group (reading + sham tDCS)	NA<br>Excluded participants with more than 1 hour per week of cognitive training (including MBT)	Training of 4 weeks of meditation and tDCS<br>n-back at Baseline vs post test	Multimodal (tDCS)<br>ERP (Visual)<br>Spectral (Wavelets)	P300 amplitude increased in Meditators group in comparison with control group at post-training in frontal sites and decreased at frontal electrodes.<br><br>P300 theta power: eMBT group was increased compared to the Control group and to eMBT baseline when collapsing across WM load conditions for Pz (mid line). And eMBT group has a decreased Fz theta power in 3-back after training when compared to control.
Van der Lubbe RHJ, De Kleine E, Schreurs KMG, Bohlmeijer ET	Does mindfulness training modulate the influence of spatial attention on the processing of intracutaneous electrical stimuli?	2018	Mindfulness (MBSR)	CDM-FA	eyes open	61 + EOG	NA	28:<br>15 in the pre-post group (immediately before and after MBSR)<br>13 in the post-post2 group (immediately after MBSR and 8 weeks after)	No previous experience (8 weeks intervention)	Go/NoGo with visual and electrical Stimulus	ERP (Visual/Electrical stimulus)	Alpha power was clearly lateralized due to spatial attention and several ERP components (N130, N180, P340) were modulated by spatial attention, as expected in this experimental setup but no support was found for an influence of the MBSR training and no significant effects or interaction with Individual Training Time.<br>Analyses revealed that individual differences in training time modulated some of the observed effects, but no support was found for an influence on attentional orienting.
Schöne B, Gruber T, Graetz S, Bernhof M, Malinowski P	Mindful breath awareness meditation facilitates efficiency gains in brain networks: A steady-state visually evoked potentials study	2018	Mindful breath awareness meditation	CDM -FA/OM	eyes open	128	Two additional electrodes were used as reference and ground electrodes, CMS and DRL.	17 in meditation group 17 in progressive muscle relaxation	naive	participants were randomly assigned to 8 weeks of meditation or progressive muscle relaxation. Experiment compared both groups (pre-post intervention) during multiple object tracking with electrophysiological recording of steady-state visual evoked potentials.	EP (Visual)	reduced SSVEP-amplitudes (500 ms to 6800 ms) after movement onset and in the three time windows: T1 (500-2000 ms), T2 (2000 –4750 ms) and T3 (4750 –6800 ms) in meditation group after the training compared to progressive muscle relaxation.
Lee D, Kang DH, Ha NH, Oh CY, Lee U, Kang SW	Effects of an Online Mind-Body Training Program on the Default Mode Network: An EEG Functional Connectivity Study	2018	Mind-Body Training	CDM- FA/OM	Eyes closed	19	common average	14 in intervention<br>15 in waiting list control group<br><br>all females	Intervention group participated online in 8 minute sections, once a day, 5 days a week during 4 weeks	Recordings on resting state	Source (beamforming)<br>Network (Imaginary Coherence)	global DMN network strengths in the theta and alpha frequency bands in meditators and not in controls
Luft CDB, Zioga I, Banissy MJ, Bhattacharya J	Spontaneous Visual Imagery During Meditation for Creating Visual Art: An EEG and Brain Stimulation Case Study	2019	stabilizing and analytical meditation	CDM	Eyes closer	64	NA	1	10 years	baseline eyes open and closed<br>10 meditation sessions in 5 non consecutive days<br>tACS applied in alpha, gamma and sham version in 3 days	Multimodal (tACS)<br>Spectral (FFT)	Gamma increase in analytic meditation at frontal and occipital sites.<br>Alpha tACS related with sharper, shorter and increasef number of visions during meditation
Telles S, Singh D, Naveen KV, Pailoor S, Singh N, Pathak S	P300 and Heart Rate Variability Recorded Simultaneously in Meditation	2019	meditative focusing and defocused meditation	CDM- FA/OM	eyes open	1 (Cz)	linked earlobes	47 (all males)	> 24 months + 3 months intervention	4 interventions (20 min) in random order and diferent days:<br>1) nonmeditative focused thinking<br>2)Random thinking<br>3) Meditative focusing<br>4) Defocused meditation<br><br>Oddball task (5 min) before and after intervention	ERP (Auditory)	There was an increase in the P300 peak amplitude after meditative focusing, defocused meditation and a decrease after random thinking.<br>P300 peak latency reduction after defocused meditation.

Autors	Title	Year	Meditation	Meditation category (ADM,NDM,CDM)	Eyes open or closed?	Number electrodes	EEG reference	Number participants	Experience	Design	Method	Result
Isbel BD, Lagopoulos J, Hermens DF, Summers MJ	Mental training affects electrophysiological markers of attention resource allocation in healthy older adults	2019	Mindfulness Meditation	CDM-FA	NA	9	average mastoid	41 meditation group (45% males)<br>28 active control group (25% males)	No experience (8 weeks intervention)	Oddball	ERP (Auditory)	Decreased N2 amplitudes at frontal and central regions during an auditory oddball task after training in both groups.<br>Only mindfulness group showed reductions in frontal N2 and P3 latency.
Miyashiro D, Toyomura A, Haitani T, Kumano H	Altered auditory feedback perception following an 8-week mindfulness meditation practice	2019	Mindfulness Meditation	CDM-FA/OM	NA	64	Left earlobe	15 Meditation Group (4 males)<br>15 Control (6 males)	No experience (8 weeks intervention)	Vocalization task - vocalize /a/ approximately 100 times, with 200ms delay feedback (~2min)<br>Listening task - participants listening their voices (~2min)<br><br>2 Recordings session (pre and post intervention). In each session there are 2 blocks separated by 16 min of meditation or listening to news.	ERP (Auditory)	In the vocalization task there was a N1 increase amplitude after meditation session in post-intervention session.<br>In the listening task, no difference was found.
Pozuelos JP, Mead BR, Rueda MR, Malinowski P.	Short-term mindful breath awareness training improves inhibitory control and response monitoring	2019	mindful breath awareness meditation	CDM-FA	NA	64	NA	21 Meditation Group (10 males)<br>15 Waitlist control group (9 males)	No experience (3 week intervention)	Go/NoGo	ERP (Visual)	Meditation training enhanced the N2 event-related potential in No-Go trials and the error-related negativity (ERN) after error responses. The changes in the ERN were correlated with the accumulated amount of meditation time. No group difference in P300.
Kakumanu RJ, Nair AK, Sasidharan A, John JP, Mehrotra S, Panth R, Kutty BM	State-trait influences of Vipassana meditation practice on P3 EEG dynamics	2019	Vipassana	CDM	Eyes open	128	NA	22 novices (11 males)<br>21 Senior (10 males)<br>16 teachers (8 males)	Averages:<br>Novices: 989h<br>Senior: 10520h<br>Teachers: 14648h	Gamified multilevel oddball	ERP (Visual)<br>Spectral (of ERP)<br>Coherence	Novices were very different from the long-term groups showing significant differences in the scalp measures of spectrum, ERSP and ITC at the theta-alpha and gamma1 bands.<br><br>Nov processed rare and frequent stimuli differently for ERSP in the theta2 band as well as in gamma1 power while the other two groups did not.<br><br>Novices and Teachers have differences for all the measures (ERP, spectra, ERSP and ITC) at Pz, differences only in spectra between Novices and Senior and no differences between Senior and Teacher.<br><br>Scalp plots for spectra, ERSP and ITC also showed a wider range of changes between Novices and Teachers (encompassing theta-alpha, beta1 and gamma1 bands) whereas there were no differences between Novices and Senior for ITC, and primarily during alpha bands for spectral measures. Senior and Teacher were different in terms of alpha1 synchrony for frequent stimuli.
Lasaponara S, Glicksohn J, Mauro F, Ben-Soussan TD	Contingent negative variation and P3 modulations following mindful movement training	2019	Quadrato Motor Training	-	Eyes open	64	Cz	23 (8 males)	No experience	Simple Response Time (SRT)<br>Choice Response Time (CRT)<br><br>Tasks performed before and after 7 min meditation session	ERP (Visual)	Increased CNV for SRT and decreased for CRT after meditation practice<br>No P3 amplitude difference for SRT and increased dor CRT after meditation practce
Sharma H, Raj R, Juneja M	EEG signal based classification before and after combined Yoga and Sudarshan Kriya	2019	Sudarshan Kriya (SK)	CDM-FA	Eye closed	32	NA	50, all males<br>25 - Control Group<br>25 - Intervention Group	Naive	3 months of Yoga and SK meditation (1h/Day)<br>10 min (lying down)	Spectral (Wavelets - Daubechie)<br>Classification (Artificial Neuronal Network)	87.2% of accuracy in distinguishing meditators and non-meditators.
Bailey NW, Freedman G, Raj K, Sullivan CM, Rogasch NC, Chung SW, Hoy KE, Chambers R, Hassed C, Van Dam NT, Koenig T, Fitzgerald PB	Mindfulness meditators show altered distributions of early and late neural activity markers of attention in a response inhibition task	2019	Mindfulness Based	CDM - FA	Eyes open	64	Between Cz and CPz	34 Meditators (13 males)<br>28 control group (11 males)	2 years or more (except for 3 meditators)	Go/NoGo (emotional faces)	ERP (Visual)<br>Source (sLORETA)<br>Global Field Potential (RAGU)<br>Microstates (RAGU)	P300/ P100<br>Controls has different amplitudes for P300 in Go than in No-Go trials, while meditotors showed no differences.<br>P300 was more frontally distributed in meditators than in controls<br>Pre-C1 topography were different between groups (differences in antecipatory activity)<br>P300 has more fronto-central positivity in both Go and No-Go trials (altered attentiona function)<br>N2 presented no differences (thought to reflect inhibition and conflict monitoring)<br>Microstates: Microstate related to pre-C1 (microstate 2) began earlier in meditators comparade to controls. Microstates (5 and 6), reflecting P300, show that meditators spend more of the P300 period in frontally dominant topographies compared to controls.<br>Loreta: Similar distributions of activity between groups in both the per-C1 and P300 periods. Diference maps showed that meditators has more pre-C1 activity in right temporal and parietal regions, and P300 activity widespread in central frontal and parietal regions.
Zhang W, Ouyang Y, Tang F, Chen J, Li H	Breath-focused mindfulness alters early and late components during emotion regulation	2019	Breath-focused mindfulness	CDM - FA	Eyes open	64	Left-mastoid	20 (11 males)	naive	Participants listen recording of BFM for three minutes before experimental procedure.<br><br>Participants performed 60 blocks of task. Each block has 3 images (negavite, neutral and positive valence) x 2 task (View or Focus).	ERP (Visual)	Chinese Affective Picture System (CAPS)<br><br>BFM attenuated P1, N2, and LPP amplitudes for positive and negative pictures but not for neutral pictures. P1 amplitudes for emotional minus neutral pictures correlated with individual differences in focus attention measured by the Attentional Control Scale, while N2 amplitudes for emotional minus neutral pictures correlated with individual differences in trait mindfulness measured by the MAAS.
Nyhus E, Engel WA, Pitfield TD, Vakkur IMW	Increases in Theta Oscillatory Activity During Episodic Memory Retrieval Following Mindfulness Meditation Training	2019	mindfulness meditation	CDM-FA	NA	64	NA	20 meditation group (10 males)<br>20 wait list group (7 males)	No experience (4 weeks intervention)	Episdic Memory Task<br>Pre and Post intervention	Spectral (Wavelets-Morlet)	Theta power (baseline corrected, 800ms before stimulus) was greater post-training than pre-training in right frontal and left parietal channels for the meditation group and changes in FFMQ scores correlated with changes in theta oscillations in right frontal channels

Autors	Title	Year	Meditation	Meditation category (ADM,NDM,CDM)	Eyes open or closed?	Number electrodes	EEG reference	Number participants	Experience	Design	Method	Result
Andreu CI, Palacios I, Moënne-Loccoz C, López V, Franken IHA, Cosmelli D, Slagter HA	Enhanced response inhibition and reduced midfrontal theta activity in experienced Vipassana meditators	2019	Vipassana	CDM	NA	32	NA	24 meditators<br>20 non-meditators	At least 1 year, 3 times a week, 1h each time. Average 5.1 years	Go/NoGo Emotional pictures	ERP (Visual)<br>Spectral (Multitaper)	ERP:<br>No changes in ERP indices of response inhibition, as indexed by the amplitude of the N2 and P3 components.<br><br>Spectral:<br>Decrease in midfrontal theta activity in Nogo vs. Go trials in the meditators compared to controls.
Kornmeier J, Friedel E, Hecker L, Schmidt S, Wittmann M	What happens in the brain of meditators when perception changes but not the stimulus?	2019	meditation types with a dominant orientation toward awareness of the present moment (mindfulness meditation, Vipassana meditation, Soto Zen).	CDM-OM	Eyes open	32	Average of TP9 and TP10	12 experienced meditators (3 males)<br>15 controls (5 males)	at least 3 years of continuous practice and had practiced at least 2 h per week over the last 8 weeks.	Similar to Go/NoGo but with ambiguous figures (Necker). Participants indicated if perception changed or remained.<br><br>They measured passive viewing and another condition where participant tried to hold perception for as long as possible	ERP (Visual)<br>Source (MSP)	Source:<br>Contrast of reversal and stability trials<br><br>Frontal Negativity (around 160ms after stimulus onset) present only in Meditators show increased activity in middle/posterior cingulate cortex and left supplementary motor area. In more frontal sites, right medial prefrontal cortex showed increased reversal-related activity.
Baquedano C, Lopez V, Cosmelli D, Lutz A	Electrophysiological evidence of the differential modulation of approach-related processes toward attractive foods by immersive or mindful viewing conditions	2019	NA	NA	NA	64	NA	25 (12 males) no meditators<br>25 (5 males) meditators	6 months - 6 years (average 1415 hours of practive)	Approach Avoidance Task (120 food images)<br>Conditions Mindfull or Immersed	ERP (Visual)	The P3 amplitude at Pz was greater for the mindful then for the immersed condition. As main effect, P300 amplitude was greater for approach than for the avoidance responses.<br><br>LPP1 at the parieto-occipital ROI (PO7, PO3, PO8, and PO4), where greater for approach than avoidance responses. The non-meditator group has greater amplitude for approach than for avoidance. For meditator group, LPP1 amplitudes for approach and avoidance were not different.<br><br>LPP2 at parietal ROI (P1, Pz, P2), for the control group, the LPP2 amplitude for approach responses was greater than for avoidance responses. For the meditator group, the LPP2 amplitude for approach responses was not greater than for avoidance responses. In summary individuals with more expertise in mindfulness meditation engaged in less late affective reappraisal during mindfulness than during immersion, as measured by lower amplitude in the late positive potential (LPP).<br><br>The approach bias toward attractive food was correlated with N2 amplitude, a marker of response inhibition, and the regulation of this bias by the mindful instruction compared to the immersed instruction was associated with a modulation of the visual N1 amplitude, a marker of early selective attention. In mindful condition at occipital ROI (O1, Oz, and O2), food type by response interaction, with greater N1 amplitude for avoidance then for
Savostyanov A, Tamozhnikov S, Bocharov A, Saprygin A, Matushkin Y, Lashin S, Kolpakova G, Sudobin K, Knyazev G	The Effect of Meditation on Comprehension of Statements About One-Self and Others: A Pilot ERP and Behavioral Study	2019	Shamata	CDM-FA	Eyes open	130	Cz	17 control (10 males)<br>18 short-term (11 males)<br>18 long term (11 males)	short-term: 3-5 years<br>long-term:10-30 years	Serial reaction time task (SRTT) with sentences with half of them with grammatically wrong sentences. There where 5 hidden groups of sentences:<br>1) describing agression of a participant<br>2) describing sentences of other people<br>3) describing anxiety of a participant<br>4) describing anxiety in other people<br>5) neutral sentences about inanimate objects	ERP (Visual)	Higher amplitude of P300 peak in frontal and left temporal scalp regions for long-term meditators in comparison with both intermediate and control groups. The latency of P300 and P600 in left frontal and temporal regions positively correlated with TPT, whereas the amplitude of P300 in these regions had a negative correlation with TPT.
JR Payne, O Baell, H Geddes, B Fitzgibbon, M Emonson, AT Hill, NT Van Dam, G Humble, PB Fitzgerald, NW Bailey	Experienced meditators exhibit no differences to demographically-matched controls in theta phase synchronisation, P200, or P300 during an auditory oddball task	2020	mindfulness	CDM-FA/OM	Eyes open	64	reference positioned between CPz and Cz	22 - meditators<br>22 - controls	Medidators - mean of 9.09 years of experience and 6.35 hours of current practice per week<br><br>Controls - less than 2 hours of meditation experience in a lifetime	-Go/Nogo task<br>-Dichotic listening<br>oddball task (Lutz et. al 2009)	ERP (Auditory)<br>Coherence (inter-trial coherence in Theta)	No difference between meditators and controls were found on the theta phase synchronization, P200 or P300 using Topography Consistency Tests
Anthony P. Zanesco, Brandon G. King, Chivon Powers, Rosanna De Meo, Kezia Wineberg, Katherine A. MacLean, & Clifford D. Saron	Modulation of Event-related Potentials of Visual Discrimination by Meditation Training and Sustained Attention	2019	shamata and metta	CDM	Eyes Open	88	average referenced	60 experienced meditatios.<br>30 did a first meditation retreat<br>30 stayed in a waiting list and did the retrat after 3 months	The participatns had experience:<br>-number of prior meditation retreats- mean = 14,<br>-minutes of daily meditation practice - mean = 55<br>-estimate hours of lifetime experience mean = 2,610 total hours<br><br>Both groups underwent in a 3-months retreat	continuous performance task (CPT) at the beginning, middle an end at the meditation intervention	ERP (Visual)	-CARTOOL for calculating GFP<br>-RAGU for Topographic ANOVA<br><br>On retreat 1 the difficulty of the task was calibrated to match the participants' current discrimination threshold (they increased their ability) and no changes in ERP were observed.<br><br>On retreat 2 the difficulty of the task was calibrated only at pre retreat test and kept constant for mid and post retreat tests and changes on N1 (faster and greater GFP amplitude) and P300 GFP amplitude increased for hits. The decline of GFP amplitude within was attenuated on Retreat 2.

Autors	Title	Year	Meditation	Meditation category (ADM,NDM,CDM)	Eyes open or closed?	Number electrodes	EEG reference	Number participants	Experience	Design	Method	Result
Eichel K, Stahl J	Error processing and mindfulness meditation in female students	2020	Focused attention meditation, open monitoring and body scan	CDM-FA/OM	NA	61	left mastoid	42 (42 females)<br>22 meditation group<br>20 Progressive Muscle Relaxation	No experience (4 weeks intervention)	5 min baseline<br>50 min modified Simon task and an error detection paradigm (error-related negativity and error positivity).<br>(11 blocks in total)	ERP (Visual)	No difference on error negativity or error-related negativity (Ne/ERN) and and error positivity (Pe) when comparing meditation and progressive muscle relaxation groups
Jiang H, He B, Guo X, Wang X, Guo M, Wang Z, Xue T, Li H, Xu T, Ye S, Suma D, Tong S, Cui D	Brain-Heart Interactions Underlying Traditional Tibetan Buddhist Meditation	2020	Samatha and Vipassana	CDM-FA/OM	Eyes closed	63	Common average	60 Tibetan Buddhist monks<br>25 male volunteers<br><br>all males	at least 2 h a day for 5–35 years (18.15 ± 8.25 years).	baseline vs meditation	Source (DICS, LCMV)<br>Network (Imaginary Coherence, Cross Frequency Directionality)<br>Multimodal	meditation vs resting (state)<br>a cluster encompassing the ACC bilaterally and extending through the superior medial frontal gyrus.<br>decreased mean connectivity in the gamma band in the frontal-limbic network<br><br>meditators rest vs controls rest (trait):<br>2 separate clusters containing multiple regions: one extending across the PCC and precuneus bilaterally and another in the right mPFC<br>decreased parietal-central and parietal-limbic network connectivity in the theta band<br><br>Interaction between theta and gamma<br>Power of gamma oscillations resets the phase of theta oscillations during meditation. After averaging the CFD in the theta and gamma frequency ranges, we found that the gamma to<br>theta CFD was significantly different between monk meditation and monk rest but not between monk rest and control rest
Katyal S, Hajcak G, Flora T, Bartlett A, Goldin P	Event-related potential and behavioural differences in affective self-referential processing in long-term meditators versus controls	2020	Ananda Marga	NDM	Eyes open	32	NA	13 (9 males)<br>Meditation Group<br>15 (9 males) Control Group	More than 10 years; Average 32.2 years	self-referential encoding task (SRET)	ERP (Visual)	early-Late Positive Potential was higher in crontrols for unpleseant vs no difference in meditators.
Chan RW, Alday PM, Zou-Williams L, Lushington K, Schlesewsky M, Bornkessel-Schlesewsky I, Immink MA	Focused-attention meditation increases cognitive control during motor sequence performance: Evidence from the N2<br>cortical evoked potential	2020	Yoga Nidra	CDM-FA	Eyes closed	64	NA	29 (11 males):<br>12 (21 meditation sessions Group)<br>9 (1 meditation session Group)<br>8 (no meditation Control group)	No previous experience	21 days of Guided Meditation (21 FA Meditation group)<br>19 days of Listening Audio non-meditation related + Listening guided meditation before second test (1 FA meditation group)<br>19 days of Listening Audio non-meditation related (Control Group)	ERP(Visual)	N2 ERP on a Serial Reaction Time Task (SRTT)<br>The group with 21 sessions demonstrated a more pronounced N2 (that is closely related to cognitive control process and its ability to predict behavioral measures) over majority of anterior and central regions of interests during SRTT compared to the other groups.
Vivot RM, Pallavicini C, Zamberlan F, Vigo D, Tagliazucchi E	Meditation Increases the Entropy of Brain Oscillatory Activity	2020	Vipassana, Himalayan Yoga and Isha Shoonya	CDM-FA/OM	Eyes closed	64	right mastoid	16 (Vipassana) (11 males)<br>16 (Hymalayan Yoga) (14 males)<br>16 (Isha Shoonya Yoga) (14 males)<br>16 Controls (14 males)	Averages (in hours)<br>Hymalayan: 15457<br>Isha: 2625<br>Vipassana: 9201	Instructed Mind Wandering (20 min)<br><br>Meditation (each meditator practiced its own method and controls were asked to pay attention to their breath) (20 min)<br><br>For both only the last 10 min were analysed	Non-linear (sample Entropy)<br>Coherence<br>Classification (Random Forest)	Vipassana resulted in the highest entropy increases, predominantly in the alpha and low/high gamma bands.<br>All meditation traditions increased the global coherence in the gamma band, but also stabilized gamma-range dynamics by lowering the metastability.<br><br>Machine learning classifiers could successfully generalize between certain pairs of meditation traditions based on the scalp distribution of gamma band entropies.
Yoshida K, Takeda K, Kasai T, Makinae S, Murakami Y, Hasegawa A, Sakai S.	Focused attention meditation training modifies neural activity and attention: longitudinal EEG data in non-meditators	2020	Focused Attention Meditation	CDM-FA	Closed eyes	20	Nose tip	17 meditation group (7 males)<br>20 control group (10 males)	Naive (8 week program)	8 week meditation training vs<br>8 week relaxation training<br>three-stimulus oddball (2 runs of 4min)	ERP (Auditory)<br>Synchrony (Phase Synchrony Index - Morlet Wavelet)<br>Spectral (FFT)	P300: Higher amplitude during oddball task and shorter reaction time in meditators group than in control at post training.<br>PSI: negative correlation between F4-Oz theta PSI during meditation and P300 amplitude during oddball task. Positive correlation between F4-Pz theta PSI during meditation and P300 amplitude during oddball task. Both in meditatiors but not control group.
